# What is known about population level programs designed to address gambling-related harm: rapid review of the evidence

**DOI:** 10.1186/s12954-024-01032-8

**Published:** 2024-06-18

**Authors:** Samantha Clune, Deepika Ratnaike, Vanessa White, Alex Donaldson, Erica Randle, Paul O’Halloran, Virginia Lewis

**Affiliations:** 1 Australian Institute for Primary Care & Ageing (AIPCA), La Trobe University, Melbourne Campus, Victoria, 3086 Australia; 2 Centre for Sport and Social Impact (CSSI), La Trobe Business School, La Trobe University, Melbourne Campus, Victoria, 3086 Australia; 3 School of Psychology and Public Health, La Trobe University, Melbourne Campus, Victoria, 3086 Australia

**Keywords:** Gambling, Public health, Harm reduction

## Abstract

**Background:**

Gambling and gambling-related harm attract significant researcher and policy attention. The liberalisation of gambling in most western countries is strongly associated with a marked rise in gambling activity and increases in gambling-related harm experienced at the population level. Programs to address gambling-related harm have traditionally focused on individuals who demonstrate problematic gambling behaviour, despite clear evidence of the effectiveness of a public health approach to high-risk activities like gambling. Little is known about the availability or efficacy of programs to address gambling-related harm at a population level.

**Methods:**

The Victorian Responsible Gambling Foundation commissioned a rapid evidence review of the available evidence on programs designed to reduce gambling-related harm at a population level. The review was conducted using a public health and harm reduction lens. MEDLINE, ProQuest Central and PsychInfo databases were searched systematically. Included studies were published in English between 2017 – 2023 from all countries with gambling policy contexts and public health systems comparable to Australia’s; included primary data; and focused on primary and/or secondary prevention of gambling-related harm or problems.

**Results:**

One hundred and sixty-seven articles were eligible for inclusion. Themes identified in the literature included: risk and protective factors; primary prevention; secondary prevention; tertiary prevention; target population group; and public health approach. The evidence review revealed a gap in empirical evidence around effective interventions to reduce gambling-related harm at the population level, particularly from a public health perspective.

**Conclusions:**

Addressing gambling-related harm requires a nuanced, multi-layered approach that acknowledges the complex social, environmental, and commercial nature of gambling and associated harms. Moreover, evidence demonstrates community programs to reduce gambling-related harm are more successful in reducing harm when based on sound theory of co-design and address the social aspects that contribute to harm.

## Background

Gambling is entrenched leisure activity in Australian society, sanctioned and regulated by government. Approximately 35% of all Australian adults spent money in a ‘typical month’ on one or more gambling activities [1]. The estimated per capita personal spend is $133 for a typical month [2]. In the Australian state of Victoria, gambling activity associated with poker machines was estimated to be $2.7 billion for 2018–2019 [1,2], with the estimated national spend at $12.7 billion for the same period. This spend on gambling activities means Australia has been described as the gambling capital of the world [3]. The negative impact, or harms, associated with gambling (i.e. gambling-related harm) are complex but well understood [4]. Gambling-related harm extends beyond the person who gambles to ‘affected others’ and the community. Harms are seen across financial, emotional, health, cultural, workplace and criminal domains with increasing lasting impact from a temporal perspective [4].

Attempts to understand and address gambling-related harm have mainly focused on individuals who gamble and their problematic behaviours and/or addiction issues. Language used to describe harm experienced as a result of gambling has varied over time and demonstrates a shifting perspective in terms of where harm originates and where responsibility may lie. The dominant discourse has been around ‘responsible gambling’ and ‘problem gamblers’, which propagates the notion of personal responsibility and problematic or dangerous personal characteristics and behaviours [5]. Critics of the responsible gambling view of gambling harm argue that this individualistic approach fails to appreciate the broad and complex nature of gambling-related harm beyond the individual who gambles [6]. Calls to adopt a public health approach, informed by principles of harm reduction, to gambling and gambling-related harm have resounded for many years [6,7]. Harm reduction, or harm minimisation, is generally associated with reducing adverse impacts from addiction practices where abstinence is not a desired outcome but adverse health outcomes are reduced as much as practicable [8]. Crucially, language has shifted from an individual focus—the ‘problem gambler’ demonstrating ‘problem gambling’ who should engage in ‘responsible gambling’—to a broader, more nuanced view of where and how gambling-related harm is experienced [4]. Specifically, Langham et al. (2016) take a social view of gambling-related harm to define it as:

Any initial or exacerbated adverse consequences due to an engagement with gambling that leads to a decrement to the health or wellbeing of an individual, family unit, community or population. [4]

A public health approach is helpful to design and deliver programs to address gambling-related harm when a social view of gambling is adopted, [6]. Programs designed using a public health approach can focus on primary, secondary or tertiary prevention, or incorporate aspects of each level of prevention [9]. Public health approaches include preventing harm, early intervention to minimise likelihood of harm, and treating harm [9].

Legislation mandates in some Australian states requires an identified proportion of all gambling revenue is dedicated to treating and preventing gambling-related harm [10,11], alongside establishing quasi-autonomous non-government agencies (QANGOs) responsible for reducing gambling-related harm [12]. This rapid review was undertaken to inform the creation of a programming framework for the Victorian Responsible Gambling Foundation that has operated for over 10 years, in Victoria, Australia to reduce the social, health and economic costs of gambling-related harm via a public health approach.

This rapid review focused on identifying available evidence about effective public health responses to gambling-related harm by examining international, English language, peer reviewed literature on:

- What is known about programs designed to address gambling-related harm?
- What evidence is there of the impact of gambling-related harm prevention programs on priority population groups?
- What is known about the risk and protective factors for gambling-related harm, particularly in priority population groups?

The key purpose of this review was to inform what action could be taken at the population health level.

## Methods

### Search strategy

A rapid evidence review is an assessment of available evidence related to a current policy or practice issue [13,14]. It is a form of knowledge synthesis in which components of a systematic review are simplified to expedite the process. As such, we adopted the procedure as described by Haby et al. [14]. A mnemonic was used to help construct the search strategy (PICO: Population; phenomenon/Intervention of interest; Context; and Outcome) [15]. All reporting was undertaken using the Preferred Reporting Items for Systematic Reviews and Meta-Analysis (PRISMA) guidelines [16]. Given the public health focus of this review, we systematically searched three key databases (MEDLINE, ProQuest and PsycInfo). We also conducted a grey literature search, which will not be reported.

We used a combination of terms for searching databases to elicit responses to the research questions, using the following key word combinations:

(gambling OR responsible gambling OR problem gambling) AND (harm reduction OR harm minimisation OR prevention) AND (access AND exposure AND community AND stigma AND uptake AND capability).

Searches were limited to literature published between January 2017 to November 2023 from countries with gambling policy contexts and public health systems similar to Australia, like the UK, USA, Canada, and New Zealand. These countries all have a hybrid welfare and market model of health care delivery and/or a similar proportion of GDP spend on public health [17].The decision to limit the search timeframe was a pragmatic one. Rapid evidence reviews often require tighter windows for age of evidence, and an existing systematic review addressing similar research questions conducted in 2017 further influenced our selection of search timeframe.

### Inclusion and exclusion criteria

Studies were considered for inclusion if they: referred to prevention programs that primarily focused on gambling-related harm or problems; were focused on primary, secondary and/or tertiary prevention of gambling-related harm; and/or included primary data.

Studies were excluded if they were: studies on clinical interventions for individuals with a diagnosis of pathological gambling, as per Diagnostic and Statistical Manual of Mental Disorders (Fifth Edition) [18]; or described non-applied research.

For this review, we adopted Langham’s (2016) definition of gambling-related harm described above. All articles that made specific reference to programs designed to address gambling-related harm were considered for inclusion. Given the paucity of available evidence, we did not assess the quality of any articles to avoid excluding data based on research design.

### Data extraction

The research team discussed the literature identified and agreed upon common themes present across the literature that were relevant to the research questions. Peer reviewed literature was loaded into the screening and data extraction software Covidence to help ensure rigor and reproducibility [19]. Each included citation was independently screened by a member of the research team (SC and VW), with conflicts and queries assessed by a third member of the research team (DR).

## Results

**Fig. 1:**
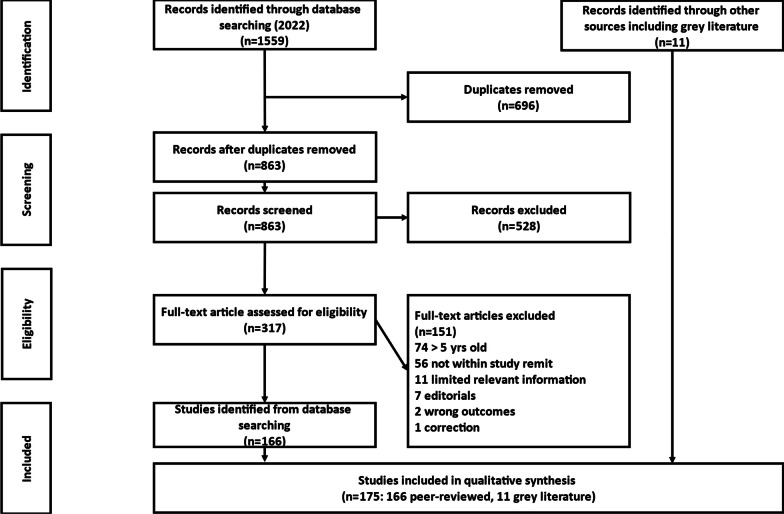
PRISMA diagram of included citations

Searches returned 1559 articles, of which 696 were duplicates. Following title and abstract screening, 319 articles were assessed at full-text stage, with 152 excluded. Excluded articles were either: outside the timeline for capture (n = 74); not within the study scope (n = 57); had limited relevant information (n = 11); editorials (n = 7); included the wrong study outcome measures (n = 2); or a correction (n = 1). Hence, a total of 166 articles met the criteria for inclusion and were included for data extraction. A PRISMA diagram relating to this process is shown in Fig. 1.

One hundred and sixty six articles were included in the review: 93 quantitative studies; 28 qualitative studies; 30 review studies (including 12 systematic reviews); 5 mixed methods studies; 8 randomised controlled trials; 1 process/program evaluations; and 1 Cochrane systematic review (see Table 1). The studies were from 20 national jurisdictions: Australia (n = 28); USA (n = 24); Canada (n = 18); Spain (n = 10); Germany (n = 7); Italy (n = 7); United Kingdom (n = 7); France (n = 5); Croatia (n = 4); Finland (n = 4); Sweden (n = 3); Norway (n = 3); New Zealand (n = 3); Switzerland (n = 2); Korea (n = 2); Poland (n = 2); China (n = 2); Portugal (n = 1); Singapore (n = 1) Cyprus (n = 1); international collaborations (n = 3). Literature reviews were not assigned a national jurisdiction.

**Table 1:** Data table for included citations

Author	Year	Aim	Method	Results/findings	Discussion
Abbott	2019	to assess recent developments in self-directed interventions for gambling disorder and at-risk gambling	rapid review of studies published between 2017–2018 regarding self-directed and primarily self-directed interventions	Limited published research little known about factors that influence likelihood of at-risk or problem gamblers engaging in self-directed change Effectiveness of self-help less as severity of gambling increases self-directed change more likely to be engaged with by high-risk or problem gamblers Self-assist change less effect size than F2F interventions and longer therapy duration combined intervention (motivational interview/workbook/follow up phone contact) demonstrated largest effect with significantly fewer relapse or remaining problem gamblers of TAU groups government and/or industry interventions shown to be varied in effectiveness self-exclusion increasingly used with moderate effect, despite low uptake, many breaches, and minimal long-term outcomes info RENO model challenged due to connotations associated with "responsible gambling" and implications for individual responsibility. Calls for adoption of Public Health/consumer protection approach based in sound understanding of risk and protective factors	At-risk and problem gamblers not likely to access self-directed/assist change programmes. Drop out, and relapse rates high, despite engagement of any kind
Armstrong et al.	2020	To test effectiveness of online intervention to strengthen contextual analytical thinking in gamblers to change gamblers cognitions and encourage safer gambling To determine whether training gamblers to think more analytically by solving a series of questions relating to common gambling diseases and providing performance-based feedback would be effective in reducing gambling-related cognitive distortions and real-world gambling consumptions among gamblers who experience gambling-related harm	participants randomly assigned to either control or intervention groups Gambling beliefs were assessed using the Gambling-related cognition scale and the Protective Gambling Beliefs Scale. Questions about gambling activity in prior week included time and money spent gambling in week prior to intervention and similar session that same week Six waves of data collection: 1 × baseline, 1 × each week (4); and 1 × post-intervention. Intervention tasks were presented following each survey and depended on condition identified during screening phase of study intervention task was an analytical training exercise int inform of an extended Gambler's Fallacy Questionnaire. Control group were asked questions related to general gambling trivia	Experimental group reported stat sig fewer erroneous cognitions, greater endorsements of protective cognitions and reduced time spent gambling post-intervention when compared with baseline. Control group also demonstrated reduced cognitions relating to predicting and controlling gambling outcomes	Cognitive interventions that encourage gamblers to challenge gambling beliefs by reflecting on gambling involvement and promoting critical thinking may be an effective tool for reducing the time spent gambling
Auer et al.	2018	To determine whether receiving personalised feedback exceeding 80% of a personally set monetary personal limit has an effect on subsequent playing behaviour of those gamblers who did not receive personalised feedback	20% of all registered NT gamblers who had set personal global spend limits and had played across one or more gambling channel. Data were matched for individuals across two time points	Matched-pairs data showed that those gamblers receiving personalised feedback in relation to limit setting showed significant reductions in amount gambled. There was no significant change in the group of gamblers in highest spend grouping	Secondary prevention—personalised feedback effective in low/medium risk gamblers
Baxter et al.	2019	To examine gambling literature to identify framework factors where research is limited, explore trends over time in selected countries and examine research foci related to gambling harms		New Zealand only country that has adopted an Public Health approach to gambling with associated gambling Act and national regulatory body Research focused on psychological and biological factors from Canada/Australia but resources and treatment received focus of NZ research—function of PH approach	Gambling research influences by jurisdictional regulation of gambling. Those countries with privately owned gambling focus on harm factors that are the responsibility of operators, whereas countries with PH focus have research identifying treatment and harm reduction resources. without legislative imperatives, research focuses on responsibilising individual with minimal attention paid to system issues and structural limitations in reducing gambling-related harm
Beckett et al.	2020	Primary aim: To investigate the perceptions of gambling venue floor staff and management in clubs in New South Wales, Australia, about barriers to staff-customer interactions, and to elicit staff-driven recommendations for improvements to current policies and procedures. To better understand their experiences and attitudes towards existing responsible gambling training programs. Study is exploratory and does not draw on established theoretical frameworks	20 employees from a Sydney sports club (convenience sample) participated in focus groups. 2 FG w gaming floor staff in customer service positions holding non-supervisory and nonmanagerial roles, 1 FG w team leaders overseeing junior staff, and 1 FG w senior duty managers. Staff volunteered to participate. Questions—the nature of each staff member’s role when interacting with patrons, perceived indicators of gambling-related harm and protocols for intervention, and attitudes towards, perceived benefits of, and improvements to responsible gambling training programs for employees. Qualitative phenomenological data were analysed using inductive thematic analyses	Three primary domains relating to staff members’ attitudes and perceptions towards current responsible gambling staff training and practices: (1) indicators of responsible and harmful gambling behaviours; (2) barriers to assisting gamblers at risk of harm; and (3) identified limitations and potential improvements to the current training. BEHAVIOURAL INDICATORS OF RG—(1) diversity of activities; (2) lack of concern about winning or losing; (3) limit setting; (4) positive emotions; and (5) socialising. These themes can be interpreted as incorporating nonfinancial motivations coupled with the objective of eliciting positive affective reactions and social interactions, consistent with a broad range of other recreational activities. BEHAVIOURAL INDICATORS OF G-RELATED HARM—(1) excessive time and money spent gambling, (2) explicit admission of harms, (3) and apathy towards personal health and physical surroundings. (4) other indicators (increasing caffeine and alcohol, playing multiple EGM simultaneously, and frequent cash withdrawals. BARRIERS TO STAFF Ix: (1) Role ambiguity around responsibilities and procedures for customer assistance, (2) fear of misidentification and reprove; and (3) feelings of inadequate legislative support. LIMITATIONS AND POTENTIAL IMPROVEMENTS TO CURRENT TRAINING: Differences in staff perceptions regarding the adequacy of current RG strategies—ranging from satisfied to more could be done to help PG. Duty managers satisfied with existing strategies while more junior floor staff felt that training and strategies were insufficient. Floor staff feedback suggested that training poorly prepares them to assist at risk-G and that current strategies do not actively reduce risky gambling behaviour. Responsible Conduct of Gambling (RCG) training informs employees about legislative and regulatory requirements, but provides little guidance for proactively approaching and interacting with patrons exhibiting indicators of gambling-related harm. Floor staff compared Responsible Service of Alcohol (RSA). RCG should provide same 'confidence and strength' to approach gamblers as they get from guidelines in the RSA	Identification needs to be followed by proactive interactions to fully determine if customer is experiencing harms that need to be addressed. Floor staff indicate that they are able to respond to patrons requesting treatment but are reluctant to take initiative in approaching patrons they perceive to be at-risk. Divergent perceptions were observed between management and floor staff in relation to customer response protocols, and this may reflect organizational culture. The trickle-down effect of management responsibility to venue operations may influence the reactive approaches taken by other staff members when responding to customers showing signs of distress. Subsequently, floor staff are uncertain about their role in assisting customers they see displaying distressing behaviours. The benefits of increased management support of proactive engagement with patrons is consistent with good corporate social responsibility
Blank et al.	2021	To identify, appraise, and synthesise existing evidence for interventions that aim to reduce gambling-related harms, and to identify gaps in the evidence base	Included only systematic review-level evidence. All forms of gambling in any setting. Participants from the whole population, identified gamblers including self-defined, and specific populations at risk. All outcome measures relating to prevention or treatment of gambling-related harms. 30 papers included in review. search end dates varied between 2011 (n = 2) and 2018 (n = 1), with half of all searches done between 2015 and 2017 (n = 16). Three papers did not state their search dates. Publication dates ranged from 2012 to 2019 (with eight reviews published in 2018–19)	Reviews were divided into those reporting on universal preventive interventions for the whole population and those evaluating selective interventions for individuals at high risk of harms. The whole-population preventive interventions included interventions to reduce the demand for gambling (demand reduction; n = 3—school-based education programs) and interventions to restrict opportunities to gamble (interventions restricting gambling activity; n = 4). Targeted interventions for individuals at increased risk of gambling-related harms included therapeutic interventions (n = 12), self-help or mutual-support interventions (n = 4), and pharmacological interventions (n = 5). We also included studies comparing different interventions (n = 2). Potential types of Ix were not represented in the SR-level evidence (1) interventions to screen, identify, and support individuals at risk of gambling-related harms (whole population) and interventions to support ongoing recovery and prevent relapse for gamblers at risk of harms. DEMAND REDUCTION: although sparse, suggests probable benefits of better gambling knowledge and attitudes of young people in the short term. However, little evidence regarding longer-term benefit exists. Establishing whether interventions are able to prevent the development of gambling-related harms in youths remains difficult. SUPPLY REDUCTION: Up-to-date review-level evidence exists, therefore, for gambling interventions that encourage individual gamblers to restrict their own gambling, with on-screen pop-up messages appearing to be the most promising approach identified, particularly high-threat messages endorsed by medical or government agencies. However, no reviews were found that considered adherence to or regulation of enforcement interventions by these agencies. Little evidence to support industry supply-reduction initiatives was found. THERAPEUTIC Ix: Considerable number of reviews but evidence only indicates positive outcomes in the ST with little evidence to support longer-term effects or to favour one therapeutic intervention or mode of delivery over another. COMPARING TARGETED TREATMENTS: Little evidence available. SELF HELP / MUTUAL SUPPORT Ix: Difficult to draw any clear conclusions at the review level because of diversity of Ix and a focus on ST self-reported behaviour change instead of LT outcomes or direct measures of harm. PHARMACOLOGICAL Ix: No conclusive support or refutation	Although studies of interventions to address so-called problem gambling or target problem gamblers can inform progress in preventing and treating gambling-related harms, it is important to acknowledge the limitations of these terms in addressing gambling-related harms at a societal or population level. Evidence from the primary literature remains sparse and weak, and review authors struggled to make conclusive statements about the evidence they examined, in terms of clear support for any specific types of intervention or for relative superiority of particular interventions or approaches over others. Recognise that mapping review not a SR
Bonnaire and Phan	2017	To examine the relationships between parental attitudes, adolescent perception of family functioning and Internet gaming disorder (IGD) and explore gender differences	PARTICIPANTS: 5 secondary schools. Final sample 434 adolescents (231 M, 203 F). 383 non-prob gamers, 37 prob gamers. MEASURES: Demographics, gaming use (including Gaming Addiction Scale for Adol), Parental attitudes to gaming, Family functioning (family cohesion, expressiveness and conflict)	GAMING: Males over-represented as gamers.PG had more screens, spent more time on weekend on Internet and more time on video games than non-PG. Gender diff in type of video game (F = management games, M = all other games). PARENTAL ATTITUDES/FAMILY FUNCTIONING: More PG played after midnight (no parental monitoring), particularly males. No differences in free and easy access to games, rules about gaming use b/w PG and non-PG. Males have more rules than females. More non-PG had been banned from gaming than PG (more males than females). Non-PG had signif. better family cohesion than PG. PG had signif. more conflict and poorer family rel than non-PG. Family cohesion and rel better in males and more conflict in females in total sample and non-PG. FACTORS ASSOCIATED WITH PG: Whole sample—time spent, banning gaming, playing late at night, playing in MMO (massively multiplayer online), and cohesion (or family relationship (depending on Model tested)	Major findings: (1) Compared to NPG, PG had significantly more screens available at home, they spent significantly more time during weeks and weekends on video games, and they played more in a MMO fashion (especially first person shooter games). (2) The time spend on video games (week and weekend), playing after midnight, and playing in a MMO fashion are positively associated with Internet Gaming Disorder (IGD). (3) Some factors associated with IGD are different in males and females. Prevalence of IGD is particularly high during adols; males over-represented. Parental monitoring is a major inhibitor of IGD in both males and females. Video games can provide relief. Aetiology of adolescent pathological symptoms of video-gaming differ by gender. This is truer for parental attitudes than about family functioning, which is similar for males and females
Botella-Guijarro et al.	2020	(1) establish the prevalence of gambling; (2) identify factors associated with gambling behaviour the following year; and (3) adjust a model to predict gambling behaviour	PARTICIPANTS: 15 high schools. Paired sample T1 to T2 (12 mths) n = 1074. 55% Female. Av age 15YO. PROCEDURE: T1 and T2 survey. MEASURES: G freq, mode, PG, Impulsivity, Sensation seeking, Self-efficacy, Peer pressure, Perceived option of G from others, Parents' att to G, norm perception, accessibility, risk perception, parent G behav, media pressure	PREVALENCE: 42.0% T2. Males more than females. Highest age of onset between 13–14 YO. Stable increase in G in adol aged 13-17YO—contrasts other studies (perhaps because diff age range). FACTORS ASSOCIATED WITH G IN FOLLOWING YEAR: Gender, gambling in T1 and risk perception were significant in 3 models with the fourth variable being sensation seeking, peer pressure (friends) and accessibility, respectively	Universal prevention should be aimed preferably at children under 15 years old and to alert regulators and public administrations to the directly proportional relationship between accessibility and gambling onset. Risk perception, sensation-seeking, peer pressure and accessibility as psychosocial variables should be considered in the design of prevention Ix
Bouguettaya et al.	2020	To establish the relationship between exposure to gambling advertising and gambling-related attitudes, intentions and behaviour	PRISMA Guidelines. Umbrella review complies evidence from multiple reviews. Encompassed years 2000–2020. Adults. Reviews must address effectiveness of interventions, actions, or policies, to prevent or reduce harm and risks of gambling. Risk or protective	24 studies included—overall lack of longitudinal, experimental. Findings similar to those found in alcohol and tobacco research. Most reliable data derived from naturalistic quasi-experimental studies related to governmental interventions to advertising (reduce, ban or increase). This data demonstrated a dose response to advertising	Lack of longitudinal data inhibits policy change and regulation. Poor quality of research increases calls of bias with implications for funding source and undue influence on research design and results
Bramley et al.	2020	To obtain insights about the accessibility of UK gambling support services from the perspectives of individuals and organisations supporting migrants experiencing gambling-related harm	PARTICIPANTS: migrants and representatives from organisations supporting migrants in Leeds and London. 2 FG w 16 participants each	Thematic analysis identified four themes: (1) the relationship between gambling-related harm and migration, (2) the ‘harm paradox’ and migrant gambling, (3) barriers to help seeking and (4) ways to improve current support. GAMBLING HARMS included financial difficulties, selling personal possessions to fund gambling, relationship breakdown, mental health problems, taking or selling illicit drugs, homelessness, domestic violence, sex work and suicide. These harms are similar to the general population; however, participants felt harms exacerbated because of lack of informal support (i.e. family and friends in UK), impacts of losses more pronounced (e.g. stigma and shame—> impact on wider community—> loss of status—> impact on health and WB, or unable to support families back home—> consequences for family). MIGRANT GAMBLING: migrants from cultures where gambling was less socially acceptable and less easy to access may gamble in the UK because it is widely promoted and visible. Disliked advertising targeting particular ethnic groups. migrants' language proficiency may affect their gambling participation and the forms it takes—may not understand risks and mechanics of G activities. Migrants, due to employment type, may try to supplement income and G environments are social and hospitable after-hours (when they finish work). Access $$ as paid in cash. Pressure to fit in—> G. BARRIERS TO HELP SEEKING: Limited awareness of G support services—types avail, how to access, costs associated. Services are inaccessible without a good understanding of English. Shame/Stigma. Fear breaches of confidentiality. Cultural preference to accept help from loved ones vs outsiders/strangers. Support service staff suggest that H/MH or disability may impact access. IMPROVEMENTS TO SERVICES: Public services to intro screening for G-related harm. G-related harm to be considered a PH issue to ensure that resources and skills were avail (voluntary or third sector orgs lacked resources and skills). PH approach may—> to staff training, targeted education, Public Health messages, interventions, and more prevention work. Inclusion of G in discussions and initiatives about health and WB, so that gambling is thought of as a lifestyle behaviour that comes with risk. Anonymised case studies within responsible gambling literature might improve the uptake of help. Accessing money advice services may overcome help seeking barriers. Raising awareness of the potential risks associated with gambling by forming partnerships between agencies and groups (culturally relevant)	Efforts should be made to ensure gambling support services are accessible to migrants and culturally sensitive
Buja et al.	2022 (data collection in 2014)	To elucidate a comprehensive risk factor models taking into account environmental, psychological, behavioural, and socio-economic variables influencing their gambling behaviour, to develop health promotion programs with a gender perspective approach	PARTICIPANTS: Adol 15–19 yrs (n = 34,922 0 53.2% Male and 34.3% Female had G at least once in past year.). METHOD: Survey—South Oaks G Screen—Revised for Adol, leisure activities, risky behave, substance abuse behave, SES, social rel, Family G, psych distress,	For M and F, G + related to leisure time spent on videogames, internet surfing, playing sports, getting into a fight, having unprotected sex, pulling stunts, drinking alcohol at least once in the previous month, having not a satisfactory relationship with teachers, receiving pocket money from parents, spending each week much money and having someone in the family (father, sister/brother, other relatives) who gambles. For M only, G + related to having poor or average school marks, going to ED in the previous year, smoking at least once in the previous month, having dissatisfied with relationships with father and having a lower family income than their friends was only associated with gambling in boys. For F only, G + related to Having an accident or injury in the previous year and having a mother who gambled. Low psych distress protective for F only	Monitoring and containing the amount of money at an adolescent’s disposal for no specific purpose should therefore be considered a valid preventive strategy for parents in both genders. goals of both preventive and therapeutic strategies should be to establish the underlying causes of stress and anxiety, and to rebuild healthy interpersonal relationships to remove the detrimental psychological substrate. Siblings’ and extended family members’ attitudes to gambling might influence adolescent gambling
Caillon et al.	2021	To evaluate the effectiveness of self-appraisal and informative pop-up messages compared to a control condition (blank pop-up messages), for both at-risk (ARG) and low risk/non-problem Internet gamblers (LR/NPG) according to their favourite type of game, in a semi naturalistic setting and with a 15-day follow-up	Participants were volunteers gambling regularly and currently on the Internet. We included ARGs [scoring 3–7 on the Problem Gambling Severity Index (PGSI) (31)] and low risk/non-problem gamblers (LR/NPG) (scoring 0–3 on the PGSI) of both sexes. Excessive gamblers (scoring 8 or higher on the PGSI) were not included because it would not have been ethical to expose them to the procedure of the study, which included a real gambling session	We observed a significant decrease in the illusion of control for ARG in the informative pop-up condition at the 15-day follow-up. A significant effect of self-appraisal pop-ups compared to blank pop-up messages was also demonstrated only for sport and horse bettors, with a decrease on time spent gambling and an increase of gambling-related expectancies at the follow-up. Finally, we also observed that a majority of the participants were disturbed and irritated by pop-ups during their gambling session	The results of our study demonstrated the limited impact of pop-up warning messages on gambling behaviour and cognition in Internet gamblers according to the type of game and the status of gamblers. The limited impact of warning messages on Caillon et al.. Internet Gambling and Pop-Up Messages gambling behaviour and the inconvenience of the pop-ups for Internet gamblers lead us to only consider warning messages as one piece of a larger responsible gambling strategy
Caillon et la	2018	To determine the effectiveness of four types of gambling moderators: limiting bonuses, self-limitation, information and self-exclusion	The participants were then randomly assigned to one sub-study (limiting bonuses, self limitation, information or self-exclusion). In the present work, only data from the “self exclusion” sub-study were analyzed (n = 60). For the sub-study “self-exclusion”, participants were randomly assigned to the experimental condition (n = 30) or the control condition (n = 30). The randomization was stratified according to their favourite game [pure chance games (n = 20), skill and chance bank games (n = 20), and skill and chance social games (n = 20)]	The results revealed that self exclusion had no short-term impact—but did have a medium-term impact—on gambling habits. After 2 months, the gambling-related cognitions (“illusion of control” and “the perceived inability to stop gambling”) and the subscale “desire” of the Gambling Craving Scale (GACS) have decreased. Participants’ opinions about the impact and effectiveness of self-exclusion were discussed	Temporary self-exclusion is an interesting tool to protect online gamblers from excessive practices, but several modifications have to be made to improve its effectiveness and use
Calado et al.	2020	Evaluate a new youth gambling prevention program based on principles of prevention and based on CBT for reducing problematic gambling behaviour, and adopting a sensation seeking targeting approach when designing preventative initiatives for your risky behaviours	Demographics of participants were collected, including gambling activity in last 12 months multiple questionnaires: Misconceptions and knowledge about gambling; attitudes towards gambling; belief sensation seeking scale RCT Case–control using pre/post test, didadic instruction to intervention groups delivered weekly during school time	111 high-school students (65 females, mean age 17.64 years) The findings demonstrated that the intervention was effective in improving correct knowledge about gambling, reducing misconceptions and attitudes, and in decreasing the total hours spent gambling per week. The intervention was also effective in reducing the number of at-risk/problem gamblers during the study period. Furthermore, these findings were stable after a 6-week follow-up	
Carra et al.	2017	To investigate whether living in certain geographic, especially deprived areas increases level of gambling, and that this will especially affect problem gambling		Severe gambling was not universally distributed across geographic locations. Males with impulsivity and drug misuse were most noted in severe gamblers. Recreational gambling was evenly distributed. Employed males in a relationship who had financial difficulties and debt were noted in the recreational group. This group demonstrated degrees of impulsivity and hazardous use of alcohol but not other drugs. Both groups smoked	In general, individual level factors explained most variance in problem/pathological gambling. Some clustering in particular areas which may explain variation in individual level factors
Choliz et al.	2021	To evaluate the efficacy of program for use in adolescents	Program implemented in 2372 adolescents (48.8% female) across 42 high schools First step: brief survey on patterns of gambling and gambling disorder were administered. Ratings scale used to evaluate frequency of both traditional forms of gambling (lotteries, slot machines etc.) and online gambling. Variables remeasured 1 month after program administered. Dependent variables: monthly gambling rates; gambling disorder; at-risk gambling Independent variable: if program was administered	Statistically significant reductions were observed in the three variables of interest: monthly frequency of gambling, percentage of adolescents with risky gambling, and percentage of adolescents with gambling disorder. The results were analysed according to sex and age (minors vs. adolescents between 18 and 19 years old)	Results obtained after applying the prevention program indicate that Ludens is effective as a universal prevention program for gambling addiction
Coste et al.	2018	To examine exclusive lottery gamblers and compare their gambling patterns and problems as well as other associated risky behaviours to those who are not exclusive lottery gamblers	Participants divided into two groups: exclusive lottery gamblers and non-exclusive lottery gamblers. Multivariate analysis	Study results reveal that ELGs, who represent two thirds of gamblers, generally exhibit less intensive gambling patterns and are less likely to report other risky behaviours. However, harms associated with moderate risk and problem gambling are found to be concentrated in specific subpopulations for both groups, primarily males, older individuals, and those who report lower income and education level	Given widespread participation in lotteries and concentration of harm within specific subgroups, these findings point to the need for prevention efforts despite the lower levels of harm associated with lottery gambling
Cronce et al.	2017	To explore associations between the independent and co-occurring use of alcohol and cannabis before/during gambling episodes and gambling outcomes		Alcohol and/or cannabis before/while gambling endorsed greater gambling quantity, frequency, negative consequences, gambling problem severity, and substance use cf those who denied using either or before/during gambling. Use of single substance (either alcohol or cannabis) associated with less severity than use of both substances. Severity associated with single substance no different for cannabis or alcohol	Use of cannabis alone before/while gambling may confer the same level of risk for negative gambling outcomes as use of both cannabis and alcohol. Prevention efforts may, therefore, benefit from targeting cannabis use in relation to gambling
Currie et al.	2020	To identify the most common self-control strategies of people who gamble regularly, the characteristics of those who use them, and assess the effectiveness of limit-setting strategies in reducing gambling-related harm		Most common control strategy was limit setting, tracking money spent, and limiting alcohol consumption. Numbers of gambling self-control strategies was positively associated with gambling involvement, annual income, problem gambling severity and playing EGMs. Approx 45% failed to adhere to limit setting, frequency, and time spent gambling. Those who stayed within limits reported less overall harm after controlling for other risk factors. Success of limit setting proportionately less effective when set at > $200 CAN/month	Findings support a Public Health approach to gambling-related harm. Longitudinal data needed to establish impact of limit setting on gambling-related harm
David et al.	2020	To explore the attitudes of young people and their parents about the alignment of gambling with sport and the strategies they perceived could assist in the prevention and reduction of gambling-related harm	Participants: Family groups consisting of 1 parent/caregiver and at least 1 child (Melbourne). Conducted 2016. METHOD: F2F interviews	PERCEPTIONS OF PROMOTIONAL STRATEGIES: Strategies clustered into themes (i) the use of imagery and appeal strategies within advertising (positive imagery, celebrities, incentives and inducements); (ii) the normalization of betting via advertising saturation and (iii) the alignment of betting with sporting codes. PROPOSED STRATEGIES FOR G HARM PREVENTION / REDUCTION: Strategies clustered around three themes: (i) regulating advertising (capping n ads, complete ban like tobacco/ alcohol—critical of gov, policy makers and industry willingness for ad regulation),; (ii) developing effective education strategies for young people (educational programs similar to tobacco and alcohol that denormalise/unglamorise sports betting, hard hitting educational campaigns that identify negative consequences) and (iii) increased acceptance for responsibility by governments and sporting organizations. Views about limited effectiveness of education: ultimately regulation of the gambling industry was needed, ‘people don’t think it is going to happen to them, until it does happen to them’, needed to be included within a range of strategies to prevent harm. Focus of education—integration of gambling education in the classroom by ‘doing things with odds in maths’, highlight ‘dangers’ associated with gambling, focus on the ‘real risks’ of gambling and emphasize the rate at which ‘you win and lose’ when placing a bet, lived experience speakers	Parents noted similarities with previously popularized products such as tobacco and alcohol. In considering responses to gambling advertising, parents often drew on the historical template provided by tobacco control. Demonstrates that community members are able to use other reference points in Public Health in considering effective responses to new and emerging Public Health issues. Awareness of financial relationship between sports betting companies and sports
Dodig Hundric et al.	2021	To examine the short-term effectiveness of this comprehensive universal youth gambling prevention program, “Who Really Wins?”, conducted by the trained program leaders, representing its first national evaluation. It also aims to contribute to a body of scientific evidence on the effectiveness of different prevention interventions in a school setting and to overcome previous shortcomings in terms of relatively small samples	n = 629 high school students from 18 towns in different regions. program implemented as part of regular prevention curricula in schools. Program leaders not obliged to conduct evaluation, therefore proportion of completed eval forms in overall participants not available. Sample of convenience and includes only students were completed the program and evaluation surveys	Results for all students showed medium positive effects on cognitive variables after the program in terms of increased gambling-related knowledge, less illusion of control, superstition, and incorrect concepts of probability related to gambling. Small or trivial effects were found on gambling behaviour in the past two months on two games of chance (sports betting and lottery), but overall, mean scores for frequency of gambling are relatively low. Results suggest that the program did not have a significant impact on students’ socioemotional skills, but mean scores for these variables were relatively high in the pre-test. The results for a subsample of boys are similar, which is to be expected given that they comprise 66% of the total sample. The results for girls did not show effects on gambling behaviour, but their gambling was not so prevalent in the pre-test. An important feature of these results is the fact that all effects are obtained in the desired direction (higher levels of correct knowledge, lower levels of cognitive distortions, lower frequency of gambling in two games of chance), proving that there are no iatrogenic effects on the measured variables	Eval suggests the universal prevention program achieves short-term effectiveness in reducing gambling-related cognitive distortions and improving gambling knowledge. However, no effects on socio-emotional skills were observed. Authors report that no effects on socio-emotional skills are consistent with earlier pilot evaluation of program and other prevention programs targeting risk behaviours. Challenging cognitive distortions and providing educational corrections have shown success in the treatment of PG. In relation to prevention, cognitive distortions have mediating role in the relationship that links probabilistic reasoning fallacy and superstitious thinking with youth problem gambling and that they can be efficiently impacted through prevention strategies. Incorrect knowledge is an important mechanism underlying risky behaviour. Alternatively, there is also evidence of dissociation between knowledge and behaviour change, suggesting that focusing on knowledge alone is insufficient in terms of prevention efforts. Effective programs combine knowledge and skill-based activities—therefore despite no ST effects on socio-emotional skills—focus should be on enhancing socio-emotional skills, especially those that have been shown to be important in preventing risky behaviour, such as problem-solving and refusal skills, critical thinking, etc. Ix effective for all students, regardless of gender (despite predominantly male gamblers). However in the long term there remains a question about the possible need to develop a gender-specific intervention. significant proportion of high school students have developed problem gambling despite their young age and that up to 20% of participants manifest some level of gambling-related consequences. Therefore, the question arises whether they are adequate participants for such a program, i.e., whether it is necessary to involve them in treatment interventions, instead. given the context in which the program is implemented, it is questionable whether a universal prevention program can achieve and sustain its positive effects in an environment where gambling is extremely available, accessible, and socially accepted
Donati et al.	2018	STUDY 1: to test a theoretically grounded model to explain gambling frequency and problem gambling referring to gambling-related cognitive distortions, i.e., a wide array of mistaken beliefs and perceptions about gambling. STUDY 2: to develop and verify the effectiveness of a preventive intervention focused on gambling-related cognitive distortions based on the model tested in Study 1	STUDY 1: PARTICIPANTS: 306 Male adol METHOD: completed measures in classroom setting (Gambler's Fallacy Task, Superstitious Thinking Scale, G-related Cognitions Scale, South Oaks Gambling Screen—revised for Adol.). STUDY 2: PARTICIPANTS: 34 male high school students. PROCEDURE/DESIGN: experimental design was conducted with two groups (Training vs. No Training) and three measurements (pre-test, post-test, and follow-up sessions). Classes were randomly assigned to the Training (16 participants) and No Training (18 participants) conditions	STUDY 1: Analyses included adol G who had G at least 1 during last yr (66% non-regular, 34% regular G). Path analysis indicated that cognitive distortions have a mediating role in the relationship that links probabilistic reasoning fallacy and superstitious thinking with problem gambling. Higher susceptibility to commit the gambler’s fallacy and higher superstitious thinking were related to greater levels of gambling related cognitive distortions. STUDY 2: 85% participants had gambled at least once during the last yr (76% non-regular, 24% regular G).. A significant reduction of the cognitive distortions from pre-test to post-test only in the Training group. FU showed stability of the training effects and the reduction of gambling frequency up to 6 mths post-Ix	There may be a cognitive-psychological mechanism through which faulty beliefs about gambling develop i.e. difficulties in dealing with probability (mindware gap) and belief in superstition and luck (contaminated mindware). Ix findings suggest that prevention strategies should address mindware problems, which can be considered as predictors of gambling-related cognitive distortions
Donati et al.	2022	To develop and evaluate a training course with the intervention providers (Study 1), and to assess the short- and long-term effects of the intervention itself (Study 2)		Results showed a significant increase of correct knowledge about gambling and a significant reduction of their susceptibility to probabilistic reasoning biases. Participants also actually learnt the main competencies to conduct the educational activities, they were satisfied for the training course received, and they felt high levels of self-efficacy The intervention was implemented with 1894 high school students (61% males; Mage = 15.68 years). In the short term, we found a significant increase of adolescents’ correct gambling knowledge, random events knowledge, and probabilistic reasoning ability, and a significant decrease of superstitious thinking, monetary positive outcome expectation, and gambling-related erroneous thoughts and fallacious behavioural choices. In the long-term, a significant decrease of gambling frequency, gambling versatility, and gambling problem severity was obtained	Overall, this work highlights the importance to train prevention program providers in order to optimize the effectiveness of large-scale gambling intervention programs towards adolescents
Donati et al.	2018	To analyse the adequacy of a model to explain gambling behaviour referring to gambling-related cognitive distortions (Study 1), and to verify the effectiveness of a preventive intervention developed on the basis of this model (Study 2)	306 male adolescents (*M*age = 17.2 years)	Mixed 2 × 2 ANOVA attested a significant Time X Group interaction, indicating a significant reduction of the cognitive distortions from pre-test to post-test only in the Training group. The follow-up attested to the stability of the training effects and the reduction of gambling frequency over time	Findings suggest that prevention strategies should address mindware problems, which can be considered as predictors of gambling-related cognitive distortions
Dowling et al.	2017	To identify early risk and protective factors (in childhood, adolescence or young adulthood) longitudinally associated with the subsequent development of gambling problems		Peer-reviewed and grey literature from 1990 to 2015 identified 15 studies published in 23 articles. Meta-analyses quantified the effect size of 13 individual risk factors (alcohol use frequency, antisocial behaviours, depression, male gender, cannabis use, illicit drug use, impulsivity, number of gambling activities, problem gambling severity, sensation seeking, tobacco use, violence, under controlled temperament), one relationship risk factor (peer antisocial behaviours), one community risk factor (poor academic performance), one individual protective factor (socio-economic status) and two relationship protective factors (parent supervision, social problems)	These findings highlight the need for global prevention efforts that reduce risk factors and screen young people with high-risk profiles. There is insufficient investigation of protective factors to adequately guide prevention initiatives. Future longitudinal research is required to identify additional risk and protective factors associated with problem gambling, particularly within the relationship, community, and societal levels of the socio-ecological model
Dowling et al.	2021	The aim of this study was to explore the potential for positive mental health characteristics (general coping, emotional support, spirituality, interpersonal skills, personal growth and autonomy, and global affect) to play a compensatory role and protective role in problem gambling in a convenience sample of 499 Australian university students	499 Australian university students (280 female, 18–56 yrs); Past-year gambling participation was reported by 75.0% of the sample, with private games (40.3%), bingo (39.0%), casino table games (32.3%), and electronic gaming machines (EGMs) (31.9%) the most commonly reported gambling activities	Hazardous alcohol use, past-year substance use, gambling-related cognitions (interpretive bias, illusion of control, predictive control, gambling-related expectancies, and perceived inability to stop gambling), gambling high-risk situations (negative and positive reinforcement situations), and gambling motives (money, positive feelings, regulate internal state, and challenge) positively predicted problem gambling severity. None of the positive mental health characteristics negatively predicted problem gambling severity, suggesting that these factors did not play a compensatory role. However, emotional support, personal growth and autonomy, and global affect buffered the influence of gambling motives and high-risk situations, suggesting that these factors played a protective role. In contrast, spirituality displayed a direct positive predictive relationship with problem gambling severity, suggesting that it served to act as a risk factor in this sample	The hypothesis that the risk factors would be positively associated with problem gambling severity was partially supported. The hypothesis that positive mental health characteristics would be negatively associated with problem gambling severity (compensatory relationships) was not supported; while the hypothesis that these characteristics would buffer the impact of a range of established risk factors for problem gambling (protective relationships) was partially supported
Dowling et al.	2021	to explore the: (1) sources of heterogeneity in the familial (paternal, maternal, and sibling) transmission of gambling problems; (2) degree to which family-of-origin characteristics are associated with family-of-origin problem gambling; and (3) beliefs of gamblers about the nature of the familial transmission of problem gambling		One-quarter (25.5%) of participants reported that at least one family member (16.5% father, 7.5% mother, 7.6% siblings) living with them when they were growing up had a gambling problem. Most participants reported that family members with a positive history of problem gambling were biological relatives, lived with them full-time, and experienced long-term difficulties with gambling. Participants with a family history of problem gambling were young (less than 12 years of age) at the onset of parental, but not sibling, problem gambling, were women, and reported difficulties with the same gambling activity as their family member. Participants raised in families with problem gambling were more likely to report parental separation (risk ratio [RR] = 2.32) and divorce (RR = 2.83), and extreme family financial hardship (RR = 1.80), as well as low levels of paternal authoritative parenting than participants raised in non-problem gambling families. Qualitatively, both social learning and genetics were perceived to play a central role in the familial transmission of gambling problems	Findings inform theories of the familial transmission of gambling problems and the design of targeted prevention and intervention strategies
Dowling et al.	2017	To examine the degree to which parenting practices (positive parenting, parental involvement, and inconsistent discipline) moderated the intergenerational transmission of paternal and maternal problem gambling	Students aged 12–18 years (N = 612) recruited from 17 Australian secondary schools completed a survey measuring parental problem gambling, problem gambling severity, and parenting practices	Participants endorsing paternal problem gambling (23.3%) were 4.3 times more likely to be classified as at-risk/ problem gamblers than their peers (5.4%). Participants endorsing maternal problem gambling (6.9%) were no more likely than their peers (4.0%) to be classified as at-risk/problem gamblers. Paternal problem gambling was a significant predictor of offspring at-risk/problem gambling after controlling for maternal problem gambling and participant demographic characteristics. The relationship between maternal problem gambling and offspring at-risk/problem gambling was buffered by parental involvement	Paternal problem gambling may be important in the development of adolescent at-risk/problem gambling behaviours and higher levels of parental involvement buffers the influence of maternal problem gambling in the development of offspring gambling problems. Further research is therefore required to identify factors that attenuate the seemingly greater risk of transmission associated with paternal gambling problems
Dowling et al.	2021	The primary aims were to examine reciprocal relationships between situational positive gambling outcome expectancies and gambling behaviour and moderators of these relationships	pre-EMA survey; PGSI; gambling motives; psychological distress; coping styles; gambling outcomes expectancies	No reciprocal relationships between EMA outcome expectancies and gambling behaviour (episodes, expenditure) were identified. Moderations predicting gambling episodes revealed: (1) cravings and problem gambling exacerbated effects of enjoyment/arousal expectancies; (2) positive emotional state and positive reframing coping exacerbated effects of self-enhancement expectancies; and (3) instrumental social support buffered effects of money expectancies. Positive outcome expectancies therefore constitute situational determinants of gambling behaviour, but only when they interact with other factors. All pre-EMA expectancies predicted problem gambling severity (OR = 1.61–3.25)	
Drawson et al.	2017	Evaluated empirical evidence on effectiveness of protective behavioural strategies (specifically individual harm reduction) in gambling	The strategies reviewed encompass four broad categories: self-exclusion (a requested ban from a gambling venue), time limit setting, monetary limit setting, and cashless, card based gambling programs	SELF-EXCLUSION PROGRAMS: Most requested by EGM PG. Effective over the period of exclusion but tend to return to gambling after exclusion period expired and PG status would increase again. Following exclusion, G freq, duration, expenditure, debt and urge reduced and maintained at 12mth FU. G who maintained enrolment reported enhanced perceived control, a stronger belief in the program's effectiveness, and that gambling was disrupting their life less. Self reported benefits of self-exclusion include improved psychosocial functioning, reductions in emotional strain and interpersonal and familial difficulties, increase in work performance, gambling-related QoL and self-confidence. Breach of self exclusions is a concern, due to lack of enforcement. TIME LIMITS: Limiters gambled for less time compared to those without limit and many would gamble for less time than their own limit. High frequencies of strategy use but inconsistent evidence for this. PG more likely than non-PG to set time limit but also exceed it. Pop-up messages appear to add time limiting. Daily and session duration limits resulted in decreases in play time at 30 days FU. MONETARY LIMITS: Highly endorsed strategy. of both PG and non-PG. Inconsistent evidence. Setting limit prior to or early in play provides more protection. Other strategies include set amount of case taken to gamble with, leaving cards at home. Reduce bets per wager. PG tend to track spend more than non-PG. PG exceed self-imposed loss limits. CASHLESS CARD-BASED EGM G PROGRAMS: Very few gamblers report often or always using cashless card. Variations in reports of potential benefits. OTHER STRATEGIES—demonstration play, G self-management techniques, Player information display system, gambling with friends/family, use of a behavioural feedback tool with a traffic light metaphor, referring to a clock /watch. The amount of evidence available was limited for the majority of strategies; the evidence for self-exclusion was the most promising, but results were inconsistent across studies. Quality of evidence was low	Self-regulation is required for time and monetary limits. PG set greater higher limits than non-PG. Games do not result in shut out if limits reached. So protections are limited and rely on individual to stop G. The literature suggests that many individuals are using PBSs when they gamble. However, information regarding who is using them and for whom they are effective is largely unknown, despite the fact that responsible gambling initiatives commonly promote PBSs. Alcohol research shows PBSs are less effective for those who drink to reduce negative emotions; same may hold for gambling. Problem gamblers were significantly more likely than non-problem gamblers to utilize most strategies. This pattern is consistent with recent literature on the use of PBSs in alcohol
Drummond et al.	2020	To investigate relationship between spending on loot boxes in video games and symptoms of problematic gambling	A sample of 1,049 participants were recruited through Qualtrics’ Survey Targeting service from a broad cross-section of the population in Australia (n = 339), Aotearoa New Zealand (n = 323), and the United States (n = 387). Participants answered a survey assessing problem gambling, problem gaming symptomology, and how much they spent on loot boxes per month	On average, individuals with problem gambling issues spent approximately $13 USD per month more on loot boxes than those with no such symptoms. Loot box spending was also associated with both positive and negative moods, albeit with small effect sizes. Analyses showed both interactions and correlations between problematic gambling and problematic gaming symptoms, indicating both some commonality in the mechanisms underlying, and independent contributions made by, these proposed diagnostic criteria	
Dussault et al.	2019	The goal of this study was to (1) identify groups with various patterns of poker playing among adolescents, based on different modalities and forms, and (2) compare the identified groups according to various characteristics, from sociodemographic to gambling-related	The sample was constituted of 759 adolescents (70.8% boys; M age 15.44 years, range 14 –19) recruited in high schools and who had played poker in the last year. The statistical fit indices revealed a four-class solution. Class 1 almost exclusively played simulated poker. Class 2 played poker exclusively in the school context. Class 3 played poker almost exclusively at home. Class 4 showed a very diversified pattern regarding their modalities of poker playing	The statistical fit indices revealed a four-class solution. Class 1 almost exclusively played simulated poker. Class 2 played poker exclusively in the school context. Class 3 played poker almost exclusively at home. Class 4 showed a very diversified pattern regarding their modalities of poker playing Results of the logistic regression suggested that gambling-related variables (e.g., time spent playing, reading about gambling strategies and diversity of gambling funding) were significant predictors of class membership. This study shows that there is a variety of profiles among young poker players. Although one profile has few risk factors (simulated poker gamblers), others have more factors associated with adults’ gambling problems	These profiles suggest that specific prevention strategies are probably appropriate to reach these different groups of young people
Dussault et al.	2021	To examine the effect of gambling location on frequency, expenditure, and time spent on cash game poker in relation to individual characteristics of gamblers	Data were drawn from a 2012 Québec epidemiological gambling survey. The quantitative analysis used multilevel methods to model the dual-level hierarchical design of gambling location (level 1) and individual characteristics nested within poker cash game players (level 2). The sample was comprised of 270 individuals aged 18 years and above and living in private homes, who reported gambling on poker cash games in the past 12 months. Participants reported their gambling habits in up to three locations: private homes, the casino, and the Internet	The most frequently reported gambling locations were private dwellings (87.4%), followed by casinos (15.9%), and the Internet (13.7%). Some interactions between location and the demographic variables were observed. Moreover, the multilevel analysis revealed an important relationship between the location and poker cash gambling behaviour	Study reveals the significance of contextual factors as a fundamental element in gambling behaviours and highlights the need for prevention strategies that target specific high-risk contexts rather than individually based interventions
Elbers et al.	2020	Description of collaborative approach between statutory (financial, licencing and PH) and voluntary services and experts by experience to address problem gambling within Leeds City Council area	1. Gathering data by commissioning local research—gain a more comprehensive understanding of G-related harms in Leeds. 2. Disseminating the evidence by holding a national conference in Leeds—encouraged Council's directorates and partners to work together to develop a coordinated approach. 3. Harnessing stakeholder engagement and building capacity by setting up PG Project Grp—representatives from Council including Public Health, welfare advice partners, national commissioners and treatment providers, local gambling operators and the specialist mental healthcare provider. Aim was to raise awareness of how to recognise, signpost and support those suffering from or at risk of gambling-related harm. 4. Public engagement and raising awareness via Beat the Odds marketing campaign—see Ix Column. 5. Training and briefing sessions delivered to frontline staff—See Ix Column. 6. Enhancing the narrative by gathering local stories and deeper insights—contacted GA branch to access "lived" experience, Interviews to explore Uni students attitudes to G. FG to explore how G affected migrants. Stakeholder scoping group RE g-related suicide. 7. Increasing Tx and support by developing a new G hub and G service—HUB: satellites bases, treats people with the most serious and complex needs, clinical team of Psych, MH nurses and others, research element to evaluation Ix and develop future Tx models. SERVICE: prevention and HR (identifying, screening and treating PG and AO). Outreach and training, brief Ix, engagement with primary care / Criminal Justice, counselling, onward referrals for specialist Tx. Co-located with Council. 8. Building a regional approach	CAMPAIGN: During the combined campaign periods, average visits to the Leeds Money Information Centre webpage have increased by over 180%. As a result of the first campaign in 2017, the regional BBC ran a programme on problem gambling featuring the Beat the Odds campaign. TRAINING: Subsequently, other local authorities in the region were demonstrating increased interest and demand for locally delivered training. This, alongside a positive evaluation of the training, contributed to GambleAware's announcement in November 2018 for a CAB in each region to start the provision of training. NARRATIVES: GA members became involved in the development of the expanded treatment and support services. Qual study recommended challenging normalising of gambling behaviour amongst young adults and a greater focus on recognising potential harms for this cohort. TX SERVICES: will act as a pathfinder, from which a model of care can be rolled out across the country	Council has used its resources through Council staff capacity and the Social Inclusion Fund to improve research evidence, training and stakeholder engagement, treatment and support and public awareness. This has extended throughout the region and other councils through working with national bodies. A creative, pragmatic and co-produced approach
Elbers et al.	2018	To examine how three factors mediate the relationship between marital status and problem gambling: (1) gambling because of loneliness; (2) gambling to socialize; and (3) social context	This study was designed to better understand the role of gambling in the lives of older adults and the prevalence of gambling and problem gambling among this group. We defined older adults as those individuals who are age 55 years and older. Participants were randomly selected to participate in an onsite, intercept survey in non-gaming areas of the gambling venues (entering/exiting). Respondents were selected by gender and age (55–64, 65–74, 75 and older) Measures included: PGSI; social motivations for gambling; social context of gambling; marital status; socioeconomic co-variates	66.7% married; 38.8% gambled to socialise; gambling with others motivation for 13.5%; 74.5% gambling socially (came to casino with family or friends) PGSI highest in those who gambled with others due to loneliness. Those who gambled to socialise had lower PGSI than those who did not gamble to socialise. those who gambled alone had higher PGSIs than those who travelled to casino with others Problem gambling highest in divorced respondents followed by single respondent. Married respondents had lowest PGSI Highest PGSI seen in divorced women	Prevention and treatment initiatives should examine ways to decrease loneliness and social isolation among older adults and offer alternative social activities
Engebo et al.	2019	Study investigates the beliefs about RG measures and if beliefs could be explained by risk factors such as demography, gambling behaviour, personality traits and self-reported impact from gambling advertisement	The present study is based on a general gambler population and investigates how RG measures with some specific RG features are assessed by the gamblers. The data was collected in 2013 and 2015. The samples were drawn from the Norwegian Population Registry. In total 9129 gamblers participated. Gamblers were asked to state to which degree they agreed that ten specific RG measures help or would help them controlling their gambling	A multiple regression analysis identified eleven variables as significant predictors of positive beliefs about RG measures: female gender, young age, playing random games only, being a moderate risk or problem gambler, reporting high impact from gambling advertisements as well as the personality traits agreeableness, openness and neuroticism. Playing low risk games only, reporting a high amount of spending on gambling and the personality trait extraversion were inversely related to positive beliefs about RG measures	Positive beliefs about RG measures can relate to needs for external based countermeasures to minimize or reduce problems. Negative views may reflect a wish to play without obstacles, take risks or to trust in self-control
Esteves et al.	2021	The aims of the present longitudinal study are (1) to evaluate the change produced after one year in gambling disorder, gambling-related cognitive bias, compulsive buying, and materialistic values and (2) to examine the gender role in these changes and to analyse the mediational mechanisms among the variables of the study		Results show significant decreases in compulsive buying, materialism, and cognitive biases related to gambling after one year. Gambling disorder severity was directly related to cognitive distortions of gambling and being a man. Compulsive buying was associated with older age and the female gender. Materialism was associated with compulsive buying and the male gender	The understanding of gambling disorder and compulsive buying in adolescents could potentially lead to early prevention and treatment programs for the specific needs of gender and age
Farhat et al.	2022	To examine casino gambling in relation to at-risk/problem gambling with respect to sociodemographic characteristics, gambling perceptions and attitudes, health/functioning measures and gambling behaviours		Approximately 11% of adolescents acknowledged gambling in casinos. ARPG was more frequent and gambling perceptions were more permissive among adolescents endorsing casino gambling. Stronger relationships between ARPG and heavy alcohol and drug use and weaker relationships between ARPG and engagement in extracurricular activities, gambling with friends, gambling with strangers and gambling for financial reasons were observed among adolescents endorsing casino gambling	Gambling in casinos was endorsed by a sizable minority of adolescents who gamble, and prevention efforts should consider targeting permissive attitudes towards gambling, adolescent drinking and participation in extracurricular activities when addressing underage casino gambling
Flack & Morris	2017	The intention to gamble, gambling frequency and gambling problems can be explained by the constructs embedded in the Theory of Planned Behaviour (TPB). Therefore (1) to examine whether gambling problems are mediated by frequency or if there are direct associations between the TPB determinants and gambling problems and (2) to examine if cognitive biases, independently, explain gambling problems or if they form part of a wider set of beliefs. The current study uses the TPB as a framework to investigate these relationships	PARTICIPANTS: 201 uni students (124 female, 75 Male, 2 Gender not specified). MEASURES: Constructs measured: Attitudes towards G, (developed for study, Perceived Social Norms, Perceived Control, G intentions, G probs, G freq, PROCEDURE: Survey	Using the TPB as a framework, perceptions about the anticipated outcome of gambling, the influence of others, and beliefs in skill and knowledge were simultaneously modelled to assess their effects on gambling problems via intentions and past year gambling frequency. Path analysis revealed TPB determinants predicted gambling frequency (56% of variance) and gambling problems (55% of variance), respectively. Interestingly though, there was the direct path between the intention to gamble and gambling problems, and attitudes and gambling frequency. However, counter to expectations, anticipated outcomes (attitude) did not independently relate to gambling intentions	Consistent with the TPB, social norms and cognitive biases predicted intention, which, in turn, predicted gambling frequency. Likewise, gambling frequency correlated with gambling problems
Forsstrom et al.	2022	1. to explore the level of alcohol use and gambling problems in a sample of Swedish university students Subgoal: to describe the prevalence of different levels of drinking and/or gambling associated with risk and harm and to compare impulsivity and sex differences between students who have no self-identifies drinking and gambling problems and students that have self-identified alcohol and / or gambling problems associates with risk Additional aim: test whether age acts as mediator for impulsivity and negative consequences from alcohol use and gambling	Participants recruited via Facebook and university SM pages Descriptive stats; Chi Square; mediator analysis on impulsivity measure and total AUDIT score	794 participants (532 female); mean age 28.9 yrs Alcohol problems higher in participant group than Swedish population but risk of gambling lower than general population. Level of medium risk for gambling lower in sample than general population but potentially skewed by gender higher rates of impulsivity and male gender positively associated with higher risks of alcohol use and gambling age only partial moderator and sex not a moderator for impulsivity—again potential skewing by aged and gender of sample	targeting of young males may offset future difficulties. Specific messaging around alcohol and gambling may be effective
Frisone et al.	2020	To highlight the implications of adolescent gambling, taking into consideration the main risk and protective factors aimed at limiting gambling activity as well as the main links with the impusle-control disorder, addiction, and behavioural addiction	The review process was conducted through Medline, Scopus, Web of Science and Google Scholar search engines. The considered keywords were “Gambling” AND “Adolescence,” “Behavioral addiction,” AND “Adolescence.” The articles related to gambling and adolescence in the last 20 years were included in compliance with inclusion and exclusion criteria, to perform a consistent analysis of the phenomenon and the related maintenance factors	107 articles extracted Main themes: gender differences: pathological gambling generally seen in adolescent males. Female adolescents more likely to engage in casual gambling. Substance use associated with gambling attitudes Drug use: some evidence of high probability of development of both gambling and substance use disorder in adolescents. Risk and protective factors associated with smoking and drugs similar for gambling technology: Increased access to online gambling for adolescents increases risk of psychopathological habits. Technology contributes to development of psychopathology linked to addictive behaviours and goes beyond protective factor like parental control Age differences: Adolescents at greater risk of poor affective decision making cf children. Enhanced emotional intelligence considered protective factor Impulsivity and sensation seeking: Some evidence that childhood impulsivity associated with higher probability of developing gambling issues as adolescent Societal factors must be taken into account when assessing risk and protective factors	Age and gender differences contribute to addictive behaviours, and use of psychoactive drugs positively associated with behavioural addictive behaviours
Gainsbury et al.	2020	To understand the extent to which consumer protection tools are used, characteristics of those using these tools, and the perceptions and attitudes towards tool use, including barriers to use	Online survey of 564 customers of Australian Internet gambling sites	Most participants were aware of the tools and had accessed activity statements; few had used deposit limits (24.5%) or time-outs (8.1%) but use of these restrictive tools was higher among those at risk of gambling problems. Satisfaction with tools was generally high among users and tools were mostly used as intended; however, only moderate changes in behaviour were reported. Participants predominately did not use the restrictive tools as they did not see these as relevant for them, and they were perceived to be intended for people with gambling problems	Findings are important to drive necessary improvements to consumer protection efforts including efforts to encourage perception that tools are relevant for all customers. Changes to current practice, including terminology and promotion of tools, are needed by Internet gambling operators and policy makers to improve the utilisation and effectiveness of consumer protection tools to enable sustainable gambling among the broader cohort of Internet gamblers
Gainsbury et al.	2018	To understand hypothesised differences between cohorts of gamblers and receive qualitative feedback on archetypal targeted messages used to increase responsible gambling tools		Cohorts exhibited different preferences and responses to message archetypes. Seniors preferred messages about limit setting, whilst young adults and frequent gamblers responded to messages about their own play and expertise. Skill game gamblers were interested in the odds of winning and their own outcomes over time. However, all groups agreed that using positive, non-judgmental language in messaging is important	
Giralt et al.	2018	To provide prevalence data and clinical description of factors related to problematic gambling among adolescents in two different German states	Representative samples of students aged from 12 to 18 years (N = 9,309) in two German federal states to provide prevalence data and clinical description of risk factors for problematic gambling	About 40% of the adolescents reported engaging in gambling activities within the past 12 months and found prevalence rates of 1.7% and 2.2% for problematic gambling. Especially, use of online gambling and slot machines was found to be related to problematic gambling. Male adolescents with a migration background were of higher risk for problematic gambling and psychopathological symptoms were significantly elevated among that group	Results indicate that participation in gambling activities is common among underaged adolescents and that prevalence of problematic gambling exceeds rates of adults. Similarly, problematic gambling is associated with increased psychopathological strain
Gomez et al.	2020	Aim of this study, carried out in the Galician region of Spain with a sample of 3772 students aged between 12 and 17 years, was to estimate the prevalence of online gambling in minors; to characterize the profile of online gamblers; to explore the differences in Internet and smartphone usage habits, online risky practices, problematic Internet use and parental involvement between online gamblers and non-online gamblers; and to analyse the relation between online gambling and academic performance		Results revealed that 6.5% of Galician adolescents are online gamblers, a figure that has more than tripled over seven years. 90% of online gamblers are male, and their mean age is 15. Online gamblers had significantly higher rates of problematic Internet use, active sexting, cyberbullying, or contacting strangers through the Internet. Furthermore, online gamblers had higher scores on impulsiveness, lower scores on assertiveness, and were lacking parental control	Data show that online gambling is not an isolated problem, so prevention should be understood in a comprehensive manner
Gonzalez-Roz et al.	2017	Main goals of this study were to (1) estimate gambling prevalence among adolescents both in terms of different levels of severity, and in terms of modes of access and (2) to explore possible predictors of gambling severity		1312 adolescents 14—18 yrs Measures: demographic; gambling behaviour and related characteristics; gambling-related problems Analysis: descriptive stats Higher proportion of problem gamblers did not live with parents and had relatives with gambling problems	The prevalence of at-risk and problem gambling was 4 and 1.2%, respectively. Regression analyses showed that having a relative with gambling problems predicted at-risk gambling. Both living with only one parent or not living with parents at all, and the prevalence of Electronic Gambling Machines in the last year were associated with problem gambling. Mixed-mode gambling was a predictor of both at-risk and problem gambling
Granato et al.	2018	The goal of this study was to examine the associations between three subscales of the Protective Behavioural Strategies Scale (PBSS) and gambling consequences in a college sample of gamblers who also met criteria for alcohol or drug abuse. We hypothesized that engaging in more drinking PBS would be associated with lower levels of gambling consequences	Sub-sample (312 students) of larger study on gambling behaviours among college students Measures: demographics; measures of gambling, alcohol or drug use; related consequences, but no drinking Protective behavioural strategies	A sample of 316 students (55% female) completed an online survey and met criteria for problematic gambling behaviour (3 or more on the South Oaks Gambling Screen and 1 or more consequences on the Gambling Problem Index). Those endorsing a higher score on the Serious Harm Reduction subscale (but not the Stopping or Limiting Drinking or Manner of Drinking subscales) showed a lower level of lifetime gambling consequences, suggesting a crossover effect	
Grande-Gosende et al.	2020	To summarize the existing literature on the effectiveness of prevention programs aimed at reducing the prevalence of gambling problems in young adults	A systematic and comprehensive search was conducted in June 2018. The authors searched six electronic academic databases (PubMed, ISI Web of Science, Scopus, ScienceDirect, PsycINFO and Cochrane) to identify potentially eligible studies, by combining the following terms: “prevention”, “gambling”, “program”, “youth”, “young adults”, “young people” and “college students”, as well as all their derivatives. A data filter was applied in order to select published papers from the last 20 years (1998–2018)	Three key findings were found. First, all the analysed studies included prevention strategies targeting young adults enrolled in college settings. Second, such gambling prevention programs mostly followed a selective or indicated prevention strategy under the scope of a harm-reduction model. And third, the existing literature revealed that the Personalised Normative Feedback approach is the preferred strategy for reducing at-risk or problem gambling among young adults, showing at least a moderate positive effect in most of the included studies	The limited number of studies included in this review highlights the need to address scientific quality standards before proceeding with the design, implementation and widespread dissemination of these preventive programs as well as the need to ensure the program’s efficacy prior to implementation
Gupta et al.	2021	This study provides (1) lived experience of individuals who reported experiencing harms from gambling, (2) insights into help-seeking for gambling issues, (3) and people’s views on current legislation on gambling in the NT	Semi-structured interviews were conducted with a targeted selection of respondents from the 2015 and 2018 NT Gambling Prevalence and Wellbeing Surveys. The sample (n = 27; age 18 + years; Aboriginal) included weekly (online and venue-based electronic gambling machine (EGM)) gamblers, nonregular gamblers, and those negatively affected by others’ gambling. A Framework Analysis approach was used for data analysis	Negative impacts and harms from gambling were experienced by both gamblers and non-gamblers. These included monetary losses, relationship conflicts, emotional distress, and decrements to health. A lack of self-realisation of gambling issues and awareness of the available services, shame, and embarrassment, were reported as the main barriers to help-seeking. Where help was sought, it was primarily informal (e.g., family) and was rarely preventive. In many instances, self-help strategies were successful in controlling one’s own gambling. Gamblers suggested regulations should set limits on the daily number of hours of playing, the bet size, and reduced access to EGM. The need for strengthening the existing awareness and education interventions was emphasised	Viewing the findings from a Public Health lens, targeted approaches based on specific circumstances may have the potential to minimise harms from gambling, but only for those already experiencing harms. The treatment, policy, and regulatory approaches need to be tailored to address the causes and impacts of harms experienced by people
Harris et al.	2017	study reviews the theoretical argument underpinning the application of specific harm-minimisation tools, as well as providing one of the first critical reviews of the empirical research assessing their efficacy, in terms of influencing gambling cognitions and behaviour		Tool categories: **Forced breaks in play**: minimally effective and potentially harmful in absence of supporting mechanisms like self-appraisal messaging **Messaging:** Static messaging vs dynamic messaging: dynamic messaging that temporarily interrupts play more effective than static messages on side of machine screen Informative messaging: some efficacy in messages that corrected erroneous cognitions, misconceptions or probability and likelihood of winning **Self-appraisal messaging:** messages that directly encourage player self-appraise time and money spent more effective than messaging around likelihood of winning and RTP odds **Monetary and time-based messaging:** may combat potential for dissociation by players. Some evidence that players will stick to pre-set limits if pop-ups are money focused **Normative feedback and enhanced messaging:** Personalised Normative Feedback (PNF) corrects perception of normal levels of engagement in specific behaviours. PNF clinically effective when combined with motivational interviewing **Limit setting:** Some effectiveness in reducing spend amounts but not maintained at 30 day follow-up. Most effective intervention pop-up monetary reminders AND limit setting **Behavioural tracking tools:** Used in conjunction with PNF models and in line with Stages of Change Model **Prohibition and Modification of note acceptors:** little evidence of efficacy of prohibition/modification of note acceptors on total spend in EGM players	Results appear to support the notion that harm-minimisation tools should be viewed as a responsible gambling prevention measure for those who already gamble safely, or are at risk of developing a problem, rather than an intervention for those already exhibiting problem gambling behaviour
Heiskanen and Matilainen	2020	To discuss and analyse the gambling habits and perceptions towards gambling cultures and problems among the large ‘baby boomer’ generation in Finland from an intersectional approach		The main results concern the gambling habits on the participants’ life course: from shared, cultural experiences in their childhood to mostly minor gambling on the edge of retirement; the mutual understanding of the enormous growth in gambling supply during their lifetime, but emphasizing the importance of gambling monopoly for the society; and framing gambling problems as an individual tendency	The prevention of gambling harm within this generation needs to take into account the historical changes they have lived: from few, harmless gambling products framed as an instrument to support ‘good causes’ to the current world of commercial gambling. The deterministic understanding of gambling problems as an individual flaw may prevent recognizing problem gambling and seeking help to tackle the problems. Risks for gambling harm relate more to the gambling structures and cultures this ageing generation lives in, and the deterministic, individual understanding of gambling harm they share, than to marginalized positions they may have through gender or education
Hing et al.	2017	This study aimed to (1) determine how use of RG-related strategies differs amongst regular gamblers by gambler risk group; and (2) identify RG related strategies whose usage predicts non-problem/low risk gambling	Regular Australian gamblers on high-risk products (EGMs and online sports betting) (N = 860), recruited through gambling venues and an online wagering operator, were surveyed about their use of RG strategies promoted on the website of their jurisdiction’s main RG agency	Knowledge of RG strategies was reasonably high amongst all gambler risk groups, but lower-risk groups were more likely to use RG strategies. A logistic regression correctly predicted 82.1% of lower-risk gamblers and 77.2% of higher-risk gamblers. Predictors of lower-risk gambling included: greater confidence in their understanding of RG; endorsement of lower gambling expenditure and frequency limits; fewer erroneous gambling beliefs; being less likely to gamble to win money, challenge their skills/beat the odds, or forget about worries and stresses; and being more likely to gamble for pleasure/entertainment. Lower-risk gamblers were more likely to set a money limit in advance of gambling and to balance their gambling with other activities	
Hing et al.	2019	This study aimed to: (1) identify a parsimonious set of evidence-based Safe Gambling Practices (SGPs) that best predict nonharmful gambling amongst gamblers who are otherwise most susceptible to experiencing gambling harm; (2) examine how widely are they used; and (3) assess whether their use differs by gambler characteristics	A sample of 1,174 regular gamblers in Alberta Canada completed an online survey measuring uptake of 43 potential SGPs, gambling harms and numerous risk factors for harmful gambling	Elastic net regression identified a sub-sample of 577 gamblers most susceptible to gambling harm and therefore most likely to benefit from the uptake of SGPs. A second elastic net predicted gambling harm scores in the sub-sample, using the SGPs as candidate predictors Nine SGPs best predicted non-harmful gambling amongst this sub-sample. The behaviour most strongly associated with increased harm was using credit to gamble. The behaviour most strongly associated with reduced harm was ‘If I’m not having fun gambling, I stop’	These SGPs form the basis of evidence-based safe gambling guidelines which can be: (1) promoted to consumers, (2) form the basis of self-assessment tests, (3) used to measure safe gambling at a population level, and (4) inform supportive changes to policy and practice. The guidelines advise gamblers to: stop if they are not having fun, keep a household budget, keep a dedicated gambling budget, have a fixed amount they can spend, engage in other leisure activities, avoid gambling when upset or depressed, not use credit for gambling, avoid gambling to make money, and not think that strategies can help you win
Huggett et al.	2020	The primary goal was to test our hypotheses that: (1) alcohol use causes increases in gambling behaviors/frequency and (2) common genetic/environmental risk factors explain the association between alcohol and gambling behaviors	Our full sample included 2,860 individuals participating in the Colorado Center on Antisocial Drug Dependence (CADD; PI: J.K. Hewitt), who were of legal drinking and gambling age (age at assessment > 21). The full sample included sibling pairs who grew-up together and were ascertained through either clinical or community settings. Participants were excluded if they had an IQ < (80 – standard error), had a history of psychosis or were not English speakers	Controlling for all covariates and shared genetic/environmental factors, we found increased alcohol use directly predicted more frequent gambling behaviors (consistent with causality). Our study also suggests shared genetic and/or environmental risk factors contribute to the association between increased alcohol use and frequent gambling behavior, a finding that may be more pronounced in males	Findings support both common risk factors between alcohol use and gambling as well as a direct relationship between alcohol use and gambling frequency. Recognizing these dual processes could prove useful for gambling-related prevention/intervention programs
Huic et al.	2017	To present an original Croatian youth gambling prevention program that was developed in accordance with the theoretical principles of prevention science and already established effective youth gambling prevention efforts. Our aim was to test short-term effects of this program designed to target both risk and protective factors associated with adolescent gambling and general risk behavior	Experimental design using two groups (training and non-training) with pre-test and post-test measurement. Training comprised didactic session for 14—16 yr olds focused on misconceptions related to sports betting; interconnectedness of our behaviours; problem solving skills; importance of peers in adolescent life and resistance to peer pressure	Results showed significant changes in the post-test sessions, which can be attributed to changes in the Training group. We observed a decrease in risk factors, namely better knowledge about gambling and less gambling-related cognitive distortions. Immediate effects on protective factors such as problem solving skills, refusal skills, and general self efficacy were not observed. Findings also show program effects to be the same for both boys and girls, students from different types of schools, for those with different learning aptitudes, as well as for those at different risk levels with regard to their gambling, which speaks in favour of the program’s universality	The program had no iatrogenic effects on behaviour change and shows promise as an effective tool for youth gambling prevention. Future research and a long-term evaluation are needed to determine whether the observed changes are also linked to behavioural change
Huic et al.	2017		Canadian Adolescent Gambling A total of 1,372 high-school girls from 7 Croatian cities participated in the study. They provided data on their gambling activities, peer gambling, cognitive distortions related to gambling, motivation for gambling, and levels of general risky behavior. As the only instrument developed specifically for use on adolescents, the Canadian Adolescent Gambling Inventory was used to examine adverse gambling consequences	7.4% of girls can be considered regular gamblers, and out of those who gambled at least once in their lifetime (n = 862), 11.2% already experience mild adverse consequences because of their gambling (at risk gamblers), with 3.2% experiencing serious consequences (problem gamblers). In general, girls seem to prefer lotto and scratch cards, but sports betting seems to be the preferred game of choice among regular girl gamblers. A hierarchical regression model confirmed the importance of much the same factors identified as risky for the development of problem gambling among adolescent boys—cognitive distortions, motives to earn money, to be better at gambling and to relax, the experiences of winning large and the drive to continue gambling, together with social factors such as having friends who also gamble, being involved in other risky and delinquent behavior and higher gambling frequency	Findings indicate that prevention efforts need to be aimed at girls just as much as at boys. Prevention strategies should use some gender specifics but information around potential harm should be universal
Jacob et al.	2022	The aim of this study was to investigate the association between problem gambling and functional disability in a UK nationally representative sample	Cross-sectional data from the 2007 Adult Psychiatric Morbidity Survey were analyzed. Problem gambling was assessed using a questionnaire including 10 DSM-IV criteria, whereas functional disability referred to at least 1 difficulty in 1 of 7 activities of daily living and instrumental activities of daily living. Control variables included sociodemographic factors, smoking status, alcohol dependence, drug use, the number of chronic physical conditions, depression, and anxiety disorder. The problem gambling-functional disability relationship was studied using a logistic regression model	This study included 6941 adults aged ≥ 16 years (51.2% women; mean [SD] age 46.3 [18.6] years). The prevalence of functional disability was significantly higher in the at-risk problem gambling/problem gambling group than in the no problem gambling group (46.2% vs 32.1%, *P* value < 0.001). After adjusting for control variables, both at-risk problem gambling (OR = 1.55, 95% CI = 1.03–2.35) and problem gambling (OR = 3.05, 95% CI = 1.09–8.52) were positively and significantly associated with functional disability	In this large representative sample of UK adults, problem gambling was associated with higher odds for functional disability. If confirmed with longitudinal studies, these results suggest that those suffering from problem gambling should receive targeted intervention to aid in the prevention of functional disability
Jauregui et al.	2020	The aims of this study were, first, to analyze gender differences in gambling motives and gambling severity; second, to analyze the relationship between gambling motives and attachment, coping, emotion regulation, and gambling severity in a community sample of adolescents and young people; and third, to analyze the mediating role of attachment, coping, and emotion regulation among gambling motives and gambling severity	472 students (222 females) recruited from high schools and vocational education centers (mean age 15.6, SD = 1.33). Gambling motives, gambling severity, parent and peer attachment, coping strategies, and difficulties in emotion regulation were assessed	The results showed that parent and peer attachment correlated with gambling motives (enhancement, social, and coping), whereas parent attachment predicted gambling motives. Difficulties of emotion regulation correlated with gambling motives, with lack of control standing out as the most significant predictor. Coping strategies also correlated with gambling motives, and maladaptive strategies predicted gambling motives. Additionally, gambling motives correlated with gambling severity, with coping and enhancement motives as predictors of gambling severity. Moreover, boys reported more enhancement motives and gambling severity than girls. Finally, difficulties in emotion regulation mediated the relationship between gambling motives and gambling severity	These results may be useful for prevention and intervention in gambling disorder in adolescents and young people
Jonas et al.	2020	This study investigated the efficacy of two interventions for problem gamblers provided online by the German Federal Center for Health Education (BZgA). The first intervention is the guided program “Check Out” (CO), the second is email counselling (EC)	A web-based randomized controlled trial with follow-up surveys after 3, 6 and 12 months was conducted. Participants were allocated to CO, to EC or to a waitlist (WL). Outcomes were the degree of problem gambling according to the Problem Gambling Severity Index, the number of days gambled in past 30 days, the highest stake during the past 30 days and the subjective well-being (WHO5)	167 individuals were included in the trial. In comparison to the WL at the 3 months follow-up, participants of CO showed significant improvements with moderate to strong effect sizes in all outcomes. Strongest effects were found in the problem gambling severity (d = 0.91; p = 0.023), followed by the well-being (d = 0.70; p = 0.011), the gambling days (d = 0.59; p = 0.001) and the highest stake (d = 0.55; p = 0.012). Improvements were sustained until last follow-up. Compared to the WL, users of EC had beneficiary results in the problem gambling severity (d = 0.74; p = 0.022). No significant effect differences were found between CO and EC. However, according to process evaluation, users of CO reported a significantly stronger working alliance than users of EC (d = 0.70; p = 0.019) and used the intervention considerably longer (d = 0.84; p = 0.004). CO helps treatment-seeking individuals to sustainably reduce their gambling behavior and to increase their general well-being. Compared to EC, CO seems a better support option, since its effects include a wider range of outcomes	Possible reasons are the more engaging program structure and elements of CO, as well as the closer interaction between client and counsellor
Jones et al.	2021	To examine the influence of two potential risk factors on sports betting behaviour and problems: erroneous beliefs and athletes' emotional involvement	201 athletes with different levels of expertise completed a newly developed scale to assess both factors. s. Participants were sampled from the general German population, predominantly male (83.08%) and on average 29.52 (SD 5 11.05) years old. We use principal components analysis to detect patterns of covariation, potentially due to the proposed underlying latent factors, and regression analyses to test associations of these factors with betting behavior and problems	Athletes’ emotional involvement was strongly associated with betting problems whereas erroneous beliefs were not. However, distorted cognitions/ beliefs were associated with higher volumes and more frequent betting activities	These results highlight athletes’ emotional involvement and erroneous beliefs as potential targets for future intervention and prevention efforts
Jonsson et al.	2020	To examine outcomes in gambling expenditure over 12 months post intervention	Observational study following a three-arm randomized controlled trial A total of 1003 statistical triplets from the top 0.5% of customers based upon annual expenditure, matched on sex, age and net losses. Mean age was 53.4 years; 19% were women, mean yearly loss for 2016 was 88 197 NoK. Interventions and comparator Feedback intervention by telephone, letter or a no-contact control condition	Per-protocol analyses of triplets who received the telephone call or letter as randomly assigned (n = 596) showed a positive and sustained effect over 12 months: the telephone group showed a 30% reduction in theoretical loss (d = 0.44) and the letter group 13% (d = 0.18), both outperforming the control group with a 7% reduction (d = 0.11). The telephone condition was superior to both the letter and control conditions in per-protocol (P < 0.001) and to control condition in intention-to-treat analyses (ITT) (P < 0.001). Individuals in the telephone condition took more responsible gambling actions. The letter condition had better outcomes than the control in the ITT-only analysis (P < 0.001). More than 93% were still customers a year after the intervention	Secondary prevention—Personal contact with high-expenditure gambling customers in Norway that provided individualized feedback on expenditures was associated with reduced theoretical losses and greater use of responsible gambling tools over a 12-month period, compared with no contact. Telephone intervention with customers had a larger impact than a mailed letter
Jun et al.	2021	To test whether earlier depressive symptoms, antisocial behaviors, and alcohol use predict later gambling participation and problem gambling in emerging adulthood in order to inform prevention and intervention strategies	Longitudinal school-based survey of individual, family, school, neighborhood, and community influences regarding physical and psychological health, health-related behaviors, and outcomes. Data from waves 1, 3 and 4 were analysed. The final data analytic sample included 8282 cases of emerging adults ages 18–29	Earlier antisocial behaviors and gambling behaviors increased later risk for gambling participation and problem gambling. Past-year alcohol use and heavy drinking were associated with the increased risk of gambling participation but not problem gambling. Earlier depressive symptoms decreased the risk of gambling participation later among those who endorsed antisocial behaviors	Earlier G behaviors were the best predictor of later G behaviors. G in emerging adults remains mostly stable in this transitional period
Jun et al.	2019	To explore gender differences in the prospective relationships among depression, antisocial behavior, alcohol use, and gambling behaviors in emerging adulthood	DATA SOURCE: National Longitudinal Study of Adolescent to Adult Health (Add Health). SAMPLE: Respondents ages 18–29 with valid longitudinal sampling weights for the data in Waves I, III, and IV (n = 15,701), with a final study sample of 8282 emerging adults. MEASURES: G behav, depression, antisocial behav, alcohol use behav, sociodemo. ANALYSIS: Secondary data analysis	Antisocial behavior was associated with increased risk of alcohol use. Heavy drinking in early emerging adulthood was associated with increased risk of gambling later, but depression was marginally protective of gambling. Among men, contemporaneous associations between alcohol use and heavy drinking were stronger than among women. Among women, earlier binge drinking conferred increased risk of later gambling problems, but in men negative relationships between the two were found	Consistent with prior literature: a) antisocial behav assoc with alcohol use behavs in M and F. b) earlier heavy drinking assoc with later G participation BUT this was stronger for M compared to F (not consistent with previous research). Inconsistent with prior literature: a) experiencing earlier depression had a slightly lower likelihood of later G participation in M and F. (previous literature suggests depression is a signif factor in PG). GENDER DIFF IN BEHAV CORRELATES OF G: M show stronger assoc between past-year alcohol use and heaving drinking than F (consistent with prior). Earlier binge drinking is a risk factor for later G for F but not M (inconsistent with prior). Other gender differences were not significant
Kaltenegger et al.	2019	To examine the effects of impulsivity and psychological health on risk gambling and heavy episodic drinking (HED), and whether psychological health functions as a moderator / protective factor	Data was extracted from the Stockholm School Survey, collected in 2014 and 2016 among students in the ninth grade of primary school (15–16 years) and second grade of upper secondary school (17–18 years) in Stockholm (n = 21,886). Impulsivity, psychological problems, risk gambling, HED, and a number of sociodemographic control variables were measured using self-report data. The statistical method was binary logistic regression	Results showed that risk gambling (3.4%) and HED (22.8%) were prevalent among Swedish pupils. Impulsivity and—to a weaker extent—psychological problems as well as several sociodemographic variables were risk factors for risk gambling and HED. Furthermore, psychological problems negatively moderated the association between impulsivity and HED among girls	This study supports evidence that impulsivity represents a risk factor for risk behaviors, and—contrary to the a priori hypothesis—indicates that the association between impulsivity and HED in female students might be attenuated by the presence of psychological problems. Prevention measures should particularly address adolescents exhibiting the mentioned risk factors and aim at reducing psychological problems, but not necessarily target the adolescents showing impulsivity and psychological problems simultaneously
Kang et al.	2019	To investigate the gambling factors related with the gambling problem level of adolescents to provide basic information for the prevention of adolescent gambling problems	The data was drawn from the 2015 Survey on Youth Gambling Problems of the Korea Center on Gambling Problems for Korean students in grades 7–11 (ages 13–17 years) and included 14,011 study subjects (average age 14.9 years, 52.5% male) Measures: CAGI;	The lifetime gambling behavior experience was 42.1%, and 24.2% had a gambling behavior experience within the past three months. The past three-month prevalence of problem gambling was 1.1%. The gambling factors related with the level of adolescent problem gambling include the presence of nearby gambling facilities, having personal relationships with people that gamble, a higher number of experienced gambling behaviors, male adolescents, and a greater amount of time spent gambling	These findings suggest that gambling prevention efforts must consider not only access to individual adolescents as early intervention, but also environmental strategies such as accessibility regulations and alternative activities
Kang et al.	2020	This study aimed to identify patterns of gambling activities and factors related to specific subgroups of out-of-school adolescent gambling activities	Analyzed secondary data from the 2015 Korea Youth Gambling Problem Survey, including 1,200 out-of-school adolescents. Latent class analysis was conducted to identify patterns of gambling activities. The factors related to gambling subgroups were verified with multinomial logistic regression gambling severity measured using Canadian Adolescent Gambling Index (CAGI)	Three latent classes of gambling activities were identified: rarely gambling (RG), immediate gain gambling (IGG), and broad gambling (BG). These subgroups differed significantly in terms of gender, age at and type of first gambling experience, number and type of gambling activities, gambling frequency, time and money spent on gambling, problem gambling severity, and motivation for gambling. Compared to the RG subgroup, both the IGG and BG subgroups were strongly associated with an older age at the first gambling experience	Out-of-school adolescents who first gambled at an older age and who gambled mainly in order to gain money immediately were at risk of problem gambling. Developing strategies for early screening and referral to professionals is necessary to prevent gambling problems from worsening
Keen et al.	2017	The aim of this review was to report the outcome of studies empirically evaluating gambling education programs across international jurisdictions	PRISMA	Many studies demonstrated change in knowledge, beliefs and attitudes do not translate to changes in behaviour. Limitation in evidence is reliance on cognitive measures as proxy for harm. Programmes that are based in sound theoretical parameters more likely to lead to observing behaviour change	
King et al.	2020		Confirmatory factor analysis to model how the symptom count of gambling fits into the structure of psychopathology in a large, community based young adult twin sample of men and women (age 24; N = 1329). Twins were assessed via in-person, structured diagnostic interviews on disorders including: Major Depression, Phobias, Post-Traumatic Stress Disorder and Anxiety Disorders (internalizing) and Substance Use Disorders, Gambling Problems (self-report), and Antisocial Behaviors (externalizing)	Gambling most likely in individuals with externalising behaviours (anti-social behaviour etc.)	Implications of these findings suggest that during emerging adulthood gambling problems are best classified and conceptualized in the realm of externalizing disorders for both males and females. Results also suggest prevention and intervention efforts be aimed at young adults who exhibit commonly co-occurring psychopathology
King et al.	2017	To examine the genetic and environmental influences on gambling behaviors contributing to stability and change from ages 18 to 25 in a longitudinal, behavioral genetic mixed-sex twin study design	We examined the genetic and environmental influences on gambling behaviors contributing to stability and change from ages 18 to 25 in a longitudinal, behavioral genetic mixed-sex twin study design. Participants were enrolled in the Minnesota Twin Family Study	A range of gambling behaviors (maximum frequency, average frequency, money lost and gambling problems) were assessed at ages 18 and 25. The results of our study support the following conclusions: (1) the genetic and environmental factors impacting a range of gambling behaviors are largely similar in men and women, (2) genetic factors increase in influence from 18 to 25 (21% at age 18 to 57% at age 25), (3) shared environmental factors are influential at age 18, but tend to decrease from ages 18 to 25 (55% at age 18 to 10% at age 25), and (4) non-shared environmental influences are similarly significant and are small to moderate in magnitude at both ages	The findings add to a small, yet important research area regarding determinants of youth gambling behaviors and have the potential to inform prevention and intervention efforts
Knaebe et al.	2019	The current study sought to subtype current-, past-, and non-problem gamblers using three common comorbidities; psychological distress, risky alcohol use, and impulsivity. Participants’ endorsement of helpful behaviour change strategies was also examined by subtype membership	The current study sought to subtype current-, past-, and non-problem gamblers using three common comorbidities; psychological distress, risky alcohol use, and impulsivity. Participants’ endorsement of helpful behaviour change strategies was also examined by subtype membership	The most helpful change strategies for current and past gamblers were similar across subtypes (i.e., accept that gambling needs to change, remind yourself of the negative consequences). Non-problem gamblers reported the most helpful strategy as setting financial limits. This study indicated that treatment of psychological distress, risky alcohol use or impulsivity may be important for all gamblers regardless of their level of risk	Most helpful strategy for current problem gambling were cognitive and related to planning past-problem gamblers with high impulsivity endorsed planning and financial restriction (leve cards at home)
Kolandi-Matchett et al.	2018	To determine implementation effectiveness of two gambling-related harm minimisation programmes to inform improvement and future program planning	Document analysis, staff survey and staff focus group discussions	The programmes demonstrated capacity to not only achieve expected outcomes (e.g. enhanced community awareness about harmful gambling), but also to enhance social sustainability at the community level (e.g. established trustful relationships) and achieve some programme sustainability (e.g. community ownership over ongoing programme delivery). C	Community-action based harm-minimisation programmes offer programme sustainability potential which in turn offers funding cost-effectiveness when there are continual Public Health outcomes beyond initial funding. Although resource intensive, the community-action based approach enables culturally appropriate Public Health programmes suitable for societies where specific ethnic groups have higher gambling risk. Recognition of such harm-minimisation programmes’ contribution to social sustainability is important considering the potential for broader Public Health outcomes (e.g. better life quality, lesser social problems) within socially sustainable societies
Kotter et al.	2019	To provide a comprehensive summary of (1) the demographic characteristics of land-based self-excluders and changes after exclusion, including (2) gambling behavior, (3) gambling problems, (4) mental symptoms, and (5) mental health		Self-excluders were predominantly men in their early or middle forties. Changes after exclusion revealed wide ranges in the rates of abstinence (13–81%), rates of gambling reduction (29–92%), and rates of exclusion breaches (8–59%). The records consistently demonstrated significant changes in pathological gambling from before exclusion (61–95%) to after exclusion (13–26%). Up to 73% of self-excluders exhibited symptoms of anxiety, depression, and substance use disorders at program enrolment. Several aspects of mental health improved after exclusion, e.g., quality of life. Problem and pathological gambling are most prevalent in young men, but self-exclusion was most prominent in middle-aged men. The magnitude of effects widely differed between studies despite overall benefits of self-exclusion, and many individuals continued gambling after exclusion	High rates of pathological gambling and other mental disorders in self-excluders highlight the need for improved early detection and treatment accessibility
Kotter et al.	2018	The present study retrospectively investigated (1) the role of voluntariness of exclusion for the first time, and (2) general gambling behavior of excluded individuals before and after exclusion	A total of N = 215 casino excluders (self-excluders: n = 187, forced excluders: n = 28) completed an online survey or a face-to-face interview up to 8 years after enrolment	Self- and forced excluders showed similar rates of abstinence (self-excluders: 19.3%, forced excluders: 28.6%) and reduction (self-excluders: 67.4%, forced excluders: 60.7%), even though forced excluders reported a significantly greater initial gambling intensity compared to self-excluders (e.g., pre-exclusion gambling time; self-excluders: 3.2 days/week, forced excluders: 4.3 days/week). Overall, results indicated that 20.5% of excluders stopped all gambling activities and another 66.5% reduced their gambling. Those who continued gambling significantly reduced this behavior in every segment, except for gambling halls	Findings indicate that self- and forced exclusion are associated with similarly reduced gambling behavior, even in non-excluded segments. However, unchanged gambling in gambling halls emphasizes the importance to implement consistent exclusion programs over all gambling segments
Ladouceur et al.	2017	The purpose of this research is to identify empirically grounded RG studies in an effort to create the beginnings of a foundation that can guide evidenced based effective RG strategies	Systematic review studies were retained if they met two criteria: (1) a specific focus on RG related topics and (2) evidence of an empirical approach toward evaluating RG strategies, initiatives, programs or theoretical content	There were 105 publications that were retained for further analysis Five RG strategies referred to in examined literature: **Self-exclusion:** limited take up and only effective in the short term but no data around long-term effectiveness. Some programs effective over 6 months if combined with individualised follow-up **RG behavioural characteristics:** difficulties in tracking gamblers behaviours impacts quality of data. Gambling frequency, expenditure, and duration of gambling strongly linked to likelihood of problem gambling **Setting gambling limits:** some evidence that setting monetary limits more effective than time limits. Variable success across all types of self determined limit setting **RG specific game features:** structural features that promote RG. features can include warning messages on EMG screens. More effective if in middle of screen in terms of recall of messages. Cash display more effective than pre-commitment or visible clock. Graphic messages more effective than text only messages **Venue staff training for intervening with problem gamblers:** minimally effective in terms of staff ability to recognise patrons at-risk of problems with obvious disparities in self-reported PGSI scores and estimates by staff. Staff unlikely to discuss issues of problem gambling with patrons or approach patrons due to personal discomfort	Minimal empirical evidence to inform RG approaches to gambling harm but sufficient to help create evidence-based RG interventions
Langham et al.	2017	To examine the relationship of individuals’ sense of coherence on their gambling behaviour and experience of gambling-related harm	This exploratory study utilised an archival dataset (n = 1236) from an online, cross sectional survey of people who had experienced negative consequences from gambling	In general, a stronger sense of coherence was related to lower problem gambling severity. When gambling behaviour was controlled for, sense of coherence was significantly related to the experience of individual gambling harms. A strong sense of coherence can be seen as a protective factor against problematic gambling behaviour, and subsequent gambling-related harms	The present study demonstrates the potential value of, and provides clear direction for, considering sense of coherence in order to understand gambling-related issues
Latvala et al.	2018	To explore the associations between final compulsory school grades and gambling and their relation to substance use and perceived mental health among people aged 18–29 in Finland (N = 831)	Cross-sectional random sample data, weighted on the basis of age, gender and region of residence, were collected in 2015. The data were analysed using logistic regression models adjusted for sociodemographic variables, risky alcohol use, daily smoking, and perceived mental health	Weekly gambling and at-risk and problem gambling (ARPG) were more common among men. Weekly gambling was linked to smoking and risky alcohol use among men and smoking among women. Additionally, ARPG was linked to risky alcohol use among men. ARPG was associated with moderate/poor mental health among men and women, but this was not the case with weekly gambling. Among men, low and average final school grades at age 16 were associated with weekly gambling later in life, even when adjusting for other variables. Among women, low and average final school grades were not associated with weekly gambling when adjusting for substance use. Lower final school grades were associated with ARPG among women but not among men when all potential confounders were adjusted for	Adolescents with lower final school grades are more likely to gamble weekly later in life. Lower final school grades are also linked with ARPG among women. It is important therefore for schools to have clear policies on gambling and to implement early prevention programmes
Levesque et al.	2018	This study assesses the mediating effect of cognitive distortions between psychological vulnerability (personality and mood), and gambling problem severity. It also verifies whether the relationships between these variables differs according to the preferred gambling activity	The sample is composed of 272 male gamblers [191 poker players; 81 video lottery terminal (VLT) players] aged between 18 and 82 years (M = 35.2)	Bootstrap analysis results revealed that cognitive distortions mediate the effect of narcissism on gambling problem severity for both groups. The level of depression for VLT players significantly predicted gambling problem severity, both directly and indirectly via the mediating effect of cognitive distortions. Mediation analyses also indicated that narcissism had an indirect impact on problem gambling through cognitive distortions for both groups	
Lostutter et al.	2019	to investigate the relationship between money attitudes, gambling behaviors and disordered gambling severity among college students	Final sample of 2534 undergraduate psych students. Measures: *Money Attitudes Scale* (subscales a) power-prestige, b) retention-time c) distrust d) anxiety), *frequency and quantity of G* (single items), *G-related consequences*, *severity of G prob*s (modified South Oaks G Screen). Analysis Plan: The hurdle model fits all zeros in a logistic regression portion of the model; non-zero responses are included in a truncated count regression model. This approach allowed simultaneous examination of effects of G attitudes on the rate of any G outcomes as well as on the intensity of gambling involvement among those reporting at least one experience of the outcome	Results suggest that college students who hold high Power-Prestige or Anxiety attitudes toward money were more likely to gamble and experience greater consequences related to their gambling. Distrust attitudes were negatively associated with gambling behaviors. Retention-Time attitudes were not significantly associated with gambling behaviors and may not be directly relevant to college students, given their often limited fiscal circumstances	Suggest power-prestige attitudes toward money may be a risk factor for gambling involvement and gambling disorder among college students. Suggest individuals higher on anxiety attitudes toward money may view gambling as a means of increasing their financial security, and may persist in gambling as a result. Popular media portrays gambling as means to gain money, power and status. This portrayal may perpetuate gambling-related cognitive distortions and encourage individuals to continue gambling despite the negative consequences in the hopes of achieving success. Suggests that a distrustful perspective toward money may serve as a protective or limiting factor for gambling. Suggest retention attitudes toward money may be a protective factor for engaging in any gambling, perhaps due to the recognition that gambling poses a risk to financial security. Retention attitudes do not appear to be protective against problematic levels of gambling involvement (MAS scale for retention ma not be appropriate for college students due to focus on financial planning)
MacArthur et al.	2018	To examine the effects of interventions implemented up to 18 years of age for the primary or secondary prevention of multiple risk behaviours (tobacco use, alcohol use, illicit drug use, gambling, self-harm, vehicle-risk behaviours, antisocial behaviour, sexual risk behaviour, physical inactivity, and poor nutrition) among individuals aged eight to 25 years	Included randomised controlled trials (RCTs), including cluster RCTs, which **aimed to address at least two risk behaviours**. included only studies with a combined intervention and follow-up period of six months or longer. We excluded interventions aimed at individuals with clinically diagnosed disorders along with clinical interventions. We categorised interventions according to whether they were conducted at the individual level; the family level; or the school level	We included in the review a total of 70 eligible studies, of which a substantial proportion were universal school-based studies (n = 28; 40%). Most studies were conducted in the USA (n = 55; 79%). On average, studies aimed to prevent four of the primary behaviours. Behaviours that were most frequently addressed included alcohol use (n = 55), drug use (n = 53), and/or antisocial behaviour (n = 53), followed by tobacco use (n = 42). **No studies aimed to prevent self-harm or gambling alongside other behaviours**	
Macia et al.	2022	1) To explore the differences between groups of problem and non-problem gamblers in attachment, gambling motives (social, enhancement and coping), positive and negative affect, and addictive behaviours (gambling, drugs, spending, alcohol and video games). (2) to analyse the relationship between these variables in the subsample of at-risk and possible problem gamblers. (3) to examine the predictive role of positive and negative affect, gambling motives, and attachment in the aforementioned addictive behaviours	Survey data collected from convenience sample from secondary colleges, professional training centres and treatment centers for PG. 351 young women and adol. Two sub-samples based on SOGS: (1) the group of PGs included women at risk or presenting possible problem gambling from both the association centres and the general sample, i.e. subclinical sample (2) the group of women without G prob from the general sample. Measures: SOGS-RA, drugs, alcohol, video games and spending, attachment, gambling motives, affect,	Females at risk or with problematic gambling scored higher on gambling, drugs, compulsive spending, maternal attachment, and gambling motives (enhancement, social and coping motives). Effect sizes where signif diff found, large for G, spending and enhancement motives, medium for drugs, mother attachment and social and coping motives. G correlated positively with drugs, spending, negative affect (now), and enhancement and coping motives, and correlated negatively with maternal attachment. Hierarchical regression found that G was associated with social and coping motives	Young women who met the criteria for risk or possible problem gambling presented significantly higher differences in gambling, spending and enhancement gambling motives, and slightly higher in the case of coping motives. Results are consistent with previous studies showing that patterns of an insecure attachment style are related to the symptomatic expression of risky addictive behaviours in adolescents, as well as to increased susceptibility for the development of these behaviours in adulthood. Identification of the possible vulnerability factors for each addiction could be useful in the design of prevention and treatment approaches. In addition, the need for integrated and holistic health- and social- care programmes are suggested in terms of sex and age
MacLean et al.	2019	To develop an understanding of the social practice of gambling in two regional Aboriginal communities in the Australian state of Victoria	Two Aboriginal community-controlled health services commissioned collaborative exploratory research into community experiences of G. Focus of 1 service on kinds of gambling played in their community, the nature of any associated harms, why relatively few community members accessed support services to deal with gambling, and what, if anything, community members believe needed to be done in response to gambling harms. Other service focused on similar issues but specifically for young Aboriginal people aged 16–25. Interviews with 50 First Nation Peoples, who had personal experience of gambling, had been affected by another person’s gambling, or had professional experience working with gamblers. Interpreted using Social Practice Theory and coded to the elements of "meaning', 'material', 'competence' and 'temporality'	**Meaning:** Complex and sometimes contradictory meanings. It was regarded as providing important opportunities for social engagement, bringing people together in an environment which usually felt safe and welcoming. Yet at the same time, gambling brought conflict, isolation from others and shame. G recognised as a means to reinforce a sense of community. Social aspect particularly emphasised by older women—respite from dealing with family issues and caring responsibilities. G provided entertainment and social outing. G beneficial in enabling staying away from alcohol and to spend time with family. Bingo was benign, pokes as highly dangerous (and eroded relationships). When money was lost, family conflict and violence could be among the consequences. G lead to self-disgust and shame (and unwillingness to use help services). **Material:** Hope of financial gain is powerful incentive. In Indigenous cultures based on collectively and sharing of resources, gambling losses have a wide effect beyond the individual. Felt welcomed into G venues when spending money. Venues welcoming with feed, TV etc. Pokies was considered physically and psychologically addictive. Online G was most common for young people—easy to spend money when not using cash. **Competence: G alleviates boredom. helped manage negative emotions and cope with adverse experiences (albeit temporary). Children learn to G and consider G as routine part of life from a very young age. G took time that could be used to develop competence in Aboriginal cultural practices. Temporal: Sense of appreciation for inclusion in venues where historically were excluded. As a consequence informal G became an important part of community life. *Participants argued strongly that restricing avail of G venues and hours of operation was a critical element in reducing harms***	Positive and negative effects of G are intertwined and difficult to extricate from each other in the social practice of gambling in Aboriginal communities. Changing meanings of gambling for Aboriginal people (or indeed for anyone) is a complex undertaking and our participants recommended providing opportunities for discussion within communities to reconsider the role of gambling in community life. Argued for culturally-based community-focused responses (i.e. yarning circles, which fulfil G functions). Address material elements of harmful gambling by closing or restraining growth in gambling venues, limiting hours of access, and better regulating internet gambling and gambling advertising. Provision of alternative activities and education about G harms, encouraging adults to teach children games that don't involve G. Restrict gambling and give meaningful ways to spend time and engage with community
Mader et al.	2019	Limited tests of etiological suppositions on which the Pathways Model is founded upon. To test if the classes yielded by a latent class analysis (LCA) matched the Pathways Model subtypes in a community sample of adult first-time problem gamblers	125 first-time adult **problem gamblers** who participated in the Quinte Longitudinal Study of G and PG. First-time problem gamblers were defined as those who did not endorse lifetime history of problem gambling at Wave 1 and met the criteria for problem or pathological gambling. Indicators in analysis: impulsivity, social dysfunction, antisociality, premorbid depression, problematic substance use and anxiety related disorders	A three-class solution was found to be the best fitting model. There was a class resembling **Behaviourally Conditioned** gambler (n = 40), demonstrating minimal rates of pre-existing psychopathology and social dysfunction; a class that shared consistencies with the **Emotionally Vulnerable** gambler (n = 56), showing intermediate rates of anxiety, depression, problematic substance use and social dysfunction prior to onset of problem gambling; and a class resembling the A**nti-social Impulsivist gambler** (n = 29) characterized by the highest impulsivity, social dysfunction, antisociality and pre-existing psychiatric illness	Provide evidence for the etiological assumptions of the Pathways Model by demonstrating that problem gamblers can be sub-grouped on traits pre-existing the development of aberrant gambling. Findings support the theory that problem gamblers can be subdivided into three groups based on factors that predate problem gambling. Furthermore, the latent classes found in this study share important similarities with the Pathways Model, lending further support to its etiological assumptions
Mallorqui-Bague	2018	To identify **specific GD subtypes** according to different domains of EFs (including cognitive flexibility, inhibition and working memory as well as decision making) in a population of men seeking outpatient treatment for GD. To characterize the resulting subtypes by exploring and comparing their sociodemographic variables, comorbid clinical symptoms, as well as personality and impulsivity traits	145 males ranging from 18 to 65 years **diagnosed with GD.** Clinical and neuropsychological assessment, self-reported measures of impulsivity, personality and psychopathological state	Cluster 1 [n = 106; labeled as Low Impaired Executive Function (LIEF)] was composed by patients with poor results in the neuropsychological assessment; cluster 2 patients [n = 46; labelled as High Impaired Executive Function (HIEF)] presented significantly higher deficits on the assessed domains and performed worse than the ones of LIEF cluster. patients in cluster 2 were significantly older, unemployed and registered higher mean age of GD onset than patients in cluster 1. Additionally, patients in cluster 2 also obtained higher psychopathological symptoms, impulsivity (in both positive and negative urgency as well as sensation seeking) and some specific personality traits (higher harm avoidance as well as lower self-directedness and cooperativeness) than patients in cluster 1	To characterize the resulting subtypes by exploring and comparing their sociodemographic variables, comorbid clinical symptoms, as well as personality and impulsivity traits
Maltzahn et al.	2022	To identify regulatory, policy and program measures to address gambling harm to bingo players and their communities and, in doing so, extend existing Public Health approaches to gambling to better include bingo	Data were collected between September 2018 and October 2019 through 12 participant observation sessions of bingo games, 53 individual, pair and group interviews with bingo players and 13 individual and pair stakeholders interviewers. Interview stakeholders—community workers, G H treatment staff, gov reps, regulatory experts, bingo operators. Snowballing. Interview focus: experiences of and observations about bingo playing and recommendations about what, if anything, needed to change related to bingo. Observation sites/ participants recruited via criterion sampling. After initial analysis, themes and recommendations were shared and tested with participants and other experts, both through site-specific feedback sessions and via a symposium with gambling experts. Case studies—thematic analysis	**Drivers of and influences on harm:** Bingo—low risk, enjoyable, affordable, inherent protective factors (i.e. group based, st price, time limit). Attractive to some because of protective factors. Also used personal strategies to further limit possible harm. Strong community connectedness was further protection—seek help from family (among Pacific community). No need to change traditional bingo. **Technological, regulatory and commercial changes to bingo eroding protective factors**: Also recog. G harms. Some believed G industry preyed on vulnerabilities. Concerned that protective were being eroded, therefore impacting on personal harm-min. strategies. Factors making higher-risk—close to EGMs, intro of PETs (personal electronic tablets), increased game costs, prizes and crowd sizes, linked jackpots (across sites and sessions). Also weak compliance processes. **Bingo used to bolster other gambling for commercial purposes**: Bingo entices involvement in other forms of G. **Government reluctance to increase regulation of bingo**: Believed change was needed but doubted gov would curtail G. Lack of faith in G around G. **Not recognising different experiences of subgroups**: Pacific communities—bingo sometimes used as fundraiser for community of origin in Pacific (i.e. linking bingo to community minded). Conversely considered in sin in churches attended. Strong community ties—> limited help seeking from services. Immigrants not help seeking due to language barriers. Involvement in G driven by limited access to other wealth gaining opportunities. **External influences on gambling harm**: including poverty, precarity and pre-existing trauma. **Participant recommendations:** safeguarding bingo's protective factors, delinking bingo from other forms of G, addressing factors external to G	Suggest 5 sets of measures that extent existing **Public Health approaches** to G to better encompass bingo. (1) Safeguard bingo's protective features (2) Delink bingo from gambling ecosystem (3) Tailor strategies for subpopulations (4) Address external harm contributors and facilitate recovery (5) Dismantle political protection of gambling industry
Maltzahn et al.	2018	Exploratory study designed to understand the complex benefits and harms associated with bingo playing for Aboriginal people in Sunraysia, a regional community in Victoria, Australia. To understand people’s experiences and perspectives on gambling and related issues, rather than to quantify or assess their potential role as gamblers	26 interviews with First Nation peoples (11 who were workers at Aboriginal community controlled health and family service. Participatory, collaborative research	Bingo as a space for Community Social Life and Pleasure. Popular with women and older people, but viewed as intergenerational activity; fun, shared experience and connection. Social = positive. As such not perceived as G. Winning $ was secondary to social connection and fun. Venues were part of bingos attraction. Bingo as an Escape Provided respite from hardship etc. Provides a distraction. Provided kinship with people experiencing the same struggles. But for some, also provided opportunity NOT to be with people facing similar circumstances. As bingo in alcohol free space—> limiting drinking, which avoided possible flow on to family violence Bingo, Risk Management and Risk Exposure Considered a safe form of gambling, where social benefits outweighed harm. Other viewed as a pathway to EGM. Bingo described as a way to manage harm i.e. protection from excessive spending on other G. Inherent boundaries/limits in bingo—book price, time, prize amount—> predictable and manageable. Controlled spend. Alternative to unmanageable forms of G but still offering excitement and entertainment. Others saw G as harming individuals and corroding community—seen as equally harmful as other forms of G. Free bingo in commercial venues lured to EGM. Bingo has become modernised and hi tech with larger winnings	Bingo reinforces social connectedness and offers fun and excitement, a strategy to find solace or respite in the face of personal pain (such as violence, poverty and histories of incarceration), and marginalization. Bingo can at times, under some circumstances, provide a less risky form of gambling than playing electronic poker machines. It can offer respite from alcohol consumption and its effects. The cost of bingo can be heavy for people with significant family commitments and low, fixed incomes, and can unnecessarily expose people to encouragement to use electronic poker machines
Marinaci et al.	2021	Aim: To explore the role of the qualities of relational networks (i.e. family functioning, perceived social and class support), family and peer approval and view of the social environment in predicting problem gambling, problem gaming and overall well-being among adolescents	Recruitment impacted by COVID. Final sample: 3 secondary schools. Random selection of 2–3 classes b/w 9 and 12 Grade at each school. 595 students aged b/w 13–18 yrs. Measures: Problem G Severity Index, Gaming Addiction Scale, wellbeing, parental monitoring, G approval by family members and peers, family functioning, social support, class support—classmates and teachers, view of the social environment. Surveys administered via Google Forms	Prevalence: 16.3% low risk, 6.6% moderate risk, 2% PG. Video game addiction 0.5% and 5% depending on analysis. Multivariate multiple regressions identify a common core underpinning problem gambling, gaming and poor well-being but also the distinct roles of psychosocial variables: being male, with low parental monitoring, and an anomic view of the social environment all predict problem gambling and gaming, which were also found to be associated. Low social support predicts problem gambling but not problem gaming; poor family functioning predicts problem gaming but not problem gambling. All the target psychosocial variables, except approval of gambling, predict poor well-being	1) Highlights the importance of taking into account the role of the interpersonal and social sphere along with the other dimensions (e.g. neurobiological, temperament and personality's traits) related to the individual sphere (2) Highlights specificities in the psychosocial paths which influence G, gaming and wellbeing. Poor family functioning seems crucial to explaining problem gaming, which was also found to be associated with poor well-being. Fndings suggest how important the perception of a meaningful, reliable context may be for adolescents’ well-being and for preventing problem consumption of gambling and gaming. The feeling of living in an anomic context where being lucky is more important than acquiring knowledge to construct one's future is a dramatic risk factor emerging in the current study
Marionneau & Jarvinen-Tassopoulos	2022	To investigates gamblers’ and their concerned significant others’ (CSOs) experiences and views on treatment and help services during spring 2020 in Finland, and their suggestions for how services and prevention should be (better) organised during and after **COVID-19**	Data collected with three separate online questionnaires (conducted by a university, help service, and harm prevention services) during spring 2020 in Finland. Aim of surveys to chart changes in G during closures of G opportunities as well as treatment and help services. N = 847 (688 gamblers, 97 concerned signif others (CSO) and 62 as both	**Changed needs for treatment or help services during the COVID-19 lockdown**: Total consumption declined but online G maintained at existing levels or increased in some contexts. Also overall reduced need for Tx and help both Gers and CSOs. Expressed relief at closures, particularly EGMs. Expressed need for additional or improved services to maintain positive effects of G closures and to address possible shift towards online gambling or increased gambling participation of online gamblers. **Impacts of service closures and the transfer of services online:** Respondents whose service discontinued: Particularly CSOs—concerned over changes/closures and how affect Gers. Experienced increased harms and considered support was weak. Respondents whose service moved online: Not as useful as face to face. Others getting used to meeting online. Online provided additional channels for keeping in touch (i.e. closed groups on WhatsApp, email or messaging). Regular messages were useful to maintain abstinence. **Suggestions for improving existing help services during and after COVID-19:** COVID specific: Online tools such as regular support messaging to be maintained. Long term: Professionals to raise topic of G and integration of G addiction support in Public Healthcare to improve service availability. In additional to professional help, peer support and support person contacts. Other respondents were happy to the services received. **Improved harm prevention during and after COVID-1**9: Suggestions for overall prevention of harm fell into four general categories: **information campaigns (RE EGM—Content—proper warnings on what G can lead to. Campaigns focus on informing about the odds of winning, informing how G habits changed during COVID, dangers and addicivtiy of online G, gaining visibility in social media, newspaper, TV, depending on demographic**), limit setting, availability restrictions also after the pandemic, and a Public Health or welfare policy approach to gambling	1) While service closures were, unsurprisingly, experienced in a negative light, transfers online were, more surprisingly, considered mainly positively (2) help and treatment services should also be more tightly integrated into wider health, social and welfare service agendas (3) the importance of preventive work rather than treatment of those already inflicted with harm. Support tackling gambling harms as a Public Health issue at all levels
Martinez-Loredo et al.	2019	To identify subpopulations of adolescents using different substances and gambling activities, to explore gender differences and to examine impulsivity as a predictor of class membership	1691 adolescents surveyed. Measures: substance use, G behav, severity of alcohol use and G, self-reported impulisivity, delay discounting	Based on a latent class model of drugs and gambling activities, four subpopulations of males (smokers with alcohol abuse, exclusively G, non-users and broad users) and five of females were found (exclusively alcohol users, broad users, non users, alcohol and gambling users and smokers with alcohol abuse. Male exclusive G had a high probability of G in lottery, sports and scratch tickets and broad users had high probability of drugs and poker, sports betting, lottery, scratch tickets and EGM. Female broad users has a high probability of lottery and scratch card users. Male broad users more likely to experience PG but not female board users. General impulsivity and sensation seeking were the most consistent predictors of class membership	Most adolescents of either sex did not engage in any addictive behavior (i.e., ‘non-users’ class) and a minority of them reported concurrent use of different substances or gambling activities (i.e., ‘broad users’ class). The identification of gender-specific gambling activities associated with ATC use among the ‘broad users’ represents a novel finding. Despite sensation seeking and impulsivity being significant predictors of class membership across subpopulations, they were more relevant for some groups than others
Mathieu et al.	2018	To assess gambling practice, gambling motives and cognitive distortions in terms of their presence and nature in gamblers, to compare them between the different types of gamblers, to determine the link between motivations and cognitions, and also to explain the nature of the relationship between gambling motivations and cognitive distortions	Recruitment through online G forums. Participants G at least once a week. 259 male poker players responded to all surveys—24% non-PG, 61.7 at risk G and 14.3% PG. Measures: sociodemographic and gaming data, South Oaks G Screen, gambling motives, gambling-related cognitions,	Cognitive distortions were independently predicted by overall gambling motives (34.8%) and problem gambling (22.4%) (p\.05). PG had higher scores than at risk G and non-PG for overall G motives and for enhancement, coping, financial subscales but not social subscale. PPG had higher scores than at risk and non-PG for overall G cognitions and predictive control, illusion of control, interpretative bias, G expectancies and inability to stop subscales. **Multivariate model** accounted for 39.7% of variance of cognitive distortions with overall G motives and PG positively related to cognitive distortions	Cognitive distortions are a good discriminating factor of gambling problems, showing a close inter-relationship between gambling motives, cognitive distortions and the severity of gambling. These data are consistent with the following theoretical process model: gambling motives lead individuals to practice and repeat the gambling experience, which may lead them to develop cognitive distortions, which in turn favor problem gambling
Mazar et al.	2020	To further elaborate understanding of the relationship between **problem gambling**, forms of gambling, gambling involvement, and gambling intensity. H1: Problem gambling is more closely related to some gambling formats H2: Problem gambling is positively related to high involvement in gambling H3: Involvement in gambling is positively related to intensity of gambling H4:Gambling format mediates the relationship between involvement and problem gambling	Sample of 5852 adults who G at least once in past 12 mths. Final sample of PG n = 446. Baseline General Population Survey (BGPS) and the Baseline Online Panel Survey (BOPS)	Groups of monthly + gamblers participating in casino gambling, bingo, and sports betting contained a higher proportion of problem gamblers. High gambling involvement was also positively associated with problem gambling; however, a large minority of gamblers experienced problems when engaging in only one or two forms of gambling. Gambling involvement was also positively associated with intensity of gambling. Therefore, intensity of gambling may be partly driving the relationship between involvement and problem gambling. Specific gambling formats mediated the relationship between involvement and problem gambling	The gambling format an individual participates in is connected to whether an individual is likely to experience problem gambling. We also found that the level of involvement (and its relationship to intensity) may affect the likelihood that an individual will experience problematic gambling behavior. Ultimately, the type of gambling format an individual partakes in does mediate the relationship between problem gambling and involvement
Mazar et al.	2018	To understand the variables which discriminate between recreational gambling and at-risk gambling and whether they are similar or different to the ones correlated with problem gambling	Sample of 9523 adults. Baseline General Population Survey that established baseline level of G participation and PG prevalence and awareness/utilisation of PG services. Address based probability sampling. Questionnaire included sections on recreation, physical, and mental health behaviors, alcohol and drug use, attitudes toward gambling, gambling participation, gambling motivations, awareness of problem gambling services, gambling-related problems, and demographics. Problem and Pathological G Measure used to categorise respondents as Non-G (n = 2523), Recreational G (n = 6271), At Risk G (n = 600) and PG (n = 129)	The strongest discriminator of being a Non-Gambler rather than a Recreational Gambler was having a lower portion of friends and family that were regular gamblers. Compared to Recreational Gamblers, At-Risk Gamblers were more likely to: gamble at casinos; play the instant and daily lottery; be male; gamble online; and be born outside the United States. Compared to Recreational Gamblers, Problem and Pathological Gamblers were more likely to: play the daily lottery; be Black; gamble at casinos; be male; gamble online; and play the instant lottery. Importantly, having a greater portion of friends and family who were regular gamblers was the second strongest correlate of being both an At-Risk Gambler and Problem/Pathological Gambler	Novel study. Gambling involvement of family and friends is strongly related to Recreational Gambling, At-Risk Gambling, and Problem/Pathological Gambling. This suggests that targeting the social networks of heavily involved Recreational Gamblers and At-Risk Gamblers (in addition to Problem/Pathological Gamblers) could be an important focus of efforts in problem gambling prevention. Discriminating between Recreational and At-Risk Gamblers also shows the importance of social networks in relation to gambling behavior. Indeed, the portion of friends and family gambling regularly was the second strongest discriminator of at-risk gambling
McCarthy et al.	2020	To understand the range of factors that may influence the normalisation of gambling for young women	Part of a broader qual study exploring gambling attitudes and consumption behaviours of 18–34-year old young women in Australia. Guided by Constructionist Grounded Theory. 45 interviews with young women aged 18 to 34 years who G at least once in the past 12 months	**Early experiences with gambling: family rituals, traditions and behaviours**: Attending G venues for family occasions, informal gambling with family before legal age, part of family trad ie. Melb Cup. Some participated in G with fathers. Early experiences shaped knowledge of and confidence in G. Rituals associated with turning 18 YO—family gave $ for G, celebrated at G venue. **Influence of peer attitudes and behaviours**: Peers groups could socially engage with G as a shared experience. Preferred G products with a social element. G practices in social groups was gendered, even in mix-gender peer groups. Only exception was G with partner. Some pressured to confirm to social norms related to G in social groups. **Feminised gambling environments**: Casinos, horse racing, community G venues were female friendly due to perceived glamour and sense of occasion. G as part of other social activities—> normalisation. G venues accessible and available because open late. Co-located with other non-G activities. **Changing social attitudes towards women’s engagement in gambling**: G becoming more normalised for young women, even before legal age. G was entertainment but no consideration of risk. Availability and accessibility—> normalisation. Commercial marketing incentives	Range of factors that influenced and encouraged young women to G. Gambling-specific factors that may occur at an early age and may contribute to young women: (i) developing positive attitudes towards gambling; and (ii) engaging in either formal or informal gambling before the legal age. Parental practices raise qu about how parents perceive risks of exposure—no clear Public Health recommendations. Early exposure may—> normalisation. Reinforces calls for government policy and regulation that aims to remove gambling from everyday family environments. Behaviours developed before gambling was heavily promoted and easily available may now be problematic for young women
McCarthy et al.	2019	To review the evidence base on women’s gambling behaviours and experiences of harm	Drawing from strategies used effectively in other areas of Public Health, key elements for a gendered approach to harm prevention were identified and adapted into practical Public Health research, policy and practice strategies	Results indicated a lack of research that explores women’s gambling. Few studies have examined the impact of gambling on the lives of women, with limited understanding of the factors that influence women’s engagement with gambling products, and the impact of industry tactics. A gendered approach was identified as a strategy used successfully in other areas of Public Health to shift the focus onto women and to ensure they are considered in research. In tobacco control, increasing trends in women’s smoking behaviour were combatted with targeted research, policy and practical initiatives	A proposed framework for a gendered approach to gambling research, policy and practice provides regulatory direction and a research agenda to minimise gambling-related harm for women both in Australia and internationally. Evidence-based policies should be implemented to focus on the influence of gender and associated factors to address gambling-related harm. Practical interventions must take into account how women conceptualise and respond to gambling risk in order to develop specific harm prevention programs which respond to their needs
McMahon et al.	2019	To evaluate the systematic review evidence base on the effects of prevention and harm reduction interventions on gambling behaviours, and gambling-related harm. Also, to examine differential effects of interventions across socio-demographic groups	**Conceptual framework:** Combined two frameworks (1) Harm minimisation—(a) supply reduction, (b) demand reduction (c) harm reduction. (2) Capability-Opportunity-Motivation-Behaviour (COM-B) framework. **Inclusion criteria:** PICOS (population, intervention, comparison, outcome, and setting). **Search strategy:** 4 databases from 2018. Quality appraisal and data synthesis—MeaSurement Tool to Assess systematic Reviews (AMSTAR 2)	10 SR reporting on 55 unique relevant studies met criteria. Significant method. weaknesses in all studies according to AMSTAR 2. **Supply reduction Ix: r**educe opening hours. Caps on EGM**. Demand reduction Ix:** *Youth prevention Ix. 4 reviews identified 11 unique studies examining effective of Ix on youth G behave. 6/11 found no significant diff in G behave after Ix. Of 5 reporting significant effects, 4 studies reported a reduction in the number of gamblers, and gambling problems in Ix group. 2 studies reporting effect size, 1 of moderate quality found freq H in Ix grp reduced PG and Freq compared to non-freq G. Second study reporting effect size of weak quality, report medium reduction in at-risk/PG in Ix grp.* Smoking bans. **Harm reduction Ix:** Pre-commitment/limit setting. Self exclusion. Message messages/feedback. *Personalised feedback Ix. Both studies demonstrated that the personalised feedback intervention was more effective in changing behaviour compared to the cognitive intervention groups.* Removal of large not acceptors. Maximum bets. Removal of ATMs. No review extracted data or reported on the differential effects of intervention strategies across sociodemographic groups. Nine of the ten included reviews did not conduct an adequate quality assessment of primary studies in line with the AMSTAR 2 criteria	
Merkouris	2020	To develop and test the usability of one of the first smartphone app-delivered ecological momentary interventions for gambling (GAMBLINGLESS: CURB YOUR URGE), with key Australian stakeholders (ten consumers, nine gambling clinicians, and ten gambling researchers)	29 participants (10 consumers (G at least once every two weeks—past or present), 9 G clinicians, 10 G researchers) tested the usability of the intervention over one-week and reported on the content’s helpfulness, the ecological momentary assessments (EMA’s) relevance/burden, the Mobile App Rating Scale, and open-ended items assessing content and functionality. Participants were prompted via push notifications to complete an EMA twice daily—morning and evening. The EMA consisted of 12 core questions administered at each assessment time-point relating to gambling cravings and behaviors, self-efficacy, mood, readiness to change, and subjective alcohol intoxication. Up to an additional 14 questions were administered at each assessment where participants responded ‘Yes’ to questions asking them if they had a craving to gamble (currently or since the last notification) or they had gambled (since the last notification), to gain more detailed information. The EMA comprised a maximum of 26 questions which took 1–4 min to complete, depending on the pattern of responses	**Quantitative.** Preceived helpfulness of ix content: All activities rated above average helpfulness. 'Belly breathing', 'heavy breathing' and 'urge surfing' highest overall. Consumers and clinicians rated 'talk to someone' and 'tips to change your thoughts' as more helpful than researchers. PERCEIVED RELEVANCE AND BURDEN OF EMA QU: rated as average. Consumers rated EMA questions are more relevant than researchers. MOBILE APP RATING: met the minimally acceptable standard for app quality. App was easy to use, functioned as intended, and the information presented was of high quality. The aesthetic and engagement subscales were rated the lowest. **Qualitative.** acceptability of content: Liked info about nature of G cravings and focus of activities. Clinicians and researchers had specific views on how the acceptability of the app-delivered intervention could be improved: (1) increasing content variability, such as providing more concrete suggestions in relevant activities (e.g., Distract Yourself), and providing more mindfulness- and relaxation-based activities; (2) providing more depth to the content to build a stronger rationale for users to return to use them more than once (e.g., explain the benefits of practicing mindfulness); and (3) prompting more self-reflection. Clinicians also commented on the tone of the content and suggested that using more normalizing and reassuring language (e.g., normalizing the difficulty of reaching out to others in the Talk to Someone activity), as well as positively re-phrasing some content (e.g., focusing on the positive consequences of not gambling rather than the negative consequences of gambling in the Rationalize Your gambling activity) could increase its acceptability. PERCEIVED HELPFULNESS: All participants reported Ix could be helpful and effective for curbing cravings, particularly in the short-term. ENGAGEMENT: All participants reported liking the audio-visual components of activities, suggesting that they were interactive, engaging, and could be used in the moment. Engagement was an area for how activities could be improved. USABIILTY: All participants indicated that several activities were highly usable, particularly the Delay the Decision, Distract Yourself, Talk to Someone, and Reminder Card activities. Participants commented that they were simple, clear, easy to understand, and brief. This simplicity of design and interaction meant the app-delivered intervention could be used by people of all ages	The intervention content, helpfulness, and usability were rated highly in quantitative and qualitative assessments. Participants liked practical and instructive content but suggested that the intervention could be more engaging, interactive, and varied, and the number of EMA questions per timepoint could be reduced. All groups indicated that they would recommend this app, as it could increase knowledge, attitudes, awareness, behaviour change, intention to change, and help-seeking for gambling cravings
Michalska et al.	2020	To preliminarily explore online poker gamblers’ attitudes concerning harm reduction (HR) measures. Besides, the study focuses on the question of whether gamblers’ attitudes differ across groups with respect to the gamblers’ type of game (poker only versus poker plus other gambling activities), indebtedness, and the severity of problem gambling, with the hypothesis that such characteristics influence poker gamblers’ views on HR tools	Adult internet G recruited via ads on specialised poker dedicated forums and websites. Questionnaires completed online. Final sample 311 **online poker Gers.** Measures: demographics, G behav, game type, possible G-related debts, Problem Gambling Severity Index, perceptions of whether harm reduction measures could be helpful in reducing G-related harm		HR were endorsed to some extent varying from 25 to 53% of all participants. No HR measures were completely rejected. Time limits were less endorsed than $ limits. Automatic block when $ limit reached was considered helpful by almost 50%. Messages and warnings related to money limits were also endorsed by about 45% of the sample indicating that Internet poker gamblers consider such interactive in-game tracking and interventions as possibly helpful. **Information-related strategies were rarely endorsed by poker gamblers in comparison to more structural ones**
Mihic et al.	2022	The aim of this study was to examine cross-sectional associations of protective factors within a family and school context with adolescent risk behaviors	The study was conducted among adolescents (n = 9682) from five cities in Croatia. Mean age of participants was 16.2 years (SD = 1.2), and 52.5% were female. Multigroup structural equation modeling was used to examine relations between school attachment, school commitment, family communication, and family satisfaction with gambling, substance use, violence, and sexual risk behavior	School attachment was negatively associated with gambling and violence, while school commitment was negatively associated with students’ gambling, substance use, and violence. Gambling was also associated with family satisfaction in this model. Family protective factors were found not to be significantly related with any risk behavior	Results emphasize the importance of strengthening school protective factors, school attachment, and school commitment in preventing risk behaviors in adolescents
Moreau et al.	2020	To explore the relationships between the frequency of actual tilt episodes and the player’s perceived frequency of tilt, and the association of tilt with online poker players’ psychopathology (excessive gambling, anxiety, depression, impulsivity)	291 adult online poker players (77% controlled G prac, 13.5% moderate risk, 9.6% probable PG). Measures: Gambling Severity Index, Impulsive Behav Scale, Hospital Anx and Dep Scale, Severity of Tilting Scale, Online Poker Tilt Scale. Online survey distributed via Facebook, poker forums, email. Classification analysis to identify distinct grps of players using the tilt scales	Analysis showed sample divided into 3 grps. Grp 1: Congruent players—low perceived and measured tilt score (n = 151). Grp 2: Players underestimating tilt (n = 66) and Grp 3: Players overestimating tilt (n = 74). Grp 2 and 3 had + or—difference existing between perceived tilt freq and measured tilt freq. Signif grp diff based on excessive G scores, anxiety and dep. Over/under estimating tilt players have higher anx and dep scores with an excessive G score placing them in mod risk category. Congruent players classed as non-PG. 92% of Congruent players indicated not having tilted in last 6 mths or not a problem. Players overestimating tilt, 39% of the sample indicated being neutral or in agreement that tilt has been a problem in the last 6 mths and Players underestimating tilt’, 27% claimed to be neutral or in agreement that tile was a problem	Existence of a relation between the player’s capacity to perceive tilt and the online poker player’s behavior. Congruent grp seemed to have characteristics compatible with an appropriate level of gambling where playing was not adversely affecting a player’s life. Players underestimating tilt appear to fail to identify the emotional and behavioral changes they may experience as a tilt episode. This grp had highest tilt freq. Overestimating grp seemed to be aware of the occurrence of tilt episodes but overestimated their frequency. May confuse tilt with pathological gambling manifestations. Possible that these players experience excessive gambling symptoms that are very similar to those of a tilt episode, such as loss of control, a strong desire to recover lost bets, an inability to abstain from gambling and a strong presence of irrational beliefs. All 3 grp have relatively high impulsivity scores
Morvannou et al.	2020	To explore poker players’ perceptions and understandings of existing problem gambling prevention strategies	A secondary data analysis of interviews conducted as part of a sequential mixed-methods research project. Participants recruited from poker venues (final sample n = 12). Questions focused on themes of passion of poker, evolution of passion and poker practices, and prevention of risks linked to poker. Prevention is focus of this article	Two themes highlighted distinction between institutional and personal prevention strategies. Institutional strategies were criticised for existing formats, messages and target audience. **Institutional prevention**: a) It did not apply to me *Aware but unaffected*—aware of messaging but considered irrelevant to poker players, particularly PG (participants perceived themselves to be in control of G) *lack of knowledge and credibility behind P messages:* Messages designed for other forms of G; poker players not target audience. Lack of consideration of the distinct characteristics of poker compared to other G. *b) Lack of knowledge of player's experience—*Poker players with PG are not serious poker players and do not attempt to improve their skills, need to understand the rational aspect of poker. Money makes game addictive and risky. Overinvestment of time makes poker risky. essential to consider the characteristics of poker such as the skill component, the structural characteristics of playing against other players, and the competitive aspect of poker the game which lead them to consider it like a sport. **PERSONAL STRATEGIES** Money management (budget management, playing small amounts as a novice to poker, choosing table where other players are less experienced, and finding a balance between spending in other areas of life and on poker). Play free or low value games. Maintaining a healthy lifestyle and emotional management. Being aware of relationship with poker. Spotting paying opportunities according to players present and their skill (i.e. play against novices)	Awareness of but perceived irrelevance of messaging is problematic for existing messaging, especially if messaging addresses all type of players regardless of G form. Poker players tend not to self-identify as PG. Messaging perceived to lack understanding of poker and credibility—possibly integrate more renowned players with prevention campaigns to increase effectiveness. Players did not define their strategies are prevention but that is what they are. These strategies are perceived more positively than institutional prevention. Overall personal strategies are part of a desire to maintain control of play and improve ability and bankroll. Based on a desire to improve and enjoy poker without losing too much money. Prevention messages need to be targeted AND tailored to the poker community—ensure consistency between individual experiences and institutional prevention strategies. When developing effective prevention, there is a need to consider what strategies are employed by some players. Public Health theoretical models and conceptual frameworks of prevention have been highly focused on framing poker as an addiction that positions poker players at risk of harm—this risks isolating poker players from prevention efforts. There may be a need to rebrand existing prevention strategies and terminology. Future research should consider taking a participatory approach to prevention messaging specifically including a diversity of players who play with relatively high frequency on different forms of poker
Motka et al.	2018	To address gaps in literature by providing description of self-excluders of land and online G by focusing on (a) on their sociodemographic features, (b) the characteristics of their gambling behavior, and (c) the circumstances surrounding their acceptance of further support. (d) It analyzes their goals and motives as well as (e) barriers for self-exclusion. (f) Differences between self excluders from online and terrestrial gambling	Methodology based on proposed framework for SR by the Centre of Reviews and Dissemination. Quality assessment—Standard Quality Assessment Criteria for Evaluating Primary Research Papers from a Variety of Field. Synthesis of results of selected studies used guidelines for a narrative approach recommended by UK Economic and Social Research Council. Inclusion criteria: self-excluders, at least as a subgroup, who excluded from land and/or online G	16 studies—2 qual, 13 quant, 1 mixed-method. Online self-excluders were on average 10 years younger than land self-excluders. Self-exclusion was mainly motivated by financial problems, followed by feelings of losing control and problems with significant others. Financial problems and significant others were less important for online than for land gamblers. Main barriers for self-exclusion were complicated enrolment processes, lack of complete exclusion from all venues, little support from venue staff, and lack of adequate information on self-exclusion programs. Both self-excluders from land and online gambling had negative attitudes toward the need of professional addiction care	To exploit the full potential of self-exclusion as a measure of gambler protection, its acceptance and its utilization need to be increased by target-group-specific information addressing financial issues and the role of significant others, simplifying the administrative processes, facilitating self-exclusion at an early stage of the gambling career, offering self-determined exclusion durations, and promoting additional use of professional addiction care
Neophytou	2021	To describe the characteristics of individuals with self-reported gambling behaviours that cause problem in daily life. To identify important demographic predictors of that as-risk to develop gambling addiction. Provide preliminary descriptive data on gambling bahviours of adults in Cyprus	Random number dialing of Cyprus residents aged 18 +. 2118 participated in the phone survey. 1242 male; Mage 48 years, SD = 15. ANALYSES: Participants rated on problem gambling index. Correlations between problem gambling index and other factor were conducted. Comparison between as-risk and res of sample was conducted	Lotteries the most common form of gambling. CHARACTERISTICS OF PROBLEM BEHAVIOUR: significant effects for being male, lower reporting amusement as a reason for gambling, higher reporting escape from everyday problems as a reason for gambling. CHARACTERSTICS OF AT RISK GAMBLERS: At-risk gamblers had significantly lower income, though age and employment weren't significant	Findings consistent with previous literature regarding male gender, low income, frequency of gambling and money spent on gambling all correlate closely with at-risk gambling. Differences however were noted in age which was not significant in this sample but is often significant in other studies. Main finding is different motives contribute to at-risk groups. Gambling for amusement characterized the low risk group, while gambling to exacerbate problems characterized at-risk gamblers
Newall et al.	2022	To conduct three preregistered, incentivised online experiments on large samples of people who gamble in the UK to compare the effects of multiple versions of the “when the fun stops, stop” message	Three online experiments, in which participants could gamble real money on the results of either football matches (experiments 1 and 3) or a virtual roulette wheel (experiment 2), and in conditions designed to emulate real-life online gambling scenarios. CONSORT flowcharts provided	Of the 506 participants in experiment 1, 41·3% of available bets were made by the 254 participants in the gambling message condition, which was not significantly different (p = 0·15, odds ratio 1·22 [95% CI 0·93 to 1·61]) to the 37·8% of available bets made by the 252 participants in the control condition. In experiment 2, the only credible difference between conditions was that the 501 participants in the condition with the yellow version of the gambling message bet 3·64% (95% Bayesian credibility interval 0·00% to 7·27%) more of available funds left over than the 499 participants in the control condition. There were no credible differences between the bets made by the 500 participants in the black-and-white gambling message condition and the other conditions. In experiment 3, there were no credible differences between the 502 participants in the gambling message condition and the 501 participants in the control condition, with the largest effect being a 5·87% (95% Bayesian credibility interval –1·44% to 13·20%) increase in the probability of betting everything in the gambling message condition	No evidence was found for a protective effect of the most common UK safer gambling message "When the fun stops, stop". Any potential reductions in choice to gamble as a result of exposure to message were too small to reliably detect. Largest effect was a backfire effect which is speculated to be related to the prominence of the word "fun". Alternative interventions should be considered as part of an evidence-based Public Health approach to reducing gambling-related harm
Noel, Rosenthal and Sammartino	2022	To determine the **prevalence** of gambling activities, including sports betting, in a sample of young adults, identify **sociodemographic risk factors** for those behaviors and ascertain whether **specific gambling behaviors are associated with experiencing gambling problems**	Data from 2020 Rhode Island Young Adult Survey (RIYAS)—online survey. n = 540. Aged 18–25 yrs. Recruited via Insta ads, Facebook, direct emails to uni students. Measures: Freq of sports betting, betting on horse, trots or dogs, gaming tables at casinos or EGM at casinos. (dichotomized to past year G or G abstinence), demographics (age, gender, race/ethnicity and sexual orientation) and SES (education, employment, essential worker, subjective social status)	70% female, 60% Caucasian. 22.4% engaged in one or more G activity. The prevalence of specific gambling activities ranged from 12.4% for sports betting to 6.5% for race betting. Prevalence of any G activities was 11.5%. Odds of all gambling activities was 2 to 4 times higher among men than women. Odds of G at tables was 93% greater for Black, Indigenous, People of Color. Odds of G at casino tables and EGM was greater among older young adults. Odds of sports betting and casino G was 2 times greater among essential workers. Odds of gambling problems were 2.4 times higher among participants who engaged with sports betting (after adjusting for demo and SES variables)	Males, persons who identify as BIPOCs, older young adults and essential workers had higher odds of participating in gambling activities, although there was variation across specific activities. Sports betting was consistently associated with PG. Consistent with literature. Sports betting rate reported higher than smoking rate in USA. Substantial prevention efforts needed as sports betting availability increases. Existing gambling prevention research base focuses largely on individual harm reduction Ixs which are reliant on voluntary adherence to Ix
Nyemcsok et al.	2021	To explore young people’s perceptions about the normalisation of gambling in sport, how they critically reflect on the factors that may contribute to the normalisation of gambling, and the strategies they perceive could prevent or address the normalisation of gambling in sport. Research questions: 1. How do young people describe the relationship between gambling and sport? 2. What factors do they believe to be most influential in shaping the normalisation of gambling in sport? 3. Do young people critically reflect on the normalisation of gambling in sport? 4. Which types of strategies, if any, are needed to alert young people about the risks associated with gambling?	Analysis of qualitative data from mixed methods study of n = 111 young people 11–16 yo in Victoria who were self-reported fans of basketball. Age range selected as when become aware of brand marketing and able to understand persuasive intent of marketing. Basketball fans as AFL and NRL fans previously researched. Convenience and purposive sampling. 10 min one on one interviewer assisted survey	**Factors contributing to normalisation of G:** Two themes emerged (1) perception that G was a regular activity in which most people engaged (some perceived that "big sports fans" G most). Agreed that G was a normal part of sport because G on sport was now socially accepted or because they heard conversations about G on sport or part of family activities. (2) Role of marketing. Observed an increase in G ads. Pervasiveness of ads. marketing promotion G as a normal part of sport. Marketing provided a call to action to G. **Critical reflections about G advertising aligned with spor**t: Participants who disagreed G was normal part of sport felt it should not be something to engage in to enjoy sport—focus of sport should be watching and having fun rather than $. Others took a moral or emotive stance. Others said just because it was normal, didn't mean they agreed with it. Views about ad ranged from highlight critical to more measured. Some concerned about risk assoc with G for YP. Expressed concern about ad products that could be addictive. **Strategies to counter the risks assoc with G for YP**: Education about risks assoc w G. Current education in school was limited. Need for awareness-raising, including contact with people experiencing G harm and specialised ed about risks and consequences of G-related harm. Campaigns to warn young people about risks and to consider YP media habits and how they engage with media. Recommended reductions and restrictions in marketing. Removing ads from family-friendly channels and restricting the timing of advertising to periods when young people would be less likely to be exposed	YP are aware of G and G marketing and increased social acceptance of G. YP viewed volume and content of marketing may normalise G. Adolescence is an ideal time to intervene and develop strategies to prevent young people from developing normalised attitudes towards gambling that may increase their risk of gambling-related harm. There is good evidence about the benefits of education from other areas in Public Health, but with the caveats that it must be: (i) part of a comprehensive approach; (ii) adequately funded; (iii) sustained; (iv) research-based; (v) independent of influence from commercial interests; and vi) free to engage in forceful counter-advertising if required. Education must be approached with caution, in particular to avoid “ineffective individually targeted information and educational approaches” of the kind typically promoted by harmful industries. Some harm minimisation messaging may reinforce the behaviour it is supposed to prevent
Obedzinski et al.	2019	T complement this existing research on G behaviours, stress, coping and motivation (i.e. a psychological perspective) by incorporating a sociological perspective, which includes analysing the social and motivational forces that influence problem-gambling behaviours in university-age students. Informed by General Strain Theory and the Generality of Deviance frameworks, this study explores whether PG risk is assoc with perceived stress, G motives and other risky behaviors	DATA: Student Leisure and Well-Being Survey administered to students enrolled in sections of either an Introduction to Sociology or an Introduction to Research Methods Course at the University of Manitoba. MEASURES: PG Risk Level, motivations for G, stressful life events, alcohol-related probs, other risky behav (i.e. cigarettes, sexual behav, drugs, binge drinking). PARTICIPANTS: 283 final sample	Perceived stress is the only variable not signif at the multivariate level. All of the G motivations were signif. Coping G motivation assoc with 2.9% change in PG risk, social G motivation with 37.2% change in PG risk, financial G motivation assoc with 12.2% change in PG risk. Enhancement G motivation assoc with 25.1% change in PG risk. All risky behav signif multivariate predictors of PG	Limited support for General Strain Theory, as gambling to cope was associated with problem gambling risk, but perceived stress was not. Generality of Deviance was supported, as various ‘risky’ behaviours—unplanned or unprotected sexual relations, binge drinking, problem drinking, cigarette smoking, illegal drug use— were correlated with increased problem gambling risk
Oh et al.	2017	The aim of this review is to examine features pertinent to effective educational-based programs in the area of adolescent problem gambling prevention in hopes of providing a foundation and future suggestions for preventive eforts			Educational-based programs that adopted the unique determinant approach, which targeted risk factors to prevent PG among adolescents, have shown consistent program effect in increasing knowledge and correcting misconceptions about gambling, and consequently increase resistance towards gambling myths and fallacies. However, there is insufficient evidence from these programs to conclude that having good gambling knowledge and belief system can effectively reduce actual youth gambling behaviour
Orlowski et al.	2019	The aim of this study was to investigate the association between specific Emotional Regulation strategies and pathological as well as problematic gambling in a proactively recruited sample	A large and unselected sample (n = 4,928) has been screened proactively and systematically in vocational schools. We assessed the Affective Style Questionnaire to measure ER strategies and the Stinchfield questionnaire for assessing problematic and pathological gambling. Associations were investigated with linear and multinomial logistic regression analyses	The analyses showed a significant negative correlation between the subscales “Adjusting” and “Tolerating” and the Stinchfield sum score. Lower scores on these subscales were associated with a higher number of endorsed Stinchfield items. A lower score on the ER strategies “Adjusting” [conditional odds ratio (COR) = 0.95, confidence interval (CI) = 0.91–0.99] and “Tolerating” [COR = 0.95, CI = 0.92–99] led to a higher chance of being classified as a pathological gambler. In problematic gambling, on a subthreshold level, only “Tolerating” turned out to be significant [COR = 0.96, CI = 0.93–0.99]. D	For the first time, deficits in specific ER strategies were identified as independent risk factors for problematic and pathological gambling in a large and proactively recruited sample. ER skills, especially acceptance focused strategies, should be considered in prevention and psychotherapy
Palmer du Preez et al.	2021	The aim of this study was to identify and explore gender-related issues, notions and practices, in relation to gambling harm for women. Key questions guiding this inquiry included: What gender or gender-related issues, notions or practices were discussed in relation to gambling harm? What are the implications of gender-related issues, notions or practices for women’s experiences of gambling harm? What are the implications of gender-related issues, notions or practices for women’s gambling harm reduction?	Secondary analysis using a feminist social constructionist lens of two existing data sets related to gambling-related harm in NZ	Women’s socio-cultural positioning as primary caregivers for families and children constrained their ability to access a range of recreational and support options and increased the attractiveness of local gambling opportunities as accessible and ‘safe’ outlets for stress reduction. Patriarchal practices of power and control within family contexts operated to maintain gambling behaviour, shut down alternative recreational opportunities, and limit women’s autonomy. Consideration of these themes in relation to current health promotion practice in New Zealand revealed that national programmes and strategies appear to be operating without cognisance of these gender dynamics and therefore have the potential to exacerbate or cause some women harm	This study demonstrates the value of theoretically informed gender analysis for gambling harm reduction research, policy and practice. International guidelines for gender-aware and gender-responsive health research and practice should be engaged as a foundation for strategic and effective gambling harm reduction programmes, projects, research and policy, and as an essential part of developing and implementing interventions for gambling harm
Parham et al.	2019		The Maryland Smart Choices Program (MD-Smart Choices), a gambling prevention program for middle and high school youth. This 3-session, 45-min program was developed for implementation in Baltimore City Public Schools, an urban and predominately African American district with specific aims to engage students, encourage positive behavior, and facilitate learning related to gambling disorder. Pre–post program participation assessments were collected from 72 students across 5 different schools. Pre/post assessment to assess factual knowledge of and attitudes to gambling	Results yielded significant increases in student awareness and knowledge following participation in MD-Smart Choices. Focus group data collected from program facilitators suggested high student engagement and participation, program feasibility, and ease of implementation. No difference in student involvement in gambling activities were noted	Long term efficacy of program may be function of educational material and dispelling of myths among youth around gambling risk and skills requirements
Paterson et al.	2021	The aim of this review was to firstly understand the scope of peer-reviewed evidence on ICT-based strategies to reduce online gambling harm, and secondly, what evidence exists specifically in relation to ICT-based harm reduction initiatives for people who gamble online		Computerised interventions with personalised feedback most effective ICT intervention for sustained behaviour change	Despite the widespread potential ICT represent for addressing gambling harm there has been only limited published research to date
Peter et al.	2021	The primary aim of the present study was to test whether MI-based messages could increase rates of online self-assessment of gambling problems in comparison to a control message that did not include motivational elements	Participants were randomly assigned to 1 of 3 message conditions that all offered participants the choice to complete either a problem gambling screener or an alternative questionnaire focused on gambling-related attitudes. The first condition was an MI-based interactive message, the second was similar in content but was presented in a noninteractive manner, and the third was a control message that did not include motivational elements	t the interactive motivational message yielded significantly higher rates of screener completion (39%) than the noninteractive message (28%) or control message (29%), 2 (2, N 805) 8.28, p .016, .29. This remained significant after controlling for other study variables. Controlling for message condition, participants were more likely to complete the screener if they gambled more frequently, with more money, were more psychologically distressed and interested in receiving help for gambling problems, or had ever received treatment for gambling problems	Findings provide support for the use of interactive MI-based messages to encourage individuals at-risk for experiencing problems to use helping resources
Peter et al.	2019	The purpose of this meta-analysis was to quantify the efficacy of gambling-focused Personalised Feedback Interventions, taking into account differences in sample characteristics, content, and modes of delivery. We hypothesized that interventions would be more effective among individuals with greater gambling severity but would not vary as a function of being tested in a college versus community sample	Outcome measures only included: amount of time or money spent gambling	Sixteen interventions from 11 studies were reviewed. We found a small, statistically significant effect in favour of PFIs versus control (d = 0.20, 95% CI 0.12, 0.27). Six moderators of intervention efficacy were explored. These interventions appeared to be most efficacious when used in populations of greater gambling severity, when individuals were provided with gambling-related educational information, and when used in conjunction with motivational interviewing. Factors associated with reduced efcacy include in-person delivery of feedback without motivational-interviewing and informing participants of their score on a psychological measure of gambling severity. Efcacy did not vary as a function of college or community samples	PFIs are a low cost, easily disseminated intervention that can be used as a harm-reduction strategy. However, more substantial effects may be attained if used as part of a larger course of therapy
Peterson et al.	2021	To provide an overview of the literature on Protective Behavioural Strategies measures for various risk behaviours, and common interventions used in conjunction with PBS		Of the articles reviewed, 15 validated PBS measures were found and eight distinct categories of PBS interventions. The 15 measures reviewed included risk factors such as alcohol use/consequences (n = 8), dating and sexual behaviors (n = 4), gambling (n = 1), cannabis (n = 1), and condom use (n = 1). A survey of the literature produced eight distinct categories of interventions with varying degrees of effectiveness: (a) Brief Motivational Interventions, (b) Personalized Normative Feedback, (c) PBS Skills Training, (d) PBS Instruction, (e) Deviance Regulation Theory Interventions, (f) Behavioral Economic Based Interventions, (g) Counterfactual Thinking and (h) Episodic Future Thinking	Findings from the present study corroborate the notion that PBS effectively reduce negative consequences associated with behaviors, such as negative alcohol-related consequences, harmful cannabis use, and adverse sexual outcomes. Research on interventions targeting PBS is lacking in areas outside of alcohol use. Within alcohol use, the utility of interventions varies widely. Understanding the reason for this discrepancy is an important area for future research
Piarska et al.	2020	To identify psychosocial and behavioural factors associated with gambling involvement among 16- to 18-year-old adolescents	The sample includes 511 adolescents (57.5% of men), who participated in a longitudinal study. Classrooms were randomly selected from public/non-public general, technical high schools and basic vocational schools from Warsaw. The self-administered anonymous questionnaire was completed during school lessons. Data reported in this study were from wave 1 (10th grade) and wave 2 (12th grade) with a response rate of about 65%. Gambling involvement was measured by combining a measure of six types of gambling behaviours and gambling-related problems in the last year. Gambling-related problems were measured by the Polish adaptation of the South Oaks Gambling Screen Revised for Adolescents. Both protective and risk factors measured in the study were selected from four broad domains representing (1) individual characteristic, (2) peer, (3) parental/familial and (4) school influences	About 50% of students were involved in some form of gambling at least once in their lifetime. The most prevalent forms of gambling among study participants included lottery games, scratch cards, card games and participation in sports betting. Approximately 34% of adolescents have had symptoms of increased gambling involvement. Generalised linear model analysis showed that male gender, wave 1 gambling, sensation seeking, delinquency and cyberbullying were the risk factors. Positive relationships with parents and meaningful activities were found as protective factors against gambling involvement	Findings indicate that prevention programs/interventions based on positive parent–child relation building and meaningful activities allowing satisfaction of the need to take risks in a socially acceptable manner can be effective in counteracting increased gambling involvement among youth
Pitt et al.	2017	To explore children’s gambling attitudes and consumption intentions and the range of consumer socialisation factors that may influence these attitudes and behaviours	Children aged 8 to 16 years old (n = 48) were interviewed in Melbourne, Australia. A semi-structured interview format included activities with children and open-ended questions. We explored children’s perceptions of the popularity of different gambling products, their current engagement with gambling, and their future gambling consumption intentions. We used thematic analysis to explore children’s narratives with a focus on the range of socialising factors that may shape children’s gambling attitudes and perceptions	Three key themes emerged from the data. First, children’s perceptions of the popularity of different products were shaped by what they had seen or heard about these products, whether through family activities, the media (and in particular marketing) of gambling products, and/or the alignment of gambling products with sport. Second, children’s gambling behaviours were influenced by family members and culturally valued events. Third, many children indicated consumption intentions towards sports betting. This was due to four key factors: (1) the alignment of gambling with culturally valued activities; (2) their perceived knowledge about sport; (3) the marketing and advertising of gambling products (and in particular sports betting); and (4) the influence of friends and family	This study indicates that there is a range of socialisation factors, particularly family and the media (predominantly via marketing), which may be positively shaping children’s gambling attitudes, behaviours and consumption intentions. There is a need for governments to develop effective policies and regulations to reduce children’s exposure to gambling products and ensure they are protected from the harms associated with gambling
Pitt et al.	2022	To explore older adults’ receptivity to, knowledge of, and engagement with EGMs	20 focus groups were conducted in Melbourne with n = 126 adults aged 55 + , who had attended a club or pub in the last 12 months. Topics included EGM attitudes and behaviours, structural characteristics of EGMs, and the potential risks associated with EGM gambling. The study utilised a co-production approach with seven Local Government Authorities—representatives gave feedback at each stage of the research process	MOTIVATIONS FOR ENGAGING IN EGM G: recreational and incidental to other activities in venue (i.e. social gatherings, attending organised activities within venue). Only engaged in EGM gambling because EGMs were within the same venue, were available and accessible, and were tempting to play. Bingo created a path to EGM G (due to co-location). A few participants indicated that they perceived some activities were offered to encourage G on EGMs. A few commented that they went to venue specifically to G. Food and drinks and vouchers and rewards encouraged G	
Pitt et al.	2021	To address evidence gap around specific experiences of older people motivation for engaging in EGMs, their knowledge about the structural characteristics of EGMs, and their perceptions of personal risk of developing harm from EGMs	Focus groups were conducted in Melbourne, Australia with n = 126 adults aged 55 + , who had attended a club or pub in the last 12 months. Topics included EGM attitudes and behaviours, structural characteristics of EGMs, and the potential risks associated with EGM gambling. Thematic analysis was used to interpret the data	For most participants, EGM gambling was secondary to their participation in other activities available within venues. Participants identified structural characteristics of EGMs; however, there were some misconceptions about how EGMs operated, including how or why machines paid out. Most participants perceived that they were not at risk of gambling harm because they engaged in “responsible” gambling practices such as setting limits	Older adults often engaged in EGM gambling because of its availability in community-based venues. Older adults’ perception that they are implementing responsible gambling practices may be increasing their susceptibility to harm
Pitt et al.	2021	To understand the factors that may influence how and why people with intellectual disability may engage in gambling	Nineteen people with intellectual disability were recruited from a disability advocacy organization and participated in face to face, semi-structured qualitative interviews. Open ended questions were used to explore participants’ gambling participation, recall of, and attitudes toward, different gambling products, understanding of gambling harm, and awareness of responsible gambling messages	All participants could remember gambling in their lifetime and some participants had recently engaged in gambling. Many participants were aware of different gambling products, and a few participants could describe in detail the technical aspects of electronic gambling machines. Most participants did not specifically recall seeing gambling harm minimization messages, however some described engaging in individual responsibility measures, such as limits and control, as they perceived this reduced the risks of experiencing harm	People with intellectual disability are engaging with gambling products in a similar way to the general community. Therefore, it is important to understand the different pathways that may lead people with intellectual disability to initiate and continue gambling and to ensure that they are aware of and protected from the potential risk. Policy makers and practitioners should seek to understand and implement a range of strategies to reduce and prevent the harms associated with particular gambling products and environments for this population sub-group
Pitt et al.	2022	To document young people's perceptions about strategies that could be used to counter the normalisation of gambling and prevent gambling-related harm	Qualitative interviews with a constructivist approach. Data from a broader study investigating the normalisation of gambling for young people in Australia. Young people aged 11–17 invited to participate through convenience, purposive, and snowball techniques. Semi-structured interviews of ~ 1 h. For this study, asked to reflect on strategies the could counter the normalisation of gambling or reduce gambling-related harm. 54 young people (25 girls, 29 boys)	Five themes constructed: Reducing the accessibility and availability of gambling products; Changing gambling infrastructure to help reduce the risks associated with product engagement; Untangling the relationship between gambling and sport; Restrictions on advertising.; The need for counter-framing in commercial messages about gambling	The five themes identify key strategies suggested by young people to counter normalisation of gambling and reduce gambling-related harm. Strategies recommended are similar to de-normalisation and harm prevention strategies that have been endorsed by Public Health experts, key stakeholders, and those with lived experiences of gambling harm. Most research with young people has focussed on factors that contribute to normalisation, this study provides suggestions to combat this, showing they're able to contextualise and suggest strategies which may address this
Rafi et al.	2019	To explore the experiences of middle management in WHPPs in relation to PG	Semi-structured interviews to examine experiences of WHPP training and skills development in policy implementation arms of program	The qualitative content analysis resulted in six themes: (1) Expectations of the skills-development training, (2) Experiences of and prior beliefs about problem gambling, (3) A good foundation, (4) The difficult conversation, (5) Appreciated aspects of the training sessions, and (6) Remaining obstacles. The results suggest that the presentation of cases, facts, and general knowledge was appreciated by most participants. However, participants also expressed that they would benefit from tailored interventions, more support in the policy implementation process, and following up on the results	
Ranta et al.	2018	To examine the effect, over 36 months, of a brief problem gambling intervention on depression in a population of people seeking help for gambling issues	One-hundred and thirty-one participants were recruited from adult (18 + years) gambler callers to the New Zealand national gambling helpline. They received a manualised version of the helpline’s brief intervention, and were assessed at baseline, 12 and 36 months	Overall, problem gambling severity reduced from a score of 17 (using the Problem Gambling Severity Index) at baseline to a score of 7.5 at 36 months. The percentage of participants with depression reduced from 74% at baseline to 41% at 36 months. For both problem gambling and depression, the greatest reduction was in the first 12 months. Multiple logistic regression analyses at baseline showed an association between problem gambling and depression. Repeated measures logistic regression indicated that reduced problem gambling severity reduced depression and that there was no independent time efect taking place (i.e. the decreased depression was not due to natural recovery)	Thus a single brief telephone intervention for problem gambling substantially reduced the prevalence of depression. This has clinical and Public Health implications with a benefit being that people with depression and co-existing gambling problems may not necessarily need additional treatment for depression if they receive treatment for their gambling issues
Rash et al.	2018	To examine motives for not gambling among a diverse sample of adult lifetime non-gamblers recruited from the community and to compare these motives to an undergraduate student sample of non-gamblers from a previous study	Participants consisted of 219 lifetime non-gamblers (45.2% male) from the United States recruited via Amazon's Mechanical Turk. The previously recruited sample consisted of Canadian undergraduate students (n = 196)	Eight distinct categories of motivations for not gambling were identified in the sample of adult community non-gamblers, which corresponded closely with previous findings from the student sample. However, comparisons between the two samples revealed that adult lifetime non-gamblers were more likely to provide financial motives as reasons for not gambling. Whereas, the student sample was more likely to mention disinterest and the influence of others as reasons to avoid gambling	The choice not to gamble among lifetime non-gamblers may reflect a more conscious, values-based decision when compared to undergraduate non-gamblers
Raybould et al.	2021	Determine whether there are differences in the presentation of gambling harms across demographic groups or gambling behaviour categories	Systematic Review. PRISMA-P. Inclusion criteria: Focus on gambling harms; Focus on harms to the gambler rather than others; Discussion of specific harms, not just general	59 studies between 1994 and 2020 were included. Replicated differences were found in groups defined by age (younger more at risk of dependence/problem gambling and social harms), socioeconomic status (less affluent more at risk of harms), education level (less educated more at risk of harm), ethnicity and culture (family gambling, migrants more at risk), risk severity (harms increase with risk of developing problem gambling), and gambling behaviours (high frequency = more harm. low frequency = mild harm such as shame and guilt. Years of gambling associated with thoughts of self-harm)	Harms appear to be dependent on specific social, demographic and environmental conditions, suggesting a health inequality in gambling-related harms. More information on confounders is still required
Ren et al.	2019	Assess long-term effectiveness of a school-based youth gambling prevention program in Illinois	Pre- and post-test on knowledge of gambling for all students. 5th grade and above received a post-test gambling screen (Modified South Oaks Gambling Screen for Teens (MSOGST). 16,262 completed pre & post tests, 16,421 completed MSOGST. 76.1% high school students, approx half female, and 21.3% received the intervention twice or more	Students receiving multiple interventions had higher pre-test scores. Those who had the intervention twice were less likely to be problem gamblers than those who had it once. Overall, improvements in post-test scores were reported compared to pre-test scores. Males more likely to be pathological gamblers than females	Pre-test scores increase across years, due to more students receiving multiple interventions. Pre-test scores for first timers did not change however, so no peer-education for untrained youth was occurring. Long term positive effect for DGAOF program increasing gambling knowledge and possibly reducing rates of pathological gambling. Multiple repeated measures are important
Ridder and Deighton	2022	The primary aim of the present study was to examine the relationships between problem gambling behaviour, shame (as both a personality trait and an emotional state), gambling-related self-efficacy, and dysfunctional coping mechanisms	The sample consisted of 235 participants (172 male; mean age = 30.32, SD = 8.57), who completed an online, self-report questionnaire that assessed gambling severity, shame-proneness, post-gambling shame and guilt, and dysfunctional coping. Data were assessed using path analysis models	More problematic gambling was significantly associated with dysfunctional coping. A pathway mediation model was determined and several mediator variables were found to operate in series, including: shame proneness, post-gambling shame, and gambling-related self-efficacy	This research supports and builds on previous research that has highlighted the detrimental effect of problem gambling and shame on coping mechanisms, and the benefits of gambling-related self-efficacy for subsequent coping. A limitation of the study was that 18.7% of the sample reported scores indicative of probable pathological gambling, while most of the remaining sample were representative of recreational gambling
Rider et al.	2018	Compare the prevalence of gambling behaviours and problem gambling among transgender and gender diverse (TDG) youth vs cisgender adolescents	Recruited from the 2016 Minnesota Student Survey, statewide surveillance effort. 80,929 students, 2168 (2.7%) were TGD in 9th and 11th grades (only grades asked about gender identity). Gambling behaviour, problem gambling both assessed	TGD youth assigned male at birth were equally likely as is gender assigned males to be involved in gambling behavoiurs. All youth assigned male at bith regardless of identity were significantly more likely to report gambling behaviours than females. TDG youth assigned male at birth were at greater risk of screening positive for problem gambling than other groups	Involvement in gambling over the past year was roughly equivalent between TGD and cis groups, though type of games differed. TGD youth were at greater risk of problem gambling than cis youth, especially those assigned male at birth. This warrants further exploration
Rodda	2021	Review to determine the attitudes and preferences of gamblers and their families on systems or tools to restrict access to money and cash, as well as their effectiveness	Systematic search of articles relating to financial restrictions and gambling. Eligibility included samples of gamblers and interventions tarted at money or cash restrictions in gambling contexts. Soft financial barriers (i.e. family involvement) were explored, as were limit setting systems focused on limiting expenditure within venues	9 studies met eligibility. Three focussed on financial systems (e.g. ban on credit betting), six on removal of cash machines from gambling venues. Included studies were generally of low quality	The included studies provided support for financial mechanisms to support gamblers and their families. Future studies need to involve multiple stakeholders to provide this time of support, as well as evaluation of the holistic impact that hard barriers can have on gambling and gambling-related harm
Rodda et al.	2020	The aim of the current feasibility study was to investigate whether an action and coping planning intervention could (1) be deployed within a gambling venue, (2) increase adherence to goal intentions regarding gambling spend during the gambling session (primary outcome), and (3) maintain adherence to intentions at 30-days post-intervention (secondary outcome). A secondary aim was to examine the effectiveness of the intervention by the level of gambling severity	PGSI pre/post; Readiness to implement strategies; expenditure: intent to spend; actual spend; anticipates spend on pokies over next 30 days; and Timeline follow back: spend 30 days prior and 30 days post intervention	No difference across groups in terms of intended spend and PGSI scores All groups spent less or similar to their intended limit with fewer MR/PG sticking to limits. However, no effect of intervention on intended spend Actual spend was substantially lower in intervention group than control within the venue substantially lower Intended spend for 30 post intervention was noted in intervention group cf control PGSI scores were negatively correlated to readiness to implement. Higher PGSI scores were also positively correlated to an 'in-venue strategy' Predictors of sticking to limits over 30 days post intervention were amount intended to spend, and negative for intention to use a strategy and PGSI scores	A simple brief intervention appears feasible in gambling venues and have an impact on gambling intentions over the short term
Rodda et al.	2018	To develop a reliable classification system capable of identifying PSYCHOLOGICAL Ix characteristics that could, potentially, account for greater or lesser effectiveness in reducing G or PG	SRs examining the efficacy of psychological interventions for problem gambling were used to identify studies for inclusion Intervention descriptions were content analyzed to identify common and differentiating characteristics (1980–2016, valuated the effectiveness of a psychological intervention for the treatment of gambling problems using a randomized controlled trial (RCT), randomized trial, quasi-randomized trial, or cross-over RCT study design, included outcome measure). 46 psychological and self-help gambling interventions reviewed. Development of classification system: generated four broad sets of categories: (a) types of change technique used in interventions (e.g., cognitive restructuring), (b) participant and study characteristics (e.g., recruitment), (c) characteristics of the delivery and conduct of the intervention (e.g., modality of delivery), and (d) evaluation characteristics (e.g., type of control group)	CHANGE TECHNIQUES: Behaviour substitution, cognitive restructuring, decisional balance, feedback on assessment, financial regulation, goal setting, exposure, imaginal desensitization, information gathering, information provision, motivational enhancement, problem solving, relapse prevention, self-monitoring, social comparison, social skills training, plan social support, stimulus control. 88 Ix descriptions extracted from 46 studies. categories of change-technique types identified in this study overlapped with those identified previously in analyses of frequently employed techniques to change behavior, in general. A subsequent refined list of categories especially relevant to alcohol-use reduction include self-monitoring, relapse prevention, social comparison, goal setting, motivational enhancement, and social support. Relapse prevention was used in 60% of interventions, cognitive restructuring in 52%, and behavioral substitution in 44%. GAPS: Limited research on use of third wave therapies (e.g. acceptance and commitment therapy), just in time Ix and cognitive-neurological Ix. Most Ix delivered F2F	Demonstrates the feasibility of a common language for all psychological interventions for PG regardless of mode of delivery or degree of therapist involvement. It provides a model for consistency in reporting of G Ix and it also identifies areas where current reporting is inconsistent or absent. Furthermore, by identifying what currently happens in psychological interventions for problem gambling, gaps have been identified and potential new types of interventions can be pursued
Rodda et al.	2021	Review and critically assess the literature related to the effectiveness of internet delivered prevention, harm reduction, and early intervention for gambling problems	Systematic Review. PRISMA. Terms relating to gambling AND internet-based intervention AND intervention type. Must be longitudinal, include at-risk or problem gamblers, focus on prevention harm or early intervention, involve delivery of content (not just a tool or resource), delivered online, include outcome measure on frequency or severity	15 studies were included. 1 study on prevention, 7 on harm reduction, 7 on early intervention. 8 were RCT. 8 target group accessing bettering and casino websites. Four types of interventions; personalised and normative feedback, limit setting, self-directed CBT, self-exclusion	The available literature shows promise in the effectiveness of internet delivered interventions. The limited number of studies (compared to 55 in a broader umbrella review of interventions) suggests internet delivered interventions could be leveraged more
Roderique-Davies et al.	2020	Pilot. Investigate whether exposure to embedded gambling promotions during televised football elicit urges to gamble amongst students. Explore whether severity of reported gambling varied between those who study sports-related and non-sports subjects	30 sports student and 30 non-sports students (overall 58.3% male). 10 in each video group. Gambling Urge Scale (GUS), Problem Gambling Severity Index (PGSI) for assessment. Data collected post-video only	The sports students had significantly higher mean PGSI scores and GUS scores. Significantly differences in GUS were noted between the 3 video groups, group (a) with highest urge, group (c) with lowest urge. The sports group had significantly higher urge from video (b) compared to (c), while the non-sports group had equal urges on both	Sports students exposed to televised sport with embedded gambling reported highest gambling urges. It was observed amongst non-sport students but at a lesser extent. Amateur sport with no cues elicited some gambling urge from sports students but none from other students
Russell et al.	2019	To determine whether demographic, behavioural, and psychological risk factors (previously identified among sports bettors) are specifically related to sports bettors rather than gamblers in general	15,225 respondents, only 1147 for analyses. Of 1147, 66.5% male, mean age 41.17 (SD = 14.5, all 18 +). Sports Betting Problem Gambling Severity Index (SB-PGSI) to measure gambling severity	Risk factors for moderate risk to problematic sports bettor were (bivariate results): Being younger; speaking a language other than English at home, being single, having higher income, having higher disposable impact; betting more frequently, betting on a higher number of other mediums; higher proportion of overseas bettering, higher proportion of telephone betting, NOT engaging in fantasy sports, higher gambling urges. Gender was not significant, though p = .053. Multivariate models left only higher monthly sports betting expenditure, higher gambling urges, alcohol issues, and self control	Results suggest that sports betting itself (rather than co-occurring gambling on other forms) contributes to gambling problems and harms. Distal effects such as age and disposable income were not significant when proximal factors (i.e. gambling urges and beliefs) were included in multivariate analyses. Distal risk factors can still be used to inform the targeting of interventions
Salonen et al.	2018	To measure gambling expenditure by game type while controlling for demographics and other gambling participation factors. A further aim was to find out how each game type was associated with gambling expenditure when the number of game types played is adjusted for	Using data from the 2015 Finnish Gambling survey on adult gamblers (n = 3555), multiple log-linear regression was used to examine the effects of demographics, gambling participation, and engaging in different game types on weekly gambling expenditure (WGE) and relative gambling expenditure (RGE)	Male gender, lower education level, higher gambling frequency and higher number of game types increased both WGE and RGE, while younger age decreased WGE but increased RGE. Furthermore, seven specific game types increased both WGE and RGE. Weekly horse race betting and non-monopoly gambling had the strongest increasing effect on expenditure. Betting games and online poker were associated with higher expenditure even when they were played less often than weekly. Among weekly gamblers the highest mean WGE was recorded for those who played non-monopoly games (146.84 €/week), online poker (59.61 €/week), scratch games (51.77 €/week) and horse race betting (48.67 €/week). Those who played only 1–2 game types a week had the highest mean WGE and RGE on horse race betting and other betting games	It seems that overall gambling frequency is the strongest indicator of high gambling expenditure. Our results showed that different game types had different effect sizes on gambling expenditure. Weekly gambling on horse races and non-monopoly games had the greatest increasing effect on expenditure. However, different game types also varied based on their popularity. The extent of potential harms caused by high expenditure therefore also varies on the population level. Based on our results, future prevention and harm minimization efforts should be tailored to different game types for greater effectiveness
Saunders et al.	2021	The aim of this review is to identify broad key principles which underpin appropriate problem gambling interventions within the literature, both theoretical and empirical, relating to the indigenous communities of Canada, Australia, New Zealand, and U.S.A		Significant lack of empirical evidence as to interventions but clear theoretical themes from literature: - Adopting a culturally-centred approach to intervene on Indigenous populations - Must have an Indigenist foundation: elder-led models, locally specific, inclusion of indigenous staff in services - Community-controlled across the life-span of the intervention - Capacity strengthening of the community - Holistic approach - Ensuring a competent workforce - A collaborative network - Regulation as a support	Mostly conceptual information that makes it impossible to adequately inform program and policy design. Given overrepresentation of Indigenous people in relation to gambling harm, this is of significant concern
Savolainen et al.	2019	To examine how social identification with online and offline peer groups associates with youth problem gambling behavior and if perceived social support buffers this relationship	Data were gathered with an online survey with 1212 American and 1200 Finnish participants between 15 and 25 years of age Measures included the South Oaks Gambling Screen for problem gambling, and items for peer group identification and perceived social support		Online peer groups may be a determinant in youth problem gambling. Focusing on offline peer groups and increasing social support can hold significant potential in youth gambling prevention
Savolainen et al.	2020	Employing the cognitive discrepancy loneliness model, this study aimed to provide a social psychological perspective on youth addictions	A comprehensive survey was used to collect data from American (N = 1212; mean 20.05, SD 3.19; 608/1212, 50.17% women), South Korean (N = 1192; mean 20.61, SD 3.24; 601/1192, 50.42% women), and Finnish (N = 1200; mean 21.29, SD 2.85; 600/1200, 50.00% women) youths aged 15 to 25 years. Perceived loneliness was assessed with the 3-item Loneliness Scale. A total of 3 addictive behaviors were measured, including excessive alcohol use, compulsive internet use, and problem gambling. A total of 2 separate models using linear regression analyses were estimated for each country to examine the association between perceived loneliness and addiction	Loneliness was significantly related to only compulsive internet use among the youth in all 3 countries (P < .001 in the United States, South Korea, and Finland). In the South Korean sample, the association remained significant with excessive alcohol use (P < .001) and problem gambling (P < .001), even after controlling for potentially confounding psychological variables	The findings reveal existing differences between youths who spend excessive amounts of time online and those who engage in other types of addictive behaviors. Experiencing loneliness is consistently linked to compulsive internet use across countries, although different underlying factors may explain other forms of addiction. These findings provide a deeper understanding in the mechanisms of youth addiction and can help improve prevention and intervention work, especially in terms of compulsive internet use
Saxton et al.	2021	1. To examine the efficacy of ‘pure PNF’ interventions (i.e., no other intervention implemented) for hazardous alcohol use, problem gambling, illicit drug and tobacco use, relative to passive control groups, in reducing frequency of use and symptom severity 2. To examine the efficacy of PNF plus self-directed interventions (‘mixed PNF interventions’) for hazardous alcohol use, problem gambling, illicit drug and tobacco use, relative to passive control groups, in reducing frequency of use and symptom severity 3. To examine whether addictive disorder type, setting (e.g., university environment), and type of additional intervention components explain the variability in the magnitude of the PNF intervention effects 4. To examine the extent to which methodological risk of bias characteristics influence PNF intervention effects		PNF alone, and with additional interventions, reduced short-term alcohol frequency and symptom severity. PNF with additional interventions reduced short-term gambling symptom severity. Effect sizes were small. PNF did not alter illicit drug use	The limited number of studies suggest further research is needed to ascertain the efficacy of PNF for gambling and illicit drug use
Schulte et al.	2021	To investigate the predictive ability and the interplay of two risk factors for trajectories of GD: the psychopathological risk factor of gambling-related cognitive distortions and the sociodemographic risk factor of having an Migrant Background (MB)	A longitudinal design is used, assessing gambling severity and gambling-related cognitive distortions at baseline and 10 months apart in a young adult sample with a focus on individuals with MB. Both, cognitive distortions and MB as risk factors for GD were hypothesized to be associated with an increase of fulfilled DSM-5 criteria for GD over time Measures: assessment of gambling severity; assessment of gambling frequency; assessment of cognitive distortions	This study investigates in a young adult sample (N = 268, age range 16–30 years) changes of gambling related cognitive distortions and Gambling Disorder severity within 10 months. The sample was proactively recruited in vocational schools in Germany. In telephone interviews, gambling-related variables were assessed. We found no differences of cognitive distortions with respect to migration background. In a GEE analysis, migration background and the believe in luck and perseverance were significantly associated with an increase of fulfilled DSM-5 criteria for Gambling Disorder over time	Findings validate the role of gambling-related cognitive distortions in this high-risk population and call for early prevention programs in the form of cognitive modification trainings specifically targeting the believe in luck and perseverance. Low-threshold prevention programs could be implemented in schools as they already exist for the prevention of alcohol abuse
Seal et al.	2022	To study the determinants of: (i) gambling behaviour, including if a person does gamble and the type of gambling engaged with; (ii) the number of sports and non-sports bets made over a 12-month period; and (iii) attitudes towards betting on sports		The probability of betting on sports decreased with increasing age and was lower for women and people with a university education. This gender difference varied with age, with the greatest difference found among the young. Similar effects were observed for the number of sports bets made, which declined with age. The gender difference in the number of sports bets also varied with age with the greatest difference found among the young arising from the high propensity of young men to bet on sports. Attitudes to sports betting were also analysed, with a key finding that, within friendship circles, the views that sports betting is perceived as harmless, common and very much a part of enjoying sports were stronger among young men. These permissive attitudes were stronger among people who bet on sports and those who bet on sports more frequently	The analysis of sports fans provides insights into the characteristics of the target market most likely to bet on sports, which can be used to inform Public Health initiatives and harm reduction campaigns
Sirola et al.	2021	Summarizes research of online gambling and monetary gaming communities based on a systematic literature review		According to results, online communities serve different functions in gambling and gaming behaviors. Gambling communities are typically forums for discussing and sharing gambling experiences, strategies, and tips as well as gambling problems, while gaming communities are inherently embedded inside a game being an essential part of the gaming experience. Identification with virtual communities influences gambling behavior and monetary gaming behavior through mechanisms of perceived norms, social influence, and community feedback. Whereas some gambling communities may provide protection from excessive gambling habits, gaming communities seem to solely motivate gaming behavior and purchase intentions	Online communities important for formation of identity, same is true for gaming/gambling. In context of positive reinforcement for gambling and in-game purchasing, behaviours is likely to change
Skinner et al.	2018	PURPOSE OF ARTICLE: Identifies, based on the knowledge and evidence currently available, Best Practices for treating gambling problems among older adults intended for practitioners, patients, families, policy makers and others concerned with this population	Based on approach in Turner et al.. 2018	These guidelines focus on five areas: (1) person-centred and family-focused care, (2) screening and assessment, (3) secondary prevention and early intervention, (4) tertiary prevention and specialized treatment, and (5) ongoing support and recovery resources	Lots of detail about the best practice guidelines within each of the five focus areas are provided in the article. Too much to present here
Stanmyre et al.	2022	(1) To investigate the five facets of mindfulness and nonattachment in an online sample of individuals who gamble; (2) to identify subgroups of individuals who gamble based on facets of mindfulness and nonattachment; and (3) to explore descriptive differences across mindfulness profiles relative to etiological precursors to problem gambling to further inform the resulting profiles	An online convenience sample of 843 adults (59.91% male; Mage = 39.40 years; SD = 12.52) who gamble completed measures of mindfulness; nonattachment; gambling motivations, cognitions, frequency, and problem severity; and mental well/ill-being	Findings from a series of latent profile analyses supported a four-profile model, representing High, Moderate, and Low Mindfulness as well as a unique profile, Judgmentally Unaware, characterized by low levels of non-judging and acting with awareness. Individuals with the Judgmentally Unaware profile demonstrated more frequent gambling, stronger gambling motivations and gambling-related cognitions, more severe problem gambling, and poorer mental health	In contrast to prior studies, this study failed to find a Nonjudgmentally Aware profile, characterized by low levels of observing but high levels of non-judgment and acting with awareness, nor the Judgmentally Observing profile, which is high in observing but low in non-judging and acting with awareness. Rather, the most clinically significant profile to emerge in the current study is the Judgmentally Unaware profile, characterized by low scores for both non-judgment and acting with awareness. In a gambling environment, individuals with lower levels of awareness may be prone to attribute wins to luck, superstition, or a winning system, and to misperceive the nature of randomness in the face of mounting losses. They may also become more easily absorbed in the gambling activity as a way to cope with stress or escape aversive moods. it is possible that judgmentalism directed at themselves may engender shame and negativity, or foster anxiety and depression, for which gambling is a welcome outlet as a stress-coping mechanism; the need for escape, coupled with the penchant for cognitive distortions, may fuel excessive play and amplify conditioning effects that lead to gambling problems. PROTECTIVE FACTOR: High mindfulness across all facets was identified as a protective factor. Those with high mindfulness may be better able to maintain control by avoiding dissociation that leads to over-expenditures of time and money and the development of erroneous perceptions about luck and winning that can lead to adverse consequences
St-Pierre et al.	2017	To assess the efficacy of targeting Negative Anticipated Emotions (NAEs) and key TPB constructs in a prevention video for modifying gambling beliefs, intentions, and behaviours in a universal adolescent G prevention tool	PARTICIPANTS: 387 adol in Gr 9, 10 and 11 randomly assigned to either an intervention (video-based preventive Ix) or a control condition. 280 participants completed measures at all time-points—pre-intervention, post-intervention, and 3-month follow-up. MEASURES: G Attitudes Scale, G Injunctive Norms Scale, NAE for G, G Intention Scale, G Activities Questionnaire—Adapted. PROCEDURE: Pre-test. Ix group watched 25-min video while control grp did regular activities. 1 week later Ix grp participated in a booster discussion session for 20–25 min while control grp did regular activities. Post-test survey. FU survey at 3 mths post-Ix	Video was not effective in producing desired changes in NAEs, the key constructs of the TPB, or the frequency of gambling behaviour. Specifically: mixed model analyses revealed no significant group-by-time interactions for gambling-related attitudes, subjective norms, perceptions of behavioural control (PBCs), NAEs, or intentions, indicating that changes from pre-intervention to follow-up were not statistically different between the intervention and the control group. Additionally, no significant group-by-time interaction was observed for frequency of gambling behaviour	Adolescents in both the intervention group and the control group reported small but significant increases in positive gambling attitudes and positive peer and family subjective norms at post-intervention and at follow-up. Although adolescents reported a slight but significant decrease in PBCs over their ability to refuse gambling at post-intervention, as well as small but significant decreases in NAEs and frequency of gambling behaviour at follow-up, no significant group differences were found
Subramaniam et al.	2017	The current study is part of a larger qualitative study exploring gambling initiation, maintenance, harm, help seeking and barriers to care among older adults in Singapore. Article describes the concept of RG among older adults (aged 60 years and above) in Singapore and reports on the cognitive and behavioural strategies employed by them to regulate their gambling	PARTICIPANTS: 25 older adults (60 yr +) who reported gambling regularly (at least weekly) currently, or those who were regular gamblers in the past METHOD: Participants self-administered South-Oaks G Screen prior to interview. In-depth interviews were used to gain an understanding of gambling from those older adults who gambled. In these narratives, research participants described their lived experience of gambling starting from the very first time they gambled, the progression over time and the consequences across the lifespan of the behaviour. This approach, which did not use specific pre-determined questions on RG, yielded a rich description of what older adults perceived as RG without biasing them in any way	Thematic analysis lead to identification of RG in 2 organising themes: (1) self-developed strategies to limit G-related harm and (2) family Ix to reduce G harm. SELF: (a) delayed gratification, perception of futility of gambling, setting limits, maintaining balance, help-seeking, abstinence, awareness of disordered G in others or in self. FAMILY: (a) pleading or threatening, (b) compel help-seeking, (c) family exclusion orders	Respondents did not use term RG but described self-developed strategies to limit G to safe limits. Limit setting, monitoring themselves and reapplying themselves to self-imposed limits if there is a discrepancy seen in RG are all essential components of self-regulation. RG demonstrates regaining or strengthening of self-regulation to prevent or regulate disordered gambling. The concept of balance identified by the older adult gamblers is somewhat unique to this study. Themes are similar to quant and qual studies conducted elsewhere, in particular to Thomas et al.. Australian study using a phenomenological framework. Study suggests that family members, by constantly engaging the respondent and setting firm limits, were frequently able to convince the respondent to gamble more responsibly. This contrasts Dowling's study suggesting nagging, disapproval, threats and ultimatums were ineffective
Tabri et al.	2017	The purpose of the current research was to assess a possible boundary condition for the known indirect effect of relative deprivation on disordered gambling (via delay discounting)	online survey of current gamblers Measures included: relative deprivation; delay discounting; financially focused self-concept; PGSI	Relative deprivation predicted greater delay discounting but not disordered gambling. Disordered gambling predicted by delayed discounting AND financially focused self-concept	relative deprivation not primary indicator of gambling severity. FFS strongly associated with increased gambling severity
Tomei et al.	2021	(1) Determine whether individuals with addictive disorders reported lower levels of prosocialness than peers without addiction. (2)Determine whether levels of prosocialness varied according to addiction type. (3)Determine whether levels of prosocialness among individuals with addictive disorders varied according to number of addictions	Young Swiss men attending army recruitment centres were invited to participate. Final sample 5654. Alcohol disorder, nicotine dependence, cannabis use, gambling disorder, gaming disorder were explored. Prosocialness measured using prosocialness scale for adults (PSA)	Gaming disorder 10.1%. Gambling disorder 1.1%. Lowest prosocialness score for gambling disorder. Next lowest gaming disorder (significantly lower than other disoders/no disorder, but significantly higher than gambling disorder)	Prosocial behaviour were lowest in gambling and gaming disorder individuals, even amongst other individuals with addictive disorders. Suggests prosocialness could be used as an awareness-raising element for young men and their peers. Also potential basis for rehabilitation programs, and as a buffer against relapse
Tong et al.	2018	(1) Measure perceived accessibility of treatment information among Macao gamblers to understand existing social demand for this information. Explored it's association with gamblers responsible gambling practices. (2) Investigate how responsible gambling (measures and policies) is perceived, and how the perception relates to responsible gambling practices	(1) 1020 respondents, mean age 44.49. 50.1% had gambling experience. 27.84% had gambled in previous 12 months. N = 284. Questionnaire on Responsible Gambling based on responsible gambling promotional materials. Random phone calls. (2) Focus groups. Six men, 19 women. Aged 21–63. 11 were casino employees. 21 had gambling experience. Recruited through newspaper advertisement. Thematic analysis conducted	(1) Large number of active gambles adopted positive RG practices. 77.3% set upper limit of gambling amount. Many didn't practice positive habits however (37.9% didn't set a time limit). 31.4% thought chance of winning could be enhanced by studying the trend. More than 7% of gamblers would gamble with borrowed money. (2) All had high acceptance of gambling activities, but had strong stigmas against disordered gamblers. They were aware of government RG campaign, but only had superficial knowledge of RG practices	(1) Overall, RG practices were largely adopted in Macao, though plenty of room for improvement. (2) Socially in Macao, gambling is common and publicly acceptable. Small-stake betting was considered entertainment and did not induce much emotional distress when losing, therefore is often considered RG. There were negative attitudes towards disordered gamblers however (characterised by large stake betting). Problems with RG implementation included mistrust towards government, casino operators, and NGOs
Turner et al.	2018	Evaluate an interactive tutorial presented to problem gamblers, and gambling treatment counsellors, intended to help educate on the design of video slot machine types of electronic gambling machines	52(?) participants. 26 clients (55% male) and 25 counsellors (40% male). 16 additional counsellors completed evaluation questionnaire. Paired t-tests to compare time 1 and time 2 data. T-tests to compare counsellor and client scores. Effect sizes compared between group and individual sessions. MEASURES: REKT—how well do people understand concept of raondom chance. 9-questions directly relating to tutorial. 10 items evaluating the tutorial	Only clients in the individual group improved REKT scores. Group clients and all counsellors had no significant difference. Significant changes to some individual items were noted however	The tutorial improved knowledge of random chance amongst clients, especially those in individual sessions. No change was noted in the counsellor group, possibly due to higher pre-test scores. The tutorial was positively evaluated by both clients and counsellors
Turner et al.	2020	Examines the relationship between traumatic brain injury (TBI) and problem gambling in adolescents	Children aged 11–20, 52% female. N = 9198. Logistic regressions controlling for sex, year level at school, hazardous drinking, and suicidality	Problem gambling was associated with a history of TBI, even after controlling for confounders. Problem gambling showed a significant relationship with being male, hazardous drinking, and suicidality	While this study suggests a link between TBI and problem gabling, more research is required to determine whether there is a causal relationship, or the potential implications for prevention and treatment
Turner et al.	2021	Determine the efficacy of a tutorial designed to reduce the risk of harm to those return to gambling venues after exclusion periods	235 participants. 59% male. 104 in intervention group. 131 in control group. Baseline, 6-month and 12-month data was collected	Significant decrease in gambling and problem gambling compared to pre-exclusion in both groups. Sustained at 6 and 12 months. No additional effect was evident from the tutorial. Self-exclusion by itself was associated with sustained reduction in problem gambling	The self-exclusion program reduced problem gambling upon reinstatement, which was maintained at 6 and 12 month follow-up. Results from other studies are mixed, as self-exclusion is not consistently enforced by the gambling industry. There was no evidence that other types of gambling increased as a result of self-exclusion
van der Maas et al.	2019	Provide a scoping review of the use of internet-based interventions for problem gambling treatments and prevention, to provide an understanding of the current state of the field	Articles published between 2007–17, include intervention for problem gambling, use of internet to deliver the intervention	27 articles met review criteria. CBT was the most common for of internet-based intervention. Interventions were generally effective at reducing problem gambling score and gambling behaviours	A wide range of online interventions were covered, including text-interactions with counsellors, automated personalized feedback, and interactive CBT. They importantly reduced the barrier to accessing professional help. Samples lacked diversity, and little comparison with face-to-face interventions were included
Vegni et al.	2019	(1) To compare the presence, extent of, and characteristics of gambling in preadolescents, to see if it shows the same characteristics as adolescents and adults. (2) verify differences between those who gamble and those who don't. (3) Asses which risk factors detected in adolescents and adults in the literature can be predictive of preadolescent gambling	2475 eligible participants (M-age = 12.36, SD = 0.95), aged 11–14 years. Recruited from 47 schools across 18 regions of Italy. Gamblers were those who had gambled money playing any game in the last twelve months	UNIVARIATE ANALYSES: Gamblers were more likely males, older, and showed a higher record of inappropriate behaviour at school. Parents of these students had a higher proportion of gambling behaviour and family conflicts related to playing videogames or gambling. Non gamblers saw gambling as less of a risk or a habit than gamblers. LOGISTIC REGRESSION: gender, inappropriate school behaviour, parent gambling, troubles with parents relating to videogames, online gambling (without money) and age were all predictive of gambling	Even amongst preadolescents, gamblers are predominantly male. Players saw games of chance as less risky, and less habitual. Gamblers perceived games as fun and exciting, fitting the literature regarding the theme of sensation seeking and it's link with development of gambling disease. Predictive factors emerged including problems with school conduct, and problems with parents relating to video game use
Wall et al.	2021	Explore the relationship between different game types, patterns of play, diversity in gambling, and problem gambling	Recruited from Swedish national gambling helpline, those who completed online survey. 7463 eligible responses. 78% male, majority in metro regions. 10 game types were identified. Clusters were identified based on game types. 7 clusters identified: casino/betting/poker; casino/electronic machines; horses/lottery; online casino; online sports; online sports/casino; diverse (high on all game types). For all game types, an increase in problem gambling score was observed when more game types were also being played	Increased diversity in gambling was associated with higher problem gambling levels, though this effect varied by game type. High probability of online casino gambling was associated with the highest problem gambling	Regulation and prevention can focus on specific game types, especially online casino games which have the strongest association with problem gambling. Reducing the diversity of gambling options may reduce the impact of problem gambling
Wang et al.	2021	Investigate predictors of sports gambling amongst college students using the theory of planned behaviour. (2) Compared differences between recreational and problem gamblers	Using theory of planned behaviour. Survey data collected from 334 college students. Six constructs examined: attitude towards sports gambling; subjective norms (i.e. family/friends think it's okay/normal); perceived behavioural control; sports gambling intention; sports gambling behaviour; problem gambling. Data analysed using structural equation modelling	Intention and perceived control were the key predictors of sports betting. Intention to bet was highly predicted by attitude and subjective norms. The moderating role of gambling addition was included in analyses. For higher-risk gamblers, intention to bet was predicted by attitude and subjective norms. For problem gamblers sports betting was predicted by intention to bet and perceived behavioural control	Attitude towards sports gambling was the most significant antecedent of behaviour intention, indirectly predicting sports gambling behaviour. This highlights the importance of attitude formation and change amongst college aged individuals for prevention/reduction in gambling behaviours. Subjective nors also significantly influence intention towards gambling. This is in both directions, family and peer influences can be preventative, or increase intention towards sports gambling
Wardle et al.	2019	To address knowledge gap in relation to gambling behaviours, cultures and experiences of migrant groups	PRISMA with PICO	Reasons and motivations for gambling: Acculturation; advertising and availability of gambling in the new country. Protective factors: recent migrants more likely to disapprove of gambling citing too many venues; religious and moral tenets with religiosity potential protective against PG but not acculturative stress	
Wejbera et al.	2021	(1) Identify psychosocial risk factors for gambling disorder. (2) Does gambling disorder vulnerability differ between migration generations. (3)what is the mental and physical health burden of GD. (4)do risk factors and health burden differ between male and female gamblers	Large sample, cross-sectional. 11,875 participants (51.2% males, 40–80 years M = 59.2, SD = 10.8). Descriptive analyses and logistic regression models with probably gambling disorder as dependent variable	235 individuals with probably GD(2.1%). PSYCHOSOCIAL: Higher proportion male. GD group had Higher daily stress and more social support, more stressful life events and individual stressor, and less likely to have an educational degree. Men with GD were younger, more likely to be single, lower SES, and less educated. Those differences were not notable in females. HEALTH BURDEN: Probably GD group had higher depression, general anxiety, social anxiety, panic disorder, with worse self-rating of mental health. More likely to be smokers, be sedentary (high screen time), and worse self-rating of physical health. REGRESSION: Strongest predictors were 1st generation migrant, and being male. Other significant predictors were smoking, screen time, daily stressors, lower age, lower SES	As study was cross-sectional, whether poor mental health, life stressors etc. is a product or cause of gambling disorder is unclear. Gender differences were stark, with most predictors being related strongly to being male
Werner et al.	2020	Examine the relationship of alcohol, cannabis, and tobacco initiation, in relation to onset of gambling	Sample 44.4% white, 55.6% African American, from Missouri, USA. 767 participants. Descriptive analyses and regression analyses conducted	Sports betting, craps were higher for African Americans. For whites, alcohol was more likely to have proceeded gambling, or tobacco/alcohol use to occur without gambling. For African American males and females, gambling was more likely to have preceded or occur at the same age as alcohol use. ASSOCIATIONS: for African American males, cannabis and alcohol significantly increased the hazard for gambling initiation. Conduct disorder significantly increased the hazard for white males. For African American females, conduct disorder significantly increased the hazard, while white female cannabis initiation increased the hazard. Gambling increased the hazard for other substance use in varying significances between different demographic groups	Racial differences were highlighted. Conduct disorder was associated with increased risk of gambling initiation (except for white females). Overall, increased risk of adolescent gambling is linked to early substance use and early conduct disorder across gender and race groups, though the substance type varied across groups
Whiteside et al.	2020	To systematically search and review the literature relating to interventions designed for Indigenous populations that seek to prevent or address gambling harm, to support the design of new programs		Only four articles were identified for inclusion. Only one provided outcomes data, which was inconclusive, and one described three separate interventions. Most described community-led approaches informed by cultural and emancipatory principles. Currently insufficient data to inform interventions aiming to prevent and address gambling harm for Indigenous peoples	Urgent need for intervention data related to Indigenous peoples
Wieczorek et al.	2019	Explore gambling habits within the homeless population (staying at rehabilitation shelters or night shelters)	Self-report survey conducted at rehabilitation and night shelters in Warsaw. 690 respondents met criteria (> 18yrs, lack of stable residence, currently in shelter, capable of giving informed consent). Descriptive analyses and Logistic regression was conducted. Sample 90% male, Mean age 49.5, SD = 12.8	30.2% had at least some signs of problem gambling. 11.3% met criteria for problem gambling. Most common symptoms of those with problem gambling were trying to win back money they lost and gambling caused financial problems. Problem gambling was more of a problem for men than women, and for younger people. Lotteries were the most popular type of game (60% of respondents had participated within the last 12 months) among homeless, though for problem gambles, slot machines and private card games were also very popular	Problem gambling in the homeless population was 16 times higher than in the general population of Poland. Highlighting importance of addressing problems for the homeless. Especially young homeless see gambling as a means to obtain money. High rates of lottery gambling could be due to low cost of tickets, and ease of purchasing/many purchase points. For problem gamblers, slot machines are the most popular gambling method, and private card games become much more popular
Williams et al.	2022	To provide a profile of Canadian Indigenous gambling and problem gambling using the 2018 Canadian Community Health Survey (CCHS) (n = 23,952 adults; 1,324 Indigenous) and an online panel survey of 10,199 gamblers (n = 589 Indigenous)		The relative popularity of different types of gambling was similar between Indigenous and non-Indigenous samples. However, there was higher Indigenous participation in electronic gambling machines (EGMs), bingo, instant lotteries, overall gambling and a higher rate of problem gambling (2.0% versus 0.5%). Variables predictive of Indigenous problem gambling were EGM participation, gambling fallacies, having a mental or substance use disorder, sports betting, and male gender. Compared to non-Indigenous problem gamblers, Indigenous problem gamblers had higher substance use and lower impulsivity	Variables predictive of Indigenous problem gambling were the same ones predictive of problem gambling in all populations, with elevated Indigenous problem gambling rates primarily being due to elevated rates of these generic risk factors. Many of these risk factors are modifiable. Particular consideration should be given to reducing the disproportionate concentration of EGMs in geographic areas having the highest concentration of Indigenous people and ameliorating the disadvantageous social conditions in this population that are conducive to mental health and substance use problems
Williams et al.	2021	(1) Identify the demographic profile of gamblers and problem gamblers in Canada. (2) Identify the demographic, mental health, and gameplay patterns most strongly associated with problem gambling. (3) Identify policy implications that arise from these findings	Large scale survey of Canadian population. 23,952 eligible responses (aged 18 +). Gambling habits, substance abuse/mental health data, and demographics were collected. Logistic regression conducted	Highest rates of problem gambling was in males, those aged 18–29, income bracket of 40-80 k, having below a bachelors degrees, being indigenous, and living in the prairie provinces (rural). Lowest rates were amongst females, those 50 + , incomes > 150 K, having a university degree, those with Latin heritage, and living in Ontario or BC (less rural). Use of electronic gambling machines was the key predictor of problem gambling, though other factors had an additive effect. The two provinces in Canada that don't allow electronic machines outside of dedicated gambling venues (i.e. casinos) had the lowest rates of problem gambling. Those with indigenous heritage had the greatest electronic machine use (compared to other race/ethnicities). Different gambling habits were associated with different demographics	Use of electronic machines was the biggest predictor of problem gambling. However the study was missing data for other key predictors of problem gambling which have been identified in other studies so lacks that context
Zhai et al.	2017	Examine the relative and additive effects of perceived gambling in family and peers, on adolescents at-risk/problem gambling (and binge drinking)	Invitations sent to all high-schools in Connecticut. 4523 adolescents surveyed. 51.6% male, aged 14–18. Problem gambling examined in logistic regression against perceived family and peer gambling	53.9% reported low-risk gambling, 17.4% at risk gambling, 10.4% problem gambling. Worry over family gambling or perception of peer gambling was associated with higher likelihood of at risk and problem gambling compared to low-risk gambling. Those who identified family concerns but not peer gambling were just as likely to have at risk or problem gambling than those with both family and peer concerns	Perceived concerning or excessive gambling within either family or peer groups is associated with greater likelihood of at risk and problem gambling. Family concerns appear to be a greater risk factor for at risk and problem gambling than peer gambling, amplifying the effects for individuals who experience gambling in peers
Zhai et al.,	2021	Examine relationship between problem gambling severity and behaviours with lottery-purchasing status	Invitations sent to all high-schools in Connecticut. 1517 with current gambling and relevant measures completed. 65% male, aged 14–18 years. Logistic regression conducted	Lottery purchasing adolescents saw gambling prevention measures as not important compared to peers. Lottery purchasing was associated with at risk and problem gambling, pathological gambling, and gambling disorder	Literary purchasers had a greater association between at risk/problem gambling, and gambling alone, as well as with internet and machine gambling. Those who gambled not on the lottery were more likely to do so with friends and adults
Zhang et al.	2020	Test the relationships between purpose in life, psychological flourishing, and self reported gambling disorder symptoms	Convenience sampling was used to recruit at time 1. Those who agreed to follow up were contacted via phone or email. 283 Chinese university students were interviewed with data at baseline and 12-month follow-up. (39.6% males; age 18–27 with M = 20.47, SD = 1.15)	Gender was positively associated with gambling disorder symptoms at both time points. Gambling symptoms at time 1 were negatively associated with psychological flourishing at T2. Psychological flourishing at T1 was negatively associated with Gambling symptoms at T2. Purpose in life was negatively associated with gambling symptoms and positively correlated with psychological flourishing. Purpose in life at T1 significantly predicted gambling disorder symptoms at Time 2 (p < .001). GD at T1 did not predict anything at T2	The findings support a unidirectional relationship, at least in the formative years of gambling disorders. Lacking purpose in life predicts symptoms, but having symptoms does not predict a lack of purpose or lack of psychological flourishing. Results suggest that having a purpose in life can mitigate vulnerability to gambling disorder
Zhao et al.	2018	1) To examine the prevalence of mobile gambling among adolescents, its association with problem gambling, and other forms of gambling. (2) To investigate the predictive effects of perceived parental and peer disapproval of gambling and perceived risk of harm related to mobile gambling behavior	PARTICIPANTS: 6,818 participants (3,341 M; 3,224 F, 253 unspecified) from Grades 7 through 12, aged 10 to 19 yrs. MEASURES: Past-year mobile G and other forms of G, G probs, perceived risk of harm-related to G, perceived peer and parental disapproval of G	**Prevalence of G and Mobile G:** 31% G in past year. 5% via mobile device. M were 3.7 times more likely to G and use mobile device for G than F. While younger adol (Grades 7–9) were associated with lower mobile G involvement, older students (Grades 10–12) were 1.93 times more likely to engage in this activity. **At-Risk G**: 7.5% were at-risk of G-related problems. M > incidence of being at-risk for G probs than F. **Assoc of Mobile G and At-Risk G**: Past-year mobile G frequency was significantly associated with at-risk G.. Participants G on mobile devices equal to or greater than once a month were significantly more likely to be identified as at-risk G than youth who engaged in mobile G less frequently. **Mobile G Association with Other Forms of G:** Mobile G at least monthly was significantly correlated with a higher frequency of most questioned forms of G Behavior, except for playing cards for money, buying scratch-offs, and betting money on Keno, when controlling for effects of the other predictors. **Predictive Effect of Perceived Risk of Harm, Parental and Peer Disapproval**: Adol perceived G as a slight to moderate risk behavior. Students perceived greater disapproval of G from their parents than peers. After controlling for age and gender, parental and peer disapproval and perceived risk of harm still significantly predicted past-year mobile gambling frequency. M are 4.16 times more likely than F to report G with their mobile device on a monthly basis or more. Odds of mobile G more than once a month being 1.31 times greater for every increase in grade year. Only parental disapproval of G was found to be a significant predictor of past-year mobile G engagement, with one unit of increase in parental disapproval decreasing the odds of mobile G on a monthly basis or more by 2.27 times	G prevalence rate of 5% for mobile G relatively low compared to overall adol G rate (noting online G is illegal in Ohio and adol can't access credit cards). But rate is 2% higher in a study were online G is legal and commonplace. Strict age-related identity checks and rigid verification procedures need to be adopted. Regular mobile G (at least once a month) are 13 times more likely than non-regular G to be at risk of experiencing a G prob. Consistent with previous research and suggests higher prevalence rates of disordered G if mobile G. Regular mobile G was found to be highly associated with regular engagement of multiple forms of G, especially with online poker, daily fantasy sports, and fantasy sports. Gender remains a significant factor associated with adolescent mobile G
Zhou et al.	2019	To assessing the effectiveness of a gambling prevention programme, GameSense, in modifying gambling cognitions and intentions among university undergraduate students of diverse ethnicities	PARTICIPANTS: Undergraduate students (N = 122). METHOD: randomly assigned treatment participants completed the programme and then played a gambling game in which they could win tokens for a desired prize. Control participants played the game but did not receive the prevention programme. DESIGN: The two Treatment (GameSense vs. Control) and three Gambling Outcome conditions (win, lose, break-even) were manipulated between subjects in a 2 × 3 randomized groups design. Participants were randomly assigned to one of the six treatment conditions	Programme participants showed increased knowledge about gambling, increased resistance to gambling fallacies, and fewer immediate and future intentions to continue gambling regardless of how much they won or lost, compared to the no-treatment control group. This is consistent with previous research. Participants who completed the programme reported a lower desire to continue playing, decreased the time they wanted to continue gambling, and wanted to play fewer minutes at the conclusion of the four gambling trials than did participants in the control condition. GameSense participants’ future intentions were not significantly influenced by whether they won or lost; whereas control participants in the winning condition, on the other hand, wanted to repeat the study in a month (without additional credit) and to participate in a similar game sometime in the undetermined future	The GameSense programme is effective in altering cognitions about gambling and immediate and future intentions to continue gambling. LIMITATIONS: Students. Few if any with G problems. Approximation of a real gambling event. Trial game was game of chance therefore not similar to gambling

Results were recorded and arranged according to the research questions and the project focus on a public health perspective with relevant themes from the literature: risk and protective factors; secondary prevention/harm reduction; primary prevention/early intervention; target population group; public health approach; and tertiary prevention/treatment.

## Q1. What is known about programs designed to address gambling-related harm?

Evidence related to gambling-related harm prevention can broadly be categorised according to levels of prevention: primary, secondary, and tertiary prevention. For readability, findings will be presented accordingly.

### Primary prevention

In large part, evidence around primary prevention or early intervention for gambling-related harm programs were school or university-based and tended to be educative programs targeting cognitive distortions or misconceptions around gambling risk [20–33]. Several literature reviews, some systematic, referred to successful primary prevention programs in schools. They found that programs that were multifaceted and addressed context as well as individual attitudes and beliefs were more effective at reducing at-risk or problematic factors, like misperceptions of gambling and illusions of control, in the longer term [22,34,35]. Grande-Gosende et al., (2019) conducted a systematic review of evidence related to programs designed for college/university students and found that programs that incorporated Personalised Normative Feedback were the most effective in terms of reducing at-risk or problem gambling among young men.

Several included studies provided primary data related to program outcomes and effectiveness [29–32,36]. Donati et al., (2018) investigated gambling-related cognitive distortions (or mindware distortions, where mindware is the rules, procedures and strategies derived from past learning used to solve problems [37]) confirming effectiveness of tailored interventions to address them. Recommendations suggested that prevention strategies address mindware problems as they can be considered predictors of gambling-related cognitive distortions. St-Pierre et al., (2017) examined the impact of targeting Negative Anticipatory Emotions and key Theory of Planned Behaviour (TPB [38]) constructs in modification of gambling beliefs, intentions and behaviours in an adolescent gambling tool—the Clean Break video [32]. Results indicated a small but statistically significant increase in positive gambling attitudes and positive peer and family subjective norms at post-intervention and follow up, where positive is indicative of anti-gambling.

In terms of causal pathways, many of the included studies examined relationships between sociodemographic factors of individuals and either risk of developing or presence of problem gambling. However, Dussault et al., (2019) examined current poker playing adolescents to identify groups according to sociodemographic and gambling-related characteristics [33]. Findings from that study indicated that adolescents who gamble were more likely to respond to interventions designed to address ‘adult’ gambling problems as both groups of gamblers shared sociodemographic and gambling-related characteristics. Interestingly, Dussault et al. (2019), also found that adolescents who played simulated poker did not exhibit or were not at risk of developing problem gambling.

Primary prevention programs were focused on raising awareness of the potential harm of gambling, mostly to young people. There was a lack of evidence of effective education programs at a whole of community level.

### Secondary prevention

In general, evidence around secondary prevention programs targeted individuals already experiencing gambling-related harm and tended to refer to ‘responsible gambling’ strategies [39–49]. Ladouceur et al., (2017) conducted a systematic review to identify empirically grounded responsible gambling studies to inform evidence-based, effective responsible gambling programs. Little empirical evidence was available from the 105 included citations in the Ladouceur review on which to develop intervention frameworks, and no data beyond 12 months was provided [50–52]. However, five responsible gambling strategies and the relative effectiveness of each were discussed, and outlined as follows [53]. *Self-exclusion*, where individuals participate in a program to be banned from specific gambling venues [54], was poorly adopted but more successful in the longer term if combined with individualised follow-up. Indicators of *responsible gambling behaviour,* where the individual exercises self-restraint, were difficult to track due to lack of access to universal data. However, *gambling frequency*, defined as expenditure and duration of gambling, was strongly linked to likelihood of problem gambling. *Monetary limit setting* — establishing a maximum spend either per bet or for each gambling session — was shown to be more effective than time limiting strategies, with some variability when limit setting is self-determined. *Venue training* programs were shown to be minimally effective as staff were poor at recognising patrons at-risk of gambling-related harm, with obvious disparities in self-reported Problem Gambling Severity Index (PGSI) scores and staff estimates. Staff were also unlikely to discuss issues of problem gambling with patrons due to personal discomfort. Secondary prevention was targeted at directly addressing the individual’s behaviour around gambling.

Other studies focused on the impacts of personalised feedback [51,52,55,56] and/or personal contact between counsellors and people who gamble [57–62]. Jonas et al., (2020) found that personal contact with counsellors in conjunction with other responsible gambling type interventions had the greatest impact on longterm reduction in problem gambling scores. In terms of impacts of personalised feedback for people who gambled, the most effective interventions were those that provided non-judgemental messaging based on gambling facts and amounts spent [55,63–66]. The effectiveness of computerised interventions to reduce gambling harm have been examined [67–70]. Findings indicated that ICT based interventions had widespread potential for harm reduction; however, those interventions that incorporated personalised feedback were the most effective.

Two included studies examined evidence of a public health approach to gambling harm, identifying a relative paucity of empirical evidence [71,72]. McMahon et al., (2019) conducted an umbrella review of systematic evidence around prevention and harm reduction and how these varied across sociodemographic groups. Findings indicate the majority of programs were focused on individual harm reduction, and the varying degree of impact that has on outcomes. Evidence on impacts of supply reduction was limited, with the authors identifying the urgent need for adequately focused research on harm reduction in relation to gambling. Peterson et al., (2021) also identified the paucity of empirical data around effective gambling harm reduction, examined other harm reduction fields like blood borne viruses (BBV) and smoking and outlined protective behaviour strategies (PBS) that could effectively translate to gambling harm [71]. Peterson, et al., (2021) argue that PBS effectively reduced negative consequences associated with gambling behaviours but outlined the pressing need for gambling specific evidence.

### Tertiary prevention

Research on psychological interventions designed to address pathological or problem gambling is abundant. A Cochrane Review published in 2012 detailed successful therapies [73]. While outside this review timeframe, a wealth of psychologically focused research has been published since 2012, with an ongoing interest in the individual and problem gambling [29–31,56,74–76]. Research tended to explore aspects of the individual that either predisposed them to problem gambling or identified various behavioural aspects that could be measured, targeted, and adjusted such that problem gambling was avoided or reduced. Problem gambling in this context is behaviour characterised by difficulties in limiting money and/or time spent on gambling which leads to adverse consequences for the gambler, others, or for the community [77]. Dowling et al. (2021) discussed the benefits of examining the relationship between positive outcomes expectancies (the expectation that one stands to gain from gambling) and gambling behaviour finding no reciprocal relationship between expectancies and gambling behaviour. A possible moderating effect of positive emotional states and the importance of social supports as a moderator of monetary expectancies was demonstrated [76].

There was limited longitudinal data beyond 12 months to assess effectiveness of interventions on lasting behaviour change. Only one included study [78] provided follow up data after eight years in relation to differences between voluntary excluded gamblers compared to forced excluded gamblers with all excluded gamblers either stopping all gambling (20.5%) or reducing their gambling (66.5%) at eight years. However, this was not consistent across all gambling venue types with no difference in gambling behaviours seen in those who gambled in gambling halls (an area within a premise where games of chance are organised and conducted [79]).

In keeping with the recent shift in the gambling discourse away from problem gambling and pathologisation of individuals who gamble, Blank et al., (2021) conducted a systematic mapping review to capture evidence from interventions designed to reduce gambling-related harm [80]. Included reviews were divided into those reporting universal/primary prevention interventions and those evaluating interventions for individuals at high-risk of harm. There was support for primary prevention through supply reduction that included screen pop-up messaging, despite a general lack of empirical evidence in the literature. Therapeutic interventions were shown to be effective in the short-term with minimal to no long-term data. Blank et al. (2021), pointed out the limitations of programs referring to problem gambling or problem gamblers in addressing gambling-related harm at a societal or population level. They argued that focusing on ‘problem-gamblers’ fails to address underlying causes of harmful behaviours and the influence of gambling policy and supply. They argued for the pressing need for evidence to support specific types of interventions for gambling-related harm reduction.

While programs designed to address problem gambling exist at the primary, secondary and tertiary levels of prevention, they are focused on individual behaviour and in specific cohorts.

## Q2. What evidence is there of impact of gambling-related harm prevention programs on priority population groups?

Priority population groups are those groups that face inequitable burden of high risk activity (gambling-related harm) when compared to the general population, such as Indigenous peoples, older people, and people who live outside the metropolitan area [81]. We based our selection of priority groups on current national, non-gambling specific, priority groups as outlined by the Australian Institute for Health and Welfare (AIHW) [81]. There is limited evidence on the impact of gambling-related harm prevention programs on priority population groups, with only evidence related to Indigenous peoples, older people, young people, LGBTIQA + and migrants captured in this review.

### Indigenous peoples

Two articles referred to harm reduction programs specific to Indigenous peoples [82,83]. Whiteside et al., (2020) conducted a systematic review of interventions in Indigenous gambling identifying four articles, only one of which provided (indeterminant) outcomes data. This is supported by Saunders et al., (2021) who also identified a dearth of empirical evidence to inform the design of policy and interventions for Indigenous populations.

### Older People

Evidence related to programs specifically designed for older people (recorded as aged 65 years and older) was limited in the literature, but still instructive [84–86]. Skinner et al., (2018) operationalise existing best-practice frameworks [85] to produce guidelines for use by practitioners, patients, families, policy makers, and concerned others. These guidelines focus on five key areas including person-centred and family-focused care, screening and assessment, secondary prevention and early intervention, tertiary prevention and specialised treatment, and ongoing support and recovery services.

### Young people

Research on programs designed to address gambling-related harm in young people were predominantly focused on primary prevention / early intervention and were schools-based programs, as described earlier. McArthur et al., (2018) conducted a systematic review of interventions designed for primary and secondary prevention for individuals up to 18 years across multiple risky behaviours, including gambling that were conducted at the individual, family or school level [87]. Forty percent of included studies were conducted in school settings with findings indicating that schools-based programs were the most effective for addressing key risky behaviours with no clear suggestion as to why these programs were more effective. Evidence was minimal as to the effectiveness of interventions targeting families when compared to the effectiveness of broader focused programs [88,89].

### Migrants

While the experience of migration may impact people’s gambling behaviour, we acknowledge that other aspects of cultural identity beyond migration may also be a factor. In this context, migrants are understood to be first-generation migrants who have recently arrived in the country in which the study took place [90]. Drawing from Wardle et al., (2015), we acknowledge the distinction between migrant status and ethnicity, and the obvious overlap between ethnicity and ethnic cultures. There was a general paucity of evidence specific to migrants; however, one included study provided primary data related to a dedicated public health intervention specifically targeting problem gambling across an entire city [91]. Elbers et al., (2020) examined the impact of a collaborative approach across statutory and volunteer services to reduce problem gambling in one UK city. Within that study, migrant groups were identified as being of particular interest due to the presence of the ‘harm paradox’ [92]. Drawing from work in alcohol and other drugs, Wardle et al. (2019) explain that the harm paradox is seen in certain population groups with particular characteristics (lower socioeconomic position, for example) where those population groups are less likely to gamble but if they do gamble, they are more likely to experience harm.

## Q3. What is known about the risk and protective factors for gambling-related harm, particularly in priority population groups?

### Overview

We use the definition of risk and protective factors in line with Billi et al., (2014) where “risk factors are attributes associated with the development of gambling problems, and protective factors are attributes that provide resilience or protection from the development of gambling problems”. The evidence related to risk and protective factors for gambling-related harm largely spoke to risk factors.

Risk and protective factors for gambling-related harm are similar to other public health issues like alcohol, tobacco, physical activity, obesity, and blood borne viruses. Evidence outlines strong associations between sociodemographic characteristics and increasing gambling severity and levels of gambling-related harm [94–105]. Specific sociodemographic factors were identified across numerous studies and include a mix of risk and protective factors such as increased educational attainment [106–111], relative deprivation [112–114], parental engagement and role modelling [115–124], peer group behaviour [125], and location of residence relative to numbers of gambling venues [126,127]. Coincidental substance use and/or abuse was also identified as a strong risk factor for gambling, both in terms of frequency and severity (amount of money lost per event) [128–134], along with increased diversity of gambling product use and frequency [135–139]. The normalisation of gambling and/or sports betting was also explicitly described as a risk factor in gambling activities in pre-teens. Nyemcsok et al. (2021) argued that increased awareness of marketing influence around sports betting was a key factor in predicting reduced gambling activities of young people. Roderique-Davies et al., (2020) also argued that exposure to gambling advertising in the absence of awareness of marketing influence, is a strong risk factor for gambling urge.

Activities like gaming (the use of video games), and particularly purchasing loot boxes (purchasable video game content with randomised rewards) in computer games, has recently been identified as a significant risk factor for developing problematic gambling [142]. These are sometimes described as “gateway activities”. Drummond et al., (2020) found in their international survey that individuals who regularly purchased loot boxes while gaming exhibited symptoms of problem gambling and changes in mood.

The psychological literature provides a wealth of information about various individual characteristics that may be associated with developing problematic gambling [75,76,143–156], and how these may be experienced across various life stages and related to other risky behaviours [98,157–163]. The interaction of comorbid mental health issues has been clearly associated with gambling-related harm [164,165], with a strong relationship between depression, anxiety and at-risk or problem gambling. Again, while much of this research demonstrates correlation, it is impossible to definitively outline causation given the socially constructed and complex nature of gambling and gambling-related harm [166]. The interaction between traumatic brain injury (TBI) and problem gambling (PG) was also examined in two separate studies by Turner et al., (2020 & 2019), finding a link between TBI and PG but no evidence of a causal relationship [167,168].

While much of the included literature described risk factors, Dowling et al., (2021) examined the protective nature of positive mental health characteristics like general coping, emotional support, spirituality, interpersonal skills and global affect. They found that emotional support in conjunction with a strong sense of self were protective against developing problem gambling [75]. Rash and McGrath (2017), who also examined potential protective factors for young adult, non-gamblers, identified religiosity and non-alcohol consumption as potential protective factors for gambling abstinence [169].

There is increasing evidence about the relationship between media campaigns and intentions to gamble across various demographic groups [170,171]. Bouguettaya et al., (2020) conducted a meta-review to establish the relationship between gambling advertising and gambling-related attitudes, intentions, and behaviours. Findings from the 24 included studies demonstrated a strong positive relationship between exposure to gambling-related advertising and intentions to gamble. Once again, the lack of longitudinal behavioural data was cited as a limitation to the strength of the finding and a key future research area.

#### Priority populations

Like evidence that refers to targeted interventions, this review captured limited empirical evidence of risk and protective factors for priority populations within the identified search period. Available evidence was specific to Indigenous peoples [172,173], older people, migrants, women [173], and the LGBTIQA + community.

### Indigenous peoples

Williams et al., (2022), in an examination of Canadian population-wide health data, identified several predictive indicators of Indigenous problem gambling. Specifically, use of electronic gambling machines (EGMs), the presence of gambling fallacy beliefs, having a mental or substance use disorder, sports betting and being male were likely to contribute to problem gambling in Canadian Indigenous peoples. Sharman et al., (2019) conducted a systematic review of psychosocial risk factors in disordered gambling across identified vulnerable groups, where disordered gambling is an umbrella term for gambling related harm. For Indigenous peoples, a history of discrimination and dispossession are thought to increase vulnerability to gambling disorder. Specifically, however, gender (being male), childhood exposure to gambling, having friends and/or family who gamble and being socially marginalised were identified as risk factors in conjunction with Indigenous status. Like Williams et al., use of specific gambling products increased vulnerability to disordered gambling and increased frequency of gambling, and fallacious beliefs were also identified as risk factors [174]. MacLean et al., (2019) investigated the social practice of gambling for two Australian Indigenous communities, finding similar antecedents to gambling as Williams et al. [172] and Sharman et al. [173] However, the socially embedded nature of gambling was identified as a key limiting factor in the relative impact of any harm reduction program. This is supported by Gupta et al., (2021) in their study of the impacts of gambling on the person who gambles as well as non-gamblers because of the socially embedded nature of gambling for both Indigenous and non-Indigenous peoples.

### Older people

Two studies explicitly discussed risk factors for gambling-related harm in older people [99,100]. Heiskanen and Matilainen (2020) argue that significant historical changes to the nature of gambling from being for ‘a good cause’, like church raffles, to the current context of commercial gambling must be considered when viewing gambling-related harm in older people. Moreover, the deterministic understanding of gambling problems as a personal flaw can prevent recognition of problem gambling and subsequent help seeking. These risk factors are considered stronger indicators of problem gambling than gender and educational attainment. Elton-Marshall et al. (2018) investigated the impacts of loneliness and social isolation among older people, suggesting that older people who socialise and who were married were far less likely to have high Problem Gambling Severity Index (PGSI) scores than older people who were divorced or single with limited social networks.

### Migrants

Wardle et al., (2019) provided a rapid review of literature related to gambling-related harm affecting migrants and migrant communities, outlining various motivators to gamble. In particular, acculturation and acculturative stress, the impact of advertising and level of access to gambling in the new country were identified as risks for increasing gambling-related harm [177]. Protective factors were identified as levels of disapproval of gambling among migrants, and religious and moral tenets. However, this may be outweighed by acculturative stress, availability of gambling and the impact of the immediate social environment [92]. Importantly, Wardle and colleagues were careful to point out the ‘harm paradox’ experienced by migrants. This is supported by Bramley et al., (2020) in their investigation of barriers and enablers to support services from the perspective of migrants and the organisations supporting them [178]. According to Bramley and colleagues, migrants were at greater risk of gambling-related harm due to a lack of a social safety net in the form of family and friends in their new country. The inability to access informal support and the impact of gambling loss may have broader ramifications than they do for local gamblers [179]. Moreover, the tendency for migrants to work in a cash economy may enable individuals to binge gamble, potentially driving the self-perpetuating ‘chase mode’ that can be exacerbated via limited ability to secure money within formal banking systems.

### Women

There has been little research on women and gambling [180,181]; however, there is increasing evidence of the lived experience of gambling-related harm among women [182]. Sharman et al., (2019) in their systematic review of psychosocial risk factors referred to women as a discrete group, identifying availability and access as well as the social nature of gambling as risk factors for disordered gambling. Women seeking treatment were also more likely than women not seeking treatment to present with co-morbid psychological conditions like anxiety, depression and mood disorder, and substance-use disorder. Experiencing trauma and abuse, living in a violent relationship, having low self-esteem and poor coping skills were all shown to be of high prevalence. Aggression in female adolescents was hypothesised to be indicative of future risky gambling [173].

### *LGBTQIA* + *community*

Only one study referred to risk factors evident in the LGBTQIA + community, specifically trans and gender diverse (TGD) teenagers [183]. Rider et al., (2019) conducted a prevalence study of TGD youth in comparison to cis gender adolescents finding that assigned male sex at birth, regardless of gender transition, was a positive risk factor for developing problem gambling behaviours.

## Discussion

This rapid synthesis evidence review included 166 peer reviewed studies about programs designed to address gambling-related harm. Most of the included studies referred to individual risk and protective factors, like socioeconomic position, age, and ethnicity, of people who gamble and explored how those factors may have contributed to overall gambling outcomes. Although there is an emerging body of evidence dedicated to public health approaches to gambling-related harm, there is a clear gap in empirical research findings around effective interventions to reduce gambling-related harm, particularly from a public health perspective.

Public health approaches to high-risk behaviours examine the social and environmental elements around the individual that may contribute to the likelihood of experiencing harm [184]. Examining gambling-related harm as a social concept is relatively new in comparison to focusing on the individual in isolation from their social context [80]. Much of the evidence included in this review focused on programs designed to address ‘problem gambling’ while identifying predisposing social and individual factors. Public health approaches to harm reduction centralise the individual within their social context while actively examining the various social determinants influencing that individual [6]. Very few of the included programs that focused on the individual demonstrated efficacy in the absence of support structures put in place around the individual. Predominantly, the included evidence outlined behaviour change mechanisms without support for the individual in their social context. This is a clear omission as the success of public health approaches to other high-risk activities like alcohol and other drugs are well documented [184].

Gambling-related harm is inherently determined by social context and industry/commercial influence on individual behaviour and choices [185]. By taking a public health approach, programs depart from an addiction focus to appreciate the far broader group of people experiencing gambling-related harm but who do not reach thresholds for addiction [186]. Most importantly, harm reduction also includes the numerous ‘affected others’ who bear the secondary impact of gambling-related harm ^4^.

Evidence supporting a public health approach for gambling-related harm is still developing but increasing. Findings from this rapid review described programs designed to respond to individuals whose behaviour is described as problem gambling (tertiary prevention), with variable effect on gambling behaviours and the individual’s experience of harm and no overt broader harm reduction approaches. Harm reduction programs generally seek to achieve primary or secondary prevention. Removing supply (primary prevention) is less likely in the context of a community that has liberalised gambling; however, targeted programs for harm reduction (secondary prevention) are far more likely to reduce overall harm experienced at an individual, community or population level. Wardle et al., (2019) argue that any interventions designed to address gambling-related harm must incorporate key understandings of the commercial, policy and regulatory environment that contribute to gambling exposure and subsequent harm. Gambling harms are related to increasing social disadvantage, with those who are less able to afford the monetary loss experiencing greater harm. Given the complex interplay between supply, social factors and individual behaviour, a public health approach may offer a more effective mechanism to reduce gambling-related harm. To optimise harm reduction outcomes in a liberalised gambling environment, public health programs should be implemented on a larger scale.

## Conclusion

From this rapid literature review, two distinct discourses around gambling-related harm and effective interventions were apparent. First, a discourse around harm reduction and ‘responsible gambling’ that focuses on the ‘problem gambler’ as an individual, without recognising the broader context of commercial and social factors and harms. This discourse is seen most strongly in the tertiary prevention literature. Second, the smaller, yet emerging, discourse dedicated to preventing and reducing gambling-related harm that acknowledges the socially and commercially constructed nature of gambling and associated harms. This discourse was more likely to be to present in the primary and secondary prevention literature.

There was a clear paucity of empirical evidence related to effectiveness of programs that address gambling-related harm, particularly those that target primary or secondary prevention. By adopting a public health approach when addressing a complex issue like gambling-related harm, efforts to reduce harm are optimised are only possible in the context of sound evidence. Similar harm reduction areas like alcohol and other drugs equally rely on sound evidence to produce the positive outcomes evident in that field.

## Data Availability

No data were created or analysed in this study. Data sharing is not applicable to this article.
